# Soft tissue material properties based on human abdominal *in vivo* macro-indenter measurements

**DOI:** 10.3389/fbioe.2024.1384062

**Published:** 2024-05-24

**Authors:** Robin Remus, Christian Sure, Sascha Selkmann, Eike Uttich, Beate Bender

**Affiliations:** Chair of Product Development, Department of Mechanical Engineering, Ruhr-University Bochum, Bochum, Germany

**Keywords:** soft tissue biomechanics, inverse FEA, surface EMG (sEMG), abdominal wall, hyperelastic material properties, human abdomen, *in vivo* indenter measurements, FEBio

## Abstract

Simulations of human-technology interaction in the context of product development require comprehensive knowledge of biomechanical *in vivo* behavior. To obtain this knowledge for the abdomen, we measured the continuous mechanical responses of the abdominal soft tissue of ten healthy participants in different lying positions anteriorly, laterally, and posteriorly under local compression depths of up to 30 mm. An experimental setup consisting of a mechatronic indenter with hemispherical tip and two time-of-flight (ToF) sensors for optical 3D displacement measurement of the surface was developed for this purpose. To account for the impact of muscle tone, experiments were conducted with both controlled activation and relaxation of the trunk muscles. Surface electromyography (sEMG) was used to monitor muscle activation levels. The obtained data sets comprise the continuous force-displacement data of six abdominal measurement regions, each synchronized with the local surface displacements resulting from the macro-indentation, and the bipolar sEMG signals at three key trunk muscles. We used inverse finite element analysis (FEA), to derive sets of nonlinear material parameters that numerically approximate the experimentally determined soft tissue behaviors. The physiological standard values obtained for all participants after data processing served as reference data. The mean stiffness of the abdomen was significantly different when the trunk muscles were activated or relaxed. No significant differences were found between the anterior-lateral measurement regions, with exception of those centered on the linea alba and centered on the muscle belly of the rectus abdominis below the intertubercular plane. The shapes and areas of deformation of the skin depended on the region and muscle activity. Using the hyperelastic Ogden model, we identified unique material parameter sets for all regions. Our findings confirmed that, in addition to the indenter force-displacement data, knowledge about tissue deformation is necessary to reliably determine unique material parameter sets using inverse FEA. The presented results can be used for finite element (FE) models of the abdomen, for example, in the context of orthopedic or biomedical product developments.

## 1 Introduction

Understanding the functions and properties of biomechanical systems is a key success factor in computer-aided human-centered design (Neumann and Bender, 2022). The basis for this are robust and valid human body models, that allow to examine the effects of crucial biological or technical variables (Wolf et al., 2020b; Neumann et al., 2020). Product development involving user behaviors or properties of the human body has traditionally been an iterative and empirical process (Grujicic et al., 2010). CAE (computer-aided engineering) can help reduce development costs and time, as it enables early preclinical verification, ethical assurance, reduction of repetitive patient involvement, and quantification of mechanisms of action (Wolf et al., 2020a; Alawneh et al., 2022). However, the study and optimization of the interfaces between biomechanical and technical systems is particularly complex, as the transferred values are directly influenced by the interaction between the geometry and mechanical properties of both the human tissue and the technical system (Haug et al., 2004; Portnoy et al., 2008; Moerman et al., 2016; Sadler et al., 2018; Fougeron et al., 2023). In particular, this is the case in the lower part of the trunk, or the abdomen, as it consists of highly vulnerable tissue with great anatomical variations (Lamielle et al., 2008) and ensures vital bodily functions (Standring et al., 2016). Moreover, issues affecting the lower back, such as pain, are also associated with the soft tissues of the abdomen, which contribute to its stabilization (Hodges et al., 2005; Driscoll and Blyum, 2019) and unloading (Hodges et al., 2001; Stokes et al., 2010). In order to broaden the understanding of these biomechanical relationships and to improve the development of new aids using simulation models, the soft tissue behavior of the abdomen will be investigated in this study.

Recent simulation models that include the biomechanical behavior of the abdomen or parts of it differ in their implementing methods, scope, and degree of detail, depending on the requirements of their intended use case (Anderson et al., 2007; Hicks et al., 2015). Possible use cases encompass, for example, studies on 1) the effect of individual braces in scoliosis treatment (Périé et al., 2004; Clin et al., 2010; Sattout et al., 2016), or of lumbar orthoses (Molimard et al., 2019; Bonnaire et al., 2020) on the lumbosacral spine, 2) injury prevention in crash testing (King, 2018; Untaroiu et al., 2018; Grébonval et al., 2021) and stiff structure impact (Lee and Yang, 2001; Haug et al., 2004; Snedeker et al., 2007) or vertical impact load (Cox, 2020) studies, or 3) the load removal of the spine by increasing the intra-abdominal pressure (El-Monajjed and Driscoll, 2020; Guo et al., 2021). Another use case is modelling the interaction of organs (Misra et al., 2008), or the abdominal wall with surgical instruments (Hernández et al., 2011); these models are used for virtual surgical planning or support of education (Leong et al., 2022). Recently, authors analyzed the effects of muscular contractions on the biomechanics of the abdominal wall numerically (Pavan et al., 2019; Todros et al., 2020).

FEA is a standard approach in mechanics to calculate the reaction of structures to loads or interactions, but only few FE models exist that model the biomechanics for the whole abdomen (King, 2018). One aspect of biomechanical modelling are geometric shapes. While imaging data is accurate (Hayes et al., 2013a), it is not sufficient to derive material properties in terms of stress-strain data for all nonlinear responses of soft tissues (Sadler et al., 2018) and their interplay. In recent years, advancements in automatic segmentation methods, e.g., deep learning algorithms for exact and individual or statistical shape models, enabled the rapid generation of anatomical geometries (Sekuboyina et al., 2021; Ji et al., 2022; Ma et al., 2022). Geometrical data sets were generated using a large amount of imaging data, which include collections of single organs, vessels, and bones, as well as collections of body segments (Li et al., 2023).

While geometries are increasingly patient-specific and complex, the literature still lacks data on soft tissue material behavior, which is crucial for valid simulations (Kauer et al., 2002; van Loocke et al., 2006; Anderson et al., 2007). Thus, it is difficult to compile the complete data sets required for biomechanical modelling of the abdomen. Numerous invasive and non-invasive studies have been conducted to capture properties that go beyond the pure geometry of the abdomen, the abdominal wall that spans the anterior and lateral side of the abdomen, or the organs. Strategies used include, for example, the indirect and non-invasive estimation of intra-abdominal pressure (Tayebi et al., 2021) by measuring the tension of the abdominal wall via indentation (van Ramshorst et al., 2008; van Ramshorst et al., 2011). Functional responses, deformations, and kinematics of the abdominal wall were assessed during controlled muscle activity (Todros et al., 2019; Jourdan et al., 2022) or during upper body movements (Szymczak et al., 2012; Remus et al., 2023). Song et al. (2006) measured the *in vivo* elasticity of the entire abdominal wall during laparoscopic surgery and Szepietowska et al. (2023) investigated the non-homogeneous strain fields of external living human abdominal walls during peritoneal dialysis and breathing. Podwojewski et al. (2014) and Tran et al. (2014) examined abdominal walls subjected to air pressure loading *ex vivo*. Because the abdominal wall plays a crucial role in protecting the abdominal organs, moving the trunk, and stabilizing the lumbar spine (Hodges et al., 2005), other researchers used shear wave elastography (Tran et al., 2016; Wang et al., 2020) to estimate the elasticity of abdominal wall muscles, for example, to improve the treatment of hernias (Deeken and Lake, 2017). However, the biomechanical behavior of the abdomen and its elements depends strongly on the interplay of its elements. For example, muscle contractions and intra-abdominal pressure affect the biomechanics of the abdominal wall (Pavan et al., 2019), and the abdominal organs interact with neighboring organs and the walls of the abdominal cavity while undergoing large relative sliding movements (Haug et al., 2004). The mechanical properties of organs and their interactions were investigated under numerous aspects such as abdominal trauma (Viano, 1989; Lamielle et al., 2008; Ramachandra, 2016) or tool-tissue interactions (Carter et al., 2001; Davies et al., 2002; Tay et al., 2006; Sato et al., 2013).

Due to the lack of data on soft tissue behavior, in addition to an exact anatomical model, simplifications and assumptions, depending on the biomechanical modelling goals (Lee and Yang, 2001), are necessary. While it is known that abdominal muscles have a stiffening effect on the trunk (Hodges et al., 2015), it is rarely considered in abdominal interaction models and as it can only be determined *in vivo*. Modelling the macroscopic behavior requires geometry data and force-compression functions, but the amount of *in vivo* data present in literature is limited, e.g., due to ethical implications, costs, or expenditure of time. Therefore, when creating an interaction model, it may be necessary to identify own material parameters for the macroscopic behavior. A common approach for the abdomen is to assume one linear material parameter per body region and varying body postures (Périé et al., 2004; Cooper et al., 2019; Bonnaire et al., 2020), but among others, Huang and Zheng (2015) state that one parameter pure elasticity theory is not sufficient to meet the hyperelastic properties of soft tissue (van Loocke et al., 2006).

Indentation is a common approach to determine the macroscopic behavior and properties of tissue in different body regions (Huang and Zheng, 2015), e.g., buttock (Grujicic et al., 2010), thigh (Sadler et al., 2018), shank (Moerman et al., 2016), foot (Erdemir et al., 2006), or lower arm (Moerman, 2012). If impact time and total deformation are surveilled and limited, indentation is a safe and non-invasive method for *in vivo* measurement of hyperelastic soft tissue (Huang and Zheng, 2015). Zhang et al. (1997) and Marinopoulos et al. (2020) view material property measurement of soft tissue with an indenter as an inverse problem, and Davies et al. (2002) introduced an inverse engineering approach to solve the problem. However, extracting unique material parameters from indenter measurements can be difficult (Pierrat et al., 2018), because the inverse FEA might result in several parameter sets, which lead to similar experimental and simulation data with one or none being valid (Oddes and Solav, 2023). A possible approach to solve this problem is to measure the displacement of the surrounding surface, for example, via 3D digital image correlation (Solav et al., 2018), or an optical 3D deformation analyzer (Ahn and Kim, 2010). Conducting indentations and surface displacement measurements usually requires custom technical solutions (Lister et al., 2011) that must comply with strict safety regulations while producing adequate results (Marinopoulos et al., 2020).

The assessment of hyperelastic *in vivo* material parameters of the abdomen is extensive and requires appropriate measurements, which have rarely been reported. The aim of this study is therefore to determine the mechanical *in vivo* responses of the physiological abdomen under local uniaxial compression, taking into account the activation of trunk muscles during various controlled activities, and to derive characterizing hyperelastic material properties. For this purpose, we aim to

1) obtain continuous force-displacement curves from macro-indentation experiments, including the associated surface deformations, and
2) conduct inverse FE simulations for region-specific hyperelastic material model data.

We hypothesize that the abdominal tissue responses under local compressions show significant variations between regions and with varying muscle activities. Our approach is to determine unique material parameters using inverse FEA based on data that we have recorded non-invasively with an indenter developed for this purpose, including ToF 3D measurements. In order to evaluate the influence of muscle tone on the elasticity of the abdominal soft tissue, the tests were performed on multiple participants under both activation and relaxation of the trunk muscles. sEMG signals of the main trunk muscles were measured to monitor and evaluate activation. Measurements hopefully reduce inherent variability and errors due to uncertainties and provide new possibilities for human-technology interaction simulations.

## 2 Materials and methods

The study is divided into three sections: 1) The experimental acquisition of force-displacement curves with the associated surface deformations at six different regions of the abdomen, 2) the processing of the measured data, and 3) the determination of descriptive material parameters by means of inverse FEA.

### 2.1 Study design and participants

A total of ten healthy males (25–37 years) participated in the study. Exclusion criteria were acute abdominal or low back pain, limited range of motion and trunk injuries, nervous system disorders, or skin diseases. All participants were fasting at least 2 h before the start of the study and wore loose pants without a restrictive waistband. Anthropometric characteristics of the participants were taken as shown in Table 1. This included body weight and skinfold thicknesses (Clauser et al., 1988; Norton, 2018). The latter was measured three times at each of seven positions (Norton, 2018) with a calibrated Harpenden Skinfold Caliper. For consistency, all data was recorded by a single examiner. The participants’ body mass index (BMI) was within the normal range (19.5–24.6 kg/m^2^). The Ethics Committee of the Medical Faculty of the Ruhr-University Bochum approved the study (23-7868 08/10/23) and participants provided written informed consent.

**TABLE 1:** Anthropometric characteristics of the participants. Body fat percentage was assessed indirectly using a 4-compartment skinfold-thickness equation (Peterson et al., 2003).

	Mean ± SD	Range	Unit
Age	31.8 ± 3.25	25–37	years
Body mass	74.1 ± 6.41	62–85	kg
Body height	181.4 ± 7.67	168–191	cm
BMI	22.52 ± 1.53	19.5–24.6	kg/m^2^
Chest girth	93.7 ± 8.82	80.9–114.3	cm
Waist girth	81.7 ± 5.64	70.4–87.63	cm
Gluteal girth	99.8 ± 3.93	91.5–106.7	cm
Forearm girth	26.4 ± 1.19	23.8–28.2	mm
Abdominal skinfold	20.9 ± 8.41	7.0–32.0	mm
Suprailiac skinfold^*^	10.7 ± 4.03	4.5–18.0	mm
Iliac crest skinfold	17.1 ± 5.69	7.7–24.5	mm
Front thigh skinfold^*^	14.0 ± 5.47	4.7–21.2	mm
Triceps skinfold^*^	10.1 ± 3.17	4.9–15.0	mm
Subscapular skinfold^*^	12.8 ± 4.16	7.1–19.8	mm
Chest skinfold	9.79 ± 4.86	4.1–18.9	mm
Body fat	19.9 ± 4.49	10.3–24.1	%

The skinfold-thicknesses used for the body fat calculation are marked with ^*^.

### 2.2 Equipment and technical calibration

**FIGURE 1:**
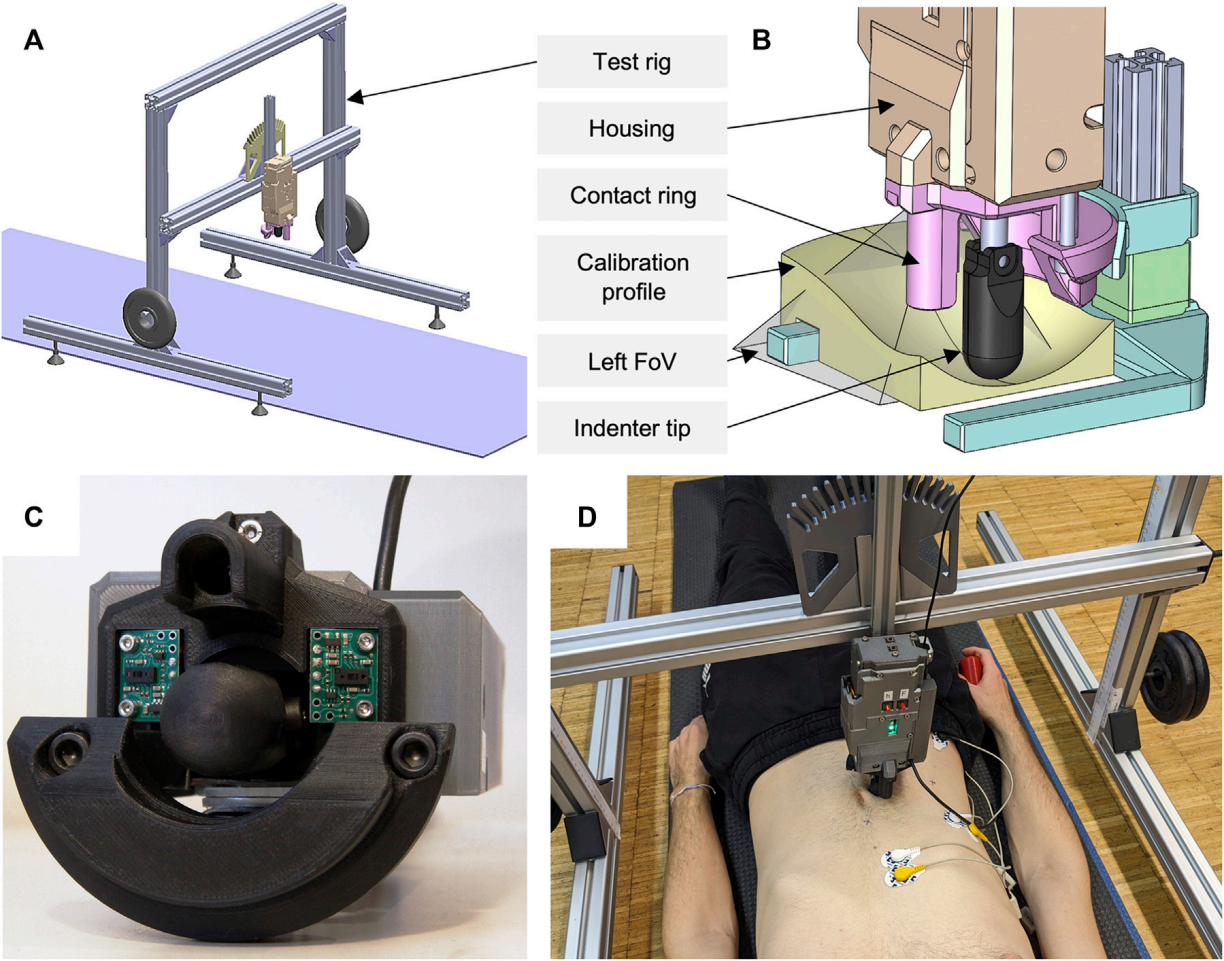
Overview of the experimental setup used. **(A)** Complete test rig with yoga mat, two additional weights, and mounted indenter in vertical position. **(B)** ToF sensor calibration setup with visualization of the left field of view (FoV) and an exemplary calibration profile. **(C)** Close-up of the mechatronic indenter (bottom view). Both ToF sensors, the contact ring with cutouts, and the indenter tip can be seen. **(D)** Participant in the experimental procedure of measurement region R2 with sEMG electrodes applied and the remote control in his right hand.

A custom-built mechatronic tissue indenter with test rig (Figure 1) was used to gather the force-displacement curves and the surface displacements simultaneously. The indenter tip was moved towards the participant through a ring [in analogy to Carter et al. (2001)] mounted on the indenter housing. The ring was not continuous, but had cutouts laterally, allowing for visual measurement of the skin deformation (Figure 1C). To define the zero position for each test and to ensure a perpendicular measurement orientation, this contact ring rested lightly on the skin so that the tissue was just visibly compressed. The plastic indenter tip had the shape of a hemisphere with radius $r_{t}=10\text{ mm}$ . The feed rate was 5 ± 1 mm/s and realized by an electronic micro linear drive. The feed force was measured with a 222.4 N (50 lbf) load cell, mounted to the side of the indenter facing away from the participant, which was subjected to the force applied by the linear drive. Maximum travel and maximum feed force were adjustable. A test rig made of torsion-resistant 40 × 40 mm aluminum profiles was used to mount the mechatronic indenter above the participant (Figure 1A). Both height and alignment of the indenter were adjustable on the test rig for each participant and measurement position. To avoid injury, the feed force was electronically and mechanically limited based on the estimated pressure between skin and indenter tip. The limits were taken from findings for the design of workplaces with collaborating robots (Muttray et al., 2014; Melia et al., 2015) and correspond to the point at which the increasing perception of pressure from an indenter tip turns into a noticeable pain. The lower pain limit for abdominal muscles was 35 N/cm^2^. Because no pressure limits were known for paraspinal tissue, we used a software-controlled force limit of 110 N for all positions. A hardware force limitation was additionally implemented in case the electronic limitation failed. The dead weight of the free-standing test rig was 10% above the software-controlled force limit, so that it lifted off the ground when limits were exceeded. A wired remote control was used to allow participants to start and interrupt a measurement by themselves.

To calibrate our force-displacement test setup with tissue-like material (Wells and Liang, 2011), we used cylinders made of ballistic gelatin (GELITA BALLISTIC 3 gelatin, 255–265 g Bloom). The cylinders, 100 mm in diameter and 80 mm in height, were produced according to the preparation procedure of GELITA: Comprised of heating distilled water and gelatin to a maximum of 55°C ± 5°C, curing the mixture in molds for 24 h, demolding the specimens, and conditioning for 60 h within a moisture-sealing barrier. Conditioning temperatures for our cylinders with 10 wt% gelatin solution were 4°C according to FBI recommendations (Fackler and Malinowski, 1988; Maiden et al., 2015) and 15°C for increased compliance. Since gelatin is very sensitive to shear stress (Wells and Liang, 2011; Valliere et al., 2018), we covered the entire top of the gelatin cylinder with a rigid acrylic plate during calibration. Ultrasonic gel was applied in between. A cylinder with 10 mm radius was used as indenter tip. We compressed the differently tempered cylinders three times each at 5 mm/s up to 200 N using our experimental test setup (Figure 1A) and a materials testing machine (Zwick Z010 with GTM GmbH series K, 10 kN, 2 mV/V). Maximum deformation was 24.6 mm at 15°C. Comparing the measurement accuracy, maximum root mean square errors (RMSE) were 0.389 and 0.433 N, and percentage deviations were below 2.4% and 3.1%, for maximum compression forces of 60 and 120 N.

To optically measure the 3D surface deformations, two 8×8 Multi-Zone ToF sensors (VL53L5CX, STMicroelectronics) were used. These were mounted sagittal symmetrically to the sides of the indenter tip on the housing, each at a 12° angle to the observation plane (Figure 1C). The sensor distances perpendicular to the reference plane (zero position) were 35.1 mm. Each trapezoidal field of view, starting at the tip of the indenter, had a diagonal of 63° and a minimum length of 39.1 mm laterally to the reference plane. This resulted in a minimum observable area of approximately 16.74 cm^2^. When the tissue is deformed starting from its initial state, the measurable area increases. For calibration of the ToF sensors and their data processing, we used 3D printed PLA (polylactic acid) profiles in matt white after ensuring that the optically measured distances did not differ from those of the skin [all participants had a Fitzpatrick skin type (Fitzpatrick, 1988) of I-III]. The seven profiles (including horizontal planar, bevelled at 12° and thus parallel to the sensor plane, convex or concave converging towards the sensor tip) represented continuous surface deformations from 0 to 45 mm (Figure 1B). The processing of the 15 Hz raw sensor data included a transformation of the absolute distance values for the 64 measuring zones into the 3D displacement of the trapezoidally measured surface, a calculation of the means over a sliding window of length 5 across the neighboring elements, and a linear time interpolation to 0.1 s. The comparisons to the fully known geometries resulted in absolute measurement deviations of ±1.6 mm. Maximum deviations occurred in the peripheral measurement zones. Mean RMSE and standard deviation (SD) over all profiles was 0.805 ± 0.45 mm.

### 2.3 Surface electromyography

**FIGURE 2:**
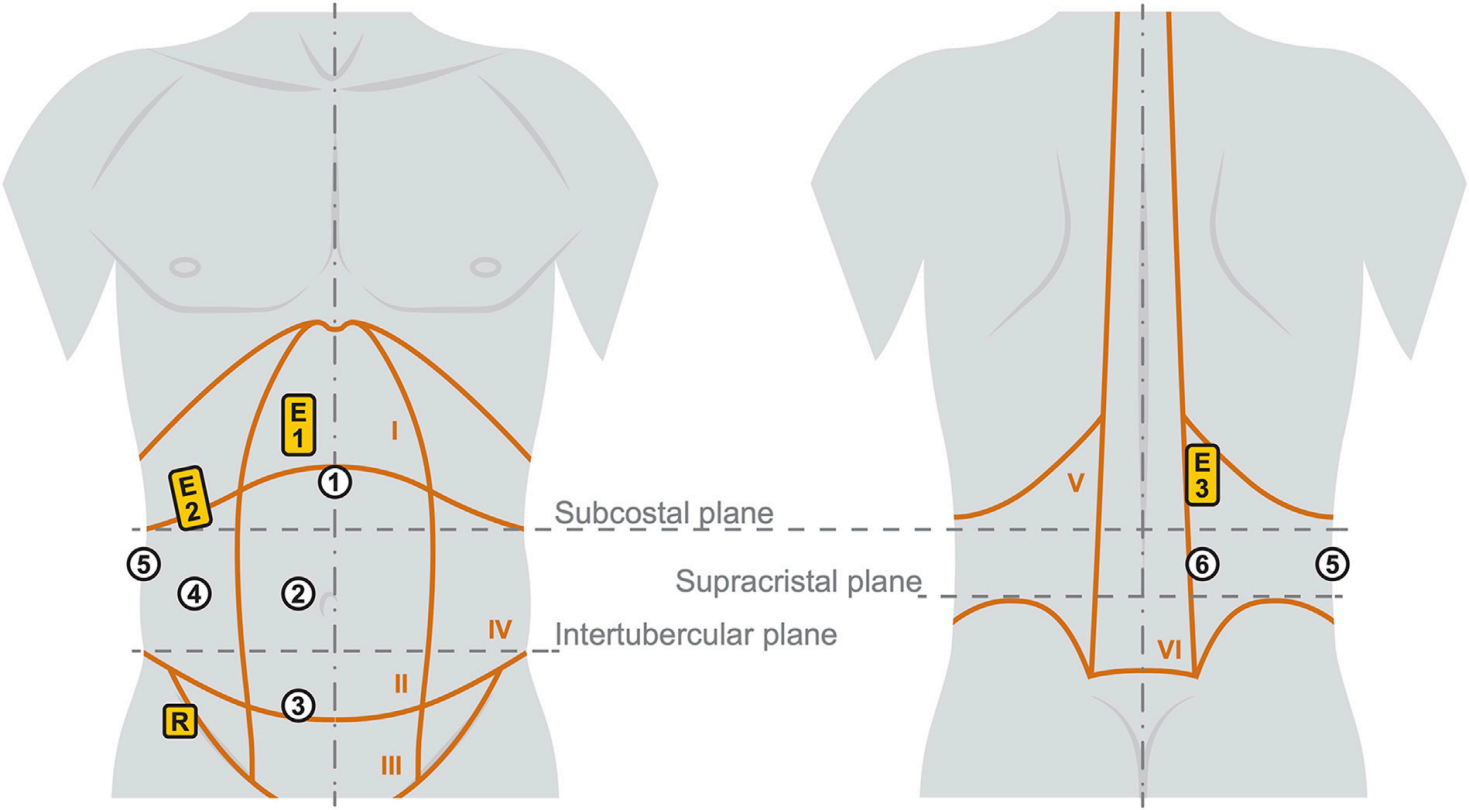
Illustration of the six measurement regions (white circles) with the three sEMG positions (yellow rectangles). The trunk with indicated anatomical characteristics is shown on the left from anterior and on the right from posterior. Details and labels of the Roman numbered body regions I-VI are given in Table 2.

During the indenter measurements, bipolar sEMG activity of the three main trunk muscles was recorded using 42 × 24 mm Ag/AgCl disposable surface electrodes with hydrogel (Kendall H93SG). Following skin preparation, three pairs of electrodes were placed on the right side of the body on the anterior abdominal (E1), the lateral abdominal wall (E2), and the paraspinal musculature (E3) (Criswell, 2011) with a center-to-center distance of 24 mm. To not interfere with the optical measurements of the skin deformations, the placements of E1, E2, and E3 were cranial to the subcostal plane. E1 was centered on the rectus abdominis and E3 was centered on the muscle belly of the erector spinae. E2 was located at the level of the most caudal palpable costa spuria in the transition between the hypochondric and right lumbar region (Figure 2). The reference electrode was placed caudally to the lateral abdominal wall muscles in the region of the anterior superior iliac spine. For sEMG signal acquisition, we used three bipolar preamplifiers of type ToMEMG V1.2 and a Tower of Measurement (DeMeTec GmbH, Langgöns, Germany). sEMG signals were sampled at 1024 Hz, filtered, quantified, visualized, and recorded using custom-built software. To mitigate the influence of electrocardiographic and power line artifacts, a band-pass filter between 45 and 500 Hz and a notch filter at 50 Hz were applied to the raw signals. For quantization, root mean square of the filtered signal was calculated on a sliding window of 200 samples (195.3 ms).

To create a frame of reference for normalization, participants performed maximum voluntary contractions (MVC) in three positions and received the following instructions:

1) MVC in supine position: Crunch with legs bent 90° and abdominal muscles actively tensed after inhaling. Arms at sides of torso, shoulders and head not touching the floor. Gaze is centered on the test bench.
2) MVC in left lateral position: Jackknife with legs and the arm on top extended. The left arm is locked behind the head, the back and neck are kept straight. Legs and head do not touch the floor.
3) MVC in prone position: Superman with arms and legs outstretched. Head, legs, and arms do not touch the ground.

For the sEMG amplitude normalization, the MVCs from the same positions as in the measurements were used in each case. The baseline sEMG activity was recorded when the participants were lying fully relaxed in all three positions before the start of the measurements.

### 2.4 Experimental procedure

Six measurement regions were deduced from the muscular structures and anatomical characteristics of the abdomen (Rohen et al., 2015; Netter, 2017; Schünke et al., 2018; Tayebi et al., 2021) as visualized in Figure 2 and explained in more detail in Table 2. The regions were identified by palpation (Van Sint Jan, 2007) and marked with a water-soluble pen. To eliminate individual and uncontrollable influence of the trunk muscles to stabilize the spine (Hodges et al., 2015), the participants were lying in three positions: supine, lateral, and prone. Four measurements were taken at each region with fully relaxed (R) and controlled activated (A) musculature, leading to a total of 48 measurements per participant. The regions are numbered from 1 to 6 and measurements were carried out in the same order (for instance: indentation with fully relaxed muscles in measurement region 1 is named R1). To maximize comparability, participants were given the following instructions:

• All measurements: Exhale before starting a measurement and hold the breath while the indenter tip moves out.
• R1-R4 (Supine position): Your legs and arms are stretched out and lie flat on the yoga mat. The head lies on the pillow so that all your muscles are fully relaxed.
• R5 (Left lateral position): Your head lies on the pillow and the legs are slightly bent on top of each other on the yoga mat. The upper arm is held in front of the body. The left arm can be placed under the pillow to support the head and relax the lateral muscles.
• R6 (Prone position): The legs are stretched out and lying on the yoga mat. Your head lies sideways on the pillow and the arms are crossed next to the head. All muscles should be relaxed.
• A1-A4 (Supine position): Your legs and head do not touch the mat. The legs form a right angle. The center of your gaze is directed towards the indenter. The arms are at the side of the upper body.
• A5 (Left lateral position): Your right leg and head are not touching the mat. The right leg is fully extended and in line with the head. To stabilize the body, the right arm is held in front of the body and the left leg is slightly bent. The left arm is stretched upwards.
• A6 (Prone position): Legs and head do not touch the mat. Your legs are stretched out. The arms are folded to the side of the head and are just not touching the mat.

**TABLE 2:** Test plan of the unilateral indenter measurements. Assuming abdominal symmetry, the measurements were solely performed on the right side of the body. Each measurement was carried out with fully relaxed (R) and controlled activated (A) muscles at the six regions listed (*cf.* Figure 2).

Region number	Lying position	Body region	Description of the measurement region
1	Supine	Transition between epigastric and (I) umbilical region (II)	Centered on the linea alba, about 1 cm cranial to the subcostal plane
2	Supine	Umbilical region	Centered on the rectus abdominis, at the supracristal plane. In most cases, this was below the second tendinous intersection (fibrous band) and at the level of the umbilicus
3	Supine	Transition between umbilical and hypogastric region (III)	Centered on the rectus abdominis, about 1 cm below the intertubercular plane
4	Supine	Lateral abdominal region (IV)	On a line with the mamilla, at the supracristal plane. Thus, lateral to the rectus abdominis and central on the lateral abdominal wall
5	Left lateral	Lateral abdominal region (V)	Most lateral position of the abdomen, midway between subcostal and supracristal plane
6	Prone	Transition between lateral abdominal and lateral lumbar region (VI)	Centered on the lumbar erector spinae muscle belly, midway between subcostal and supracristal plane. In most cases, this was at the level of vertebra L3

By comparing the monitored sEMG amplitudes to that of other participants, the postures were evaluated before and during each measurement and adjusted by interacting with the participant if necessary. To reduce tilting of the pelvis and chest in coronal plane, a folded towel was placed under the waist.

After the posture instructions, ultrasound gel was applied to the measurement region to minimize friction between skin and indenter tip. The test rig was set up so that the indenter was perpendicular to the body (Figure 1D) and the contact ring was in light contact with the skin. This was checked before each single measurement. To familiarize participants with the measurement procedure, test measurements were performed at region 1 with R and A at least once. If the patient breathed during an indentation, significant changes in muscle activity were observed, or other disturbances were detected, the respective measurement was repeated.

### 2.5 Data processing

**FIGURE 3:**
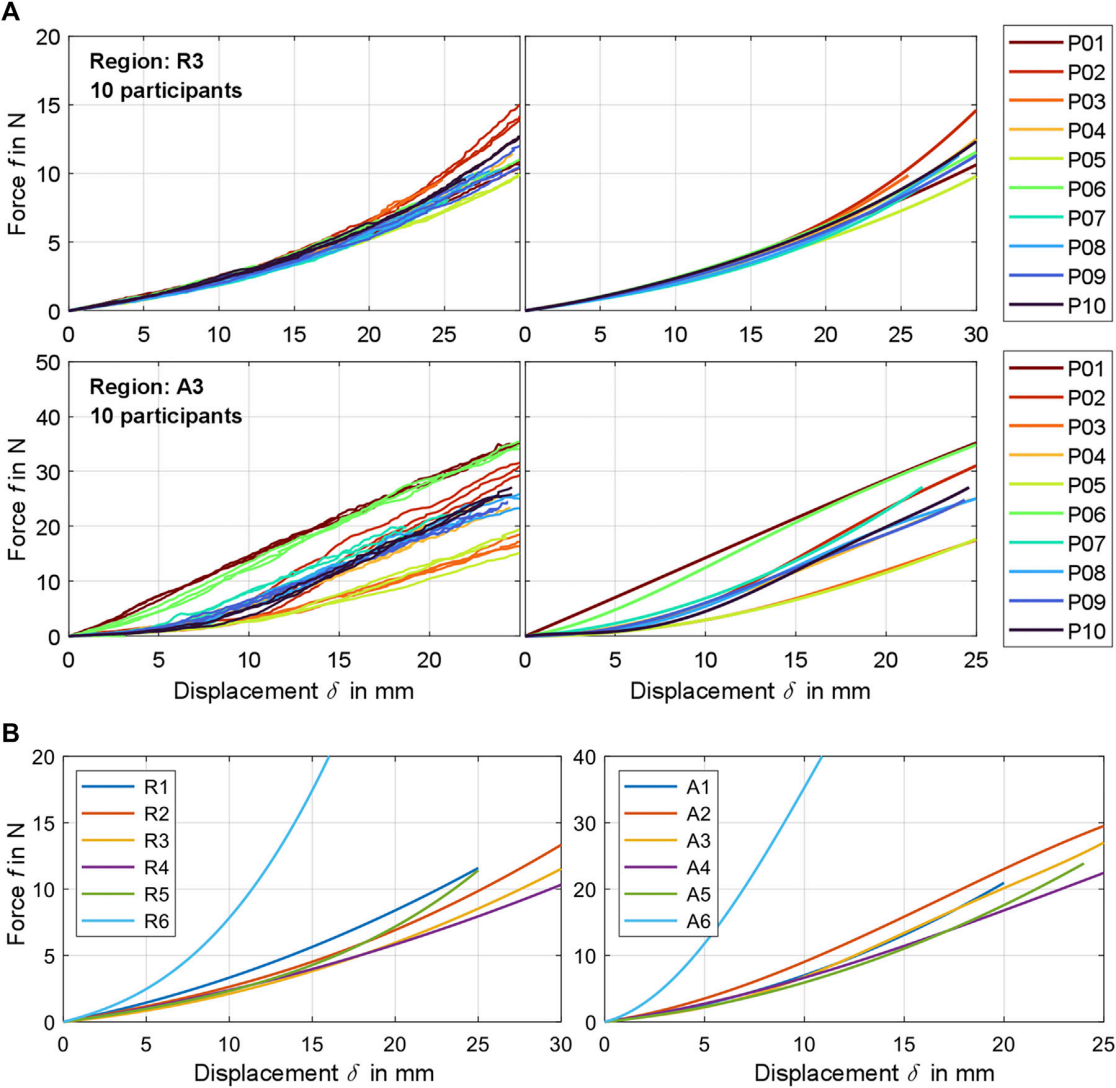
**(A)** Exemplary comparison of experimentally determined raw force-displacement curves (left) and the participant-related curve fits calculated from these (right) for measurement region 3 for fully relaxed (R) and controlled activated (A) musculature. A visualization for all participants and regions can be found in Supplementary Material S3. **(B)** Compilation of all mean curve fits $f\left(\delta \right)$ for the six measurement regions, each for the range $0\leq \delta \leq \delta_{\max}$ .

Data was processed and analyzed using MATLAB R2022b (MathWorks, Inc., Natick, MA). Three measurements were manually selected for each participant at each measurement region. Data recorded before a detectable contact force and after maximum stroke was not considered for further data processing. For the force-displacement curves, the raw data was cleaned of recording-related outliers. Polynomial curve fits $f\left(\delta \right)$ of degree n=1,2,…,5 were used for the uniform and continuous description of the non-linear curves for each participant (Figure 3A) and as a mean for each measurement region (Figure 3B). The polynomials (1) were selected according to two criteria: the least possible degree with minimum RMSE, and validity in the range $0\leq \delta \leq \delta_{\max}$ , based on the available cleaned measurement data.

$$f\left(\delta \right)=f_{1}\delta^{n}+f_{2}\delta^{n-1}+\ldots +f_{n}\delta +f_{n+1}$$ (1)

**FIGURE 4:**
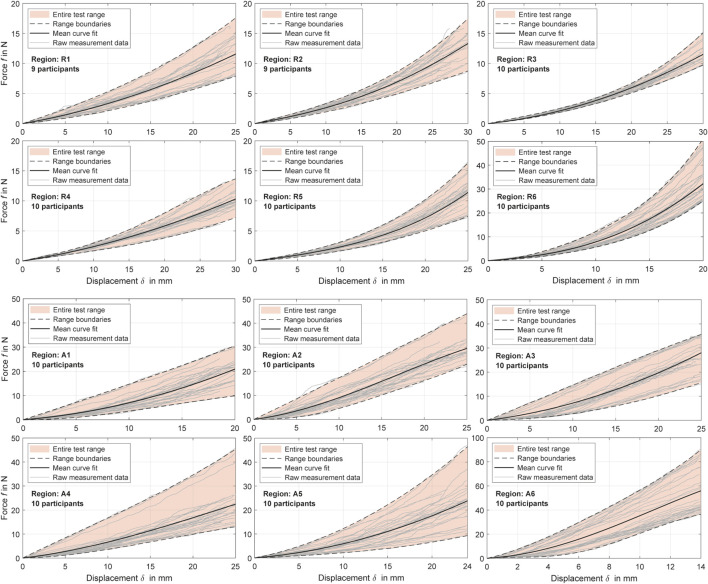
Experimental force-displacement curves for all measurement regions with distinction between relaxed and activated musculature (R and A). The polynomial curve fits (Table 4) are shown alongside the cleaned raw data for all participants and the resulting value ranges.

The coefficients $f_{n}$ are in descending powers, and the length of f is n+1 . The mean force-displacement curves for each measurement region with R or A were based on all the respective raw data sets of the participants. To quantify entire test ranges, these combined data sets were also used to create conforming 2D boundaries, the upper and lower limits of which were each described by a curve fit (Figure 4). Goodness of fits (Sum of Squares Due to Error, R-Squares, and RMSEs) are provided with all coefficients $f_{n}$ in Supplementary Material S1.

**FIGURE 5:**
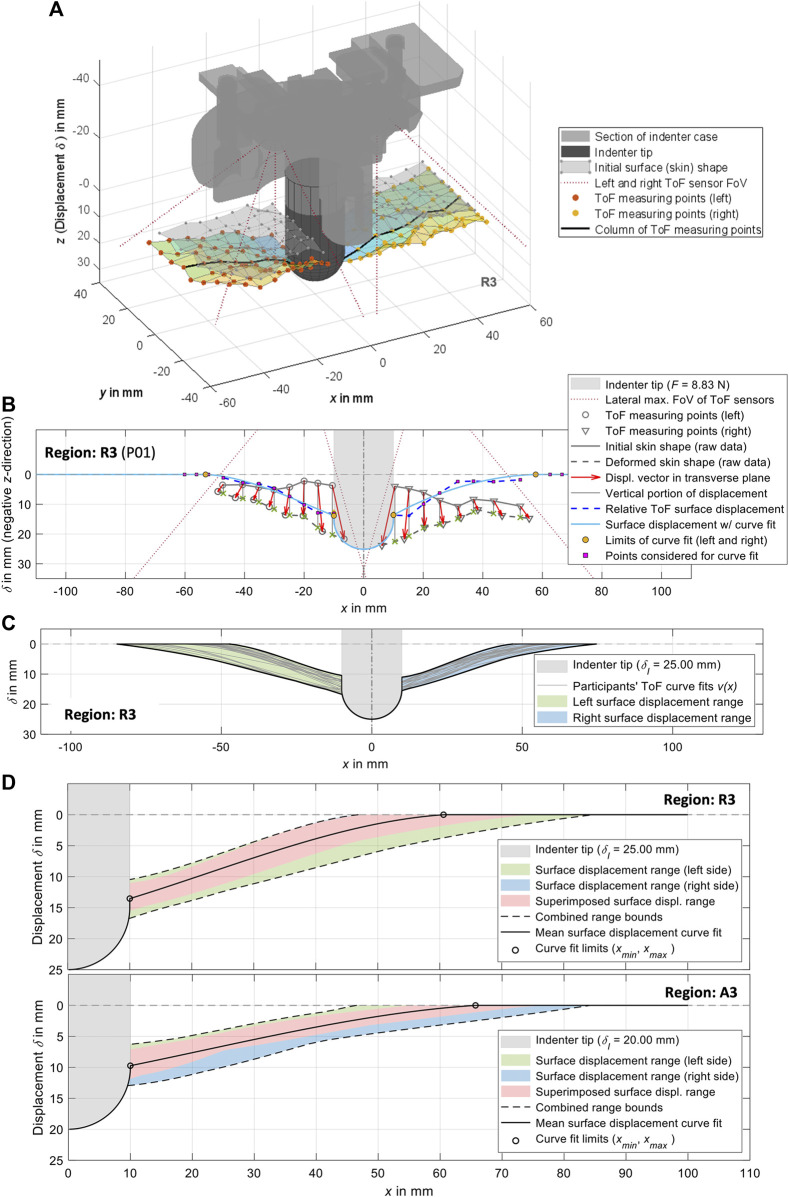
Visualizations of the experimental surface measurements and their processing exemplarily for measurement region 3. **(A)** 3D plot of the ToF measuring points from the left and right side, together with a visualization of the indenter tip and the indenter case. In addition to the initially measured undeformed skin, the deformation resulting from the measuring points is also shown. **(B)** Procedure for generating curve fits from the ToF measuring points in transverse plane (*cf.* Section 2.5). **(C)** Compilation of the left and right curve fits for all participants with visualization of the resulting ranges. **(D)** Complete surface displacements for both sides of all participants for $\delta_{I}$ . The mean surface displacement consists of the outer edge of the indenter tip that is in contact with the skin, the mean curve fit representing the deformation profile of the skin (Table 5), and an idealized undeformed section. The maximum radius of influence of the indentation is specified by $x_{\max}$ .

For the relative skin or surface displacements, we used the measuring points from the fifth row from the anterior of both ToF sensors (Figure 5A), which were synchronized in time with the force-displacement data. These two sets of eight ToF measuring points were located to the left and right of the indenter tip respectively and were used to deduce continuous surface displacement curves: In relation to the reference plane, the vertical components, as visualized in Figure 5B, served as the relative surface displacements. Offsets to the indenter tip were eliminated on the surface side. The first measurable contact force as a result of an incipient deformation was taken as reference. Assuming that measuring points close to the indenter tip are distorted, raw data in the range $-0.9\;r_{t}\leq x\leq 0.9\;r_{t}$ were discarded. The direct connection of the remaining most central measuring points from the left and right side resulted in two intersection points with the indenter tip profile. From these, together with the measuring points and three additional base points on the reference plane, curve fits of third degree were created separately for each side. The base points on the reference plane were introduced because the field of view of the ToF sensors did not always capture the entire displacement. Consequently, the start of the deformation on the reference plane could be unknown. To approximate it, the positions $x_{B}$ of the base points located within an 8 mm interval were shifted in 5 mm steps from the outermost ToF measuring point to $\left|x_{B}\right|\leq r_{C}=r_{t}+100\text{ mm}$ and a fit was calculated in each case. This is subject to the assumption that the surface displacements are continuous and can be described by a third degree polynomial. The selection of a participant-specific fit $v\left(x\right)$ per side and indentation depth $\delta_{I}$ is done by minimizing the cost function $ϴ\left(x_{B}\right)$ in (2).

$$ϴ\left(x_{B}\right)=RMSE_{v\left(x\right)}+Q,$$ (2)

$$Q=\left\{\begin{array}{l} 0.75\left|\frac{x_{xmax}}{r_{C}}\right|\text{ if }v\left(x\leq r_{C}\right)\cap \text{reference plane}\neq \emptyset \\ 10\text{ if }v\left(x\leq r_{C}\right)\cap \text{reference plane}=\emptyset \end{array}\right.$$

$RMSE_{v\left(x\right)}$ is the standard error of the regression of $v\left(x\right)$ and $x_{xmax}$ is the intersection point of $v\left(x\right)$ with the reference plane for a given $x_{B}$ . If no intersection existed, the penalty factor was applied instead.

For mean surface displacement per measurement region $u\left(x\right)$ (Figure 5D), a fit of third degree was calculated over all $v\left(x\right)$ (Figure 5C) of both sides combined (mirrored left side) for one $\delta_{I}$ each. Each of these complete experimental surface deformations is defined for the range $0\text{ mm}\leq x\leq r_{C}$ and includes the profile of the indenter tip in the negative *z*-direction for $x<x_{\min}$ and the reference plane for $x>x_{\max}$ . With n=4 , $u\left(x\right)$ is analogous to (1). As part of the objective function analysis (Section 2.9), the transitions to the indenter tip and the reference plane were automatically smoothed in an *x*-axis section of up to 10 mm for a continuous displacement curve.

### 2.6 Statistical analysis

The experimental force-displacement results were statistically analyzed for T1) differences between the six measurement regions with similar muscle activities (R or A), and T2) differences between muscle activities for the six measurement regions. A *p*-value of <0.05 was considered statistically significant. Statistical tests were conducted for δ=7,14,and 20 mm where available. As a result of these multiple comparisons, Bonferroni correction factors were used. All data sets for all subjects were tested for normal distribution using the one-sample Kolmogorov-Smirnov test. Because the test was rejected for a number of data sets, we used the nonparametric Kruskal–Wallis test for T1), considering each measurement region independent from one another. Post hoc comparisons were made with Tukey’s honestly significant difference procedure (multiple comparison test) for comparisons between regions. Assuming that the respective measurements from R and A are paired, we conducted a non-parametric Friedman test with a Bonferroni factor of 2 ( α=0.025 ) for T2).

### 2.7 Axisymmetric FE model

**FIGURE 6:**
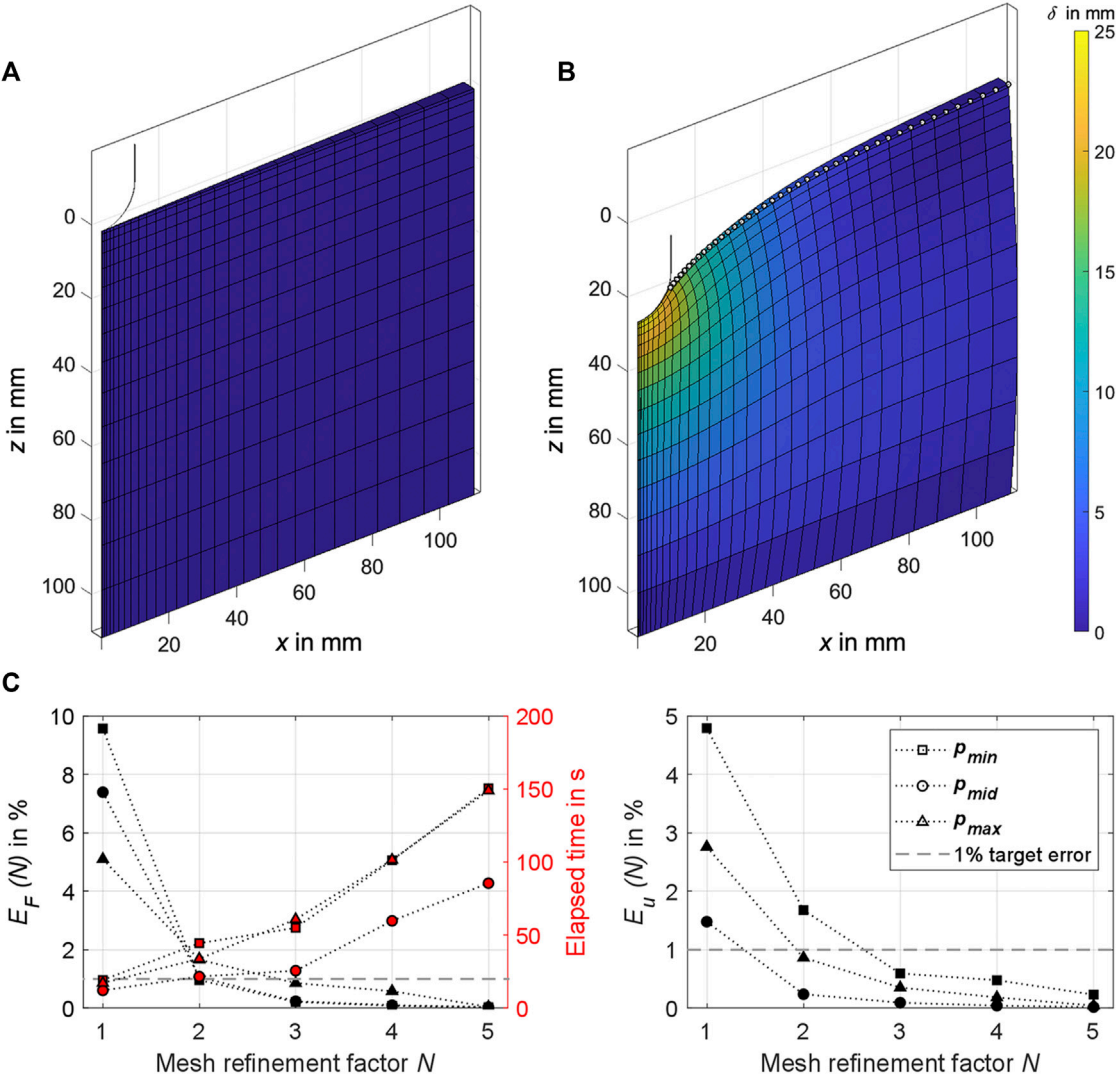
**(A)** The 2° sector of the axisymmetric FE cylinder as used in the analysis. The section of the hemispherical indenter tip in the initial state is also shown in the cylinder’s center. **(B)** Exemplary simulation result for δ=25 mm. The displacements of the finite elements are colored, and the nodes used in the objective function $F_{u}$ [Eq. (8)] are highlighted with white dots. **(C)** Results of the FE mesh convergence analysis for three material sets $p_{\min}=\left(1\text{ kPa},4\right)$ *,* $p_{mid}=\left(45\text{ kPa},30\right)$ , and $p_{\max}=\left(140\text{ kPa},60\right)$ each plotted against the mesh refinement factor *N* used. On the left are the relative errors of the indentation forces $E_{F}$ [Eq. (3)] with the elapsed solution times (plotted in red), and on the right the relative errors of the surface displacements $E_{u}$ [Eq. (4)]. The convergence target error for $E_{F}$ and $E_{u}$ is shown on both sides.

For the inverse FEA of an axisymmetric indentation test, we utilized the framework *indentify* (https://github.com/SolavLab/indentify, v1.0.1) developed and made available by Oddes and Solav (2023) and adapted it to our needs. For pre- and postprocessing of the simulations, running in the FE solver FEBio v4.3 (Maas et al., 2012), we used MATLAB R2022b with the open-source toolbox GIBBON (Moerman, 2018). The indentation model comprises an FE cylinder with radius and height $r_{C}$ and Ogden material model (*cf.* Section 2.8). No differentiations were made between the different layered abdominal tissue components. Skin, fat, muscles, and all subsequent structures were lumped and modeled as a homogeneous material. The indenter tip was modelled rigid with the same geometry as in our experiments and was displaced vertically downwards to the respective indentation depths $\delta_{I}$ . In the tangential section, the 90° circular sector of the indenter tip including a 20 mm cylinder section (Figure 6B) had 60 triangular shell elements. Four equally spaced $\delta_{I}$ were simulated for each region (Table 2) to match with the experimental data. Maximum displacement was $\delta_{\max}=\delta_{I=4}=25$ mm for R2-R5. Friction between indenter tip and cylinder surface was omitted with a friction coefficient of zero using FEBio’s *sliding-elastic* contact formulation. To reduce simulation time, only a cylinder sector of 2° (Figure 6A) was solved with the meshing characteristics and boundary conditions described in detail by Oddes and Solav (2023).

An FE mesh convergence analysis was conducted to check the numerical simulation accuracy for different mesh densities. The mesh density bias was 1.1 towards the center and 0.8 towards the top of the FE cylinder. No larger mesh density bias could be set to ensure model stability until $\delta_{\max}$ and mesh refinement factors *N* < 7. A fine mesh with a mesh refinement factor of $\tilde{N}=6$ served as reference for data comparison at δ=20 mm. Target was a convergence below 1% of the relative errors for the indentation force (3) and the surface displacement (4).

$$E_{F}\left(N\right)=\frac{\left|F\right.\left(N\right)-F\left.\left(\tilde{N}\right)\right|}{\left|F\right.\left.\left(\tilde{N}\right)\right|}∙100\%$$ (3)

$$E_{u}\left(N\right)=\sum_{i=1}^{N_{n}\left(N\right)}\frac{\left|u^{i}\left(N\right)-u^{i}\left(\tilde{N}\right)\right|}{\left|u^{i}\left(\tilde{N}\right)\right|}∙100\%$$ (4)

$F\left(N\right)$ is the maximum indentation force and the vector $u^{i}\left(N\right)$ denotes the final displacement of the $i^{th}$ node on the upper outer edge of the FE cylinder segment (*cf.* Figure 6B) using a mesh refinement factor *N*. $N_{n}\left(N\right)$ is the number of the nodes used. To take into account varying material behaviors, three material sets covering the entire parameter space were analyzed. As a result of the mesh convergence analysis (compare with Section 3.3), we used a mesh refinement factor of *N* = 3 and quadratic elements (hex20 and penta15) in the objective function analysis (Section 2.9). This resulted in 30 elements in radial, 15 elements in axial, and one element in tangential direction.

### 2.8 Constitutive model

Consistent with previous mechanical descriptions of human soft tissue (Holzapfel, 2000; Erdemir et al., 2006; Flynn et al., 2011; Halloran and Erdemir, 2011; Maas et al., 2012; Moerman et al., 2017; Calvo-Gallego et al., 2018; Wang et al., 2019; Marinopoulos et al., 2020; Alawneh et al., 2022; Lohr et al., 2022), we used a hyperelastic, nearly-incompressible Ogden material model (Simo and Taylor, 1991) to describe the nonlinear force-displacement and surface deformation behaviors gathered in this study. With a set of material parameters $p=\left(p_{1},p_{2}\right)=\left(c,m\right)$ and the bulk-like modulus κ , the uncoupled first-order Ogden strain energy density function Ψ integrated in FEBio is defined in (5),

$$\Psi \left(\lambda_{i}\right)=\frac{c}{m^{2}}\;\sum_{i=1}^{3}\left({\tilde{\lambda }}_{i}^{m}-1\right)+\frac{\kappa }{2}{\left(\ln\;J\right)}^{2}$$ (5)

where $\lambda_{i}$ is the $i^{th}$ deviatoric principal stretch and *J* represents the volume ratio. For a nearly isochoric deformation, $\kappa =\frac{c}{2}\cdot 10^{3}$ was selected (Moerman et al., 2017; Oddes and Solav, 2023).

### 2.9 Objective function analysis

Based on the objective function analysis with synthetic reference values of Oddes and Solav (2023), we defined the combined objective function $F_{fu}\left(p;\delta \right)$ as the modulated sum of the overall normalized errors between the indentation forces $F_{f}\left(p;\delta \right)$ and the associated overall normalized relative errors between the surface displacements $F_{u}\left(p;\delta \right)$ in Eq. (6).

$$F_{fu}\left(p;\delta \right)={\eta \;F}_{f}\left(p;\delta \right)+{\left(1-\eta \right)\;F}_{u}\left(p;\delta \right),\;\eta \;\epsilon \;\left[0,1\right]$$ (6)

The modulation factor η enables a convex combination of both residual errors. This means that η=0 or η=1 corresponds to a sole evaluation of the superficial displacements or the indentation reaction forces. The residual errors between the indentation forces at an indentation depth δ are defined in (7).

$$F_{f}\left(p;\delta \right)=\frac{{\left|f\left(p;\delta \right)-f\left(\delta \right)\right|}^{2}}{{\left|f\left(\delta \right)\right|}^{2}}$$ (7)

$f\left(\delta \right)$ is the experimental mean and $f\left(p;\delta \right)$ is the simulated indentation reaction force in negative *z*-direction, respectively. $F_{u}\left(p;\delta \right)$ quantifies the combined surface displacement deviations in (8).

$$F_{u}\left(p;\delta \right)=\sum_{i=1}^{{\bar{N}}_{n}}w_{n,i}\cdot \frac{{\left|u_{\left(i\right)}\left(p;\delta \right)-u_{\left(i\right)}\left(\delta \right)\right|}^{2}}{{\left|u_{\left(i\right)}\left(\delta \right)\right|}^{2}}$$ (8)

$u_{\left(i\right)}\left(p;\delta \right)$ and $u_{\left(i\right)}\left(\delta \right)$ are the simulation and the mean reference surface displacements during an indentation depth δ of the *i* ^th^-node in negative *z*-direction. No displacements in directions other than the *z*-direction could be tracked using our ToF measurement setup. After interpolating the mean reference displacements (Table 5) to all nodes of the simulation model, only the nodes ${\bar{N}}_{n}$ that fulfill $x_{\min}\leq x_{n}\leq x_{Trim}$ were considered. $x_{n}$ is the node position in *x*-direction and $x_{\min}$ represents the outer edge of the indenter tip. $x_{Trim}$ in turn results from the reference displacements $\delta_{Trim}=u\left(x_{Trim}\right)$ , which must at least be given so that a reasonable evaluation of $F_{u}\left(p;\delta \right)$ is possible. Due to the processing of the measurement data (Section 2.5), $u\left({x\geq x}_{\max}\right)=0$ applies to all mean displacements. For the calculation of $F_{u}\left(p;\delta \right)$ , this leads to an underrepresentation of displacement errors near the indenter tip due to $\underset{x\to x_{\max}}{\lim\;}u\left(x\right)=0$ , despite an inversely proportional weighting to their initial radial coordinates $w_{n}$ .

$$w_{n}=\frac{{\left(\frac{1}{r_{1}},\frac{1}{r_{2}},\ldots ,\frac{1}{r_{{\bar{N}}_{n}}}\right)}^{T}}{\left\|{\left(\frac{1}{r_{1}},\frac{1}{r_{2}},\ldots ,\frac{1}{r_{{\bar{N}}_{n}}}\right)}^{T}\right\|}$$ (9)

The nodes visualized in Figure 6B (vertex and edge nodes in the range $x_{\min}{\leq r}_{N_{n}}\leq r_{C}$ ) on the outer edge of the cylinder section were represented by their coordinates $r_{1},r_{2},\ldots ,r_{N_{n}}$ . The number of all surface nodes used in each case was ${\bar{N}}_{n}\leq N_{n}∊N$ .

To analyze the objective functions combined over all indentation depths, $\delta_{I}$ (10) was specified for the resulting parameter space, where p is an element of the discrete parameter space P=C×M .

$$F_{fu}^{tot}\left(p\right)=\frac{1}{4}\sum_{I=1}^{4}F_{fu}\left(p;\delta_{I}\right),\text{ for}I=1,2,3,4$$ (10)

Consequently, the minimum of $F_{fu}^{tot}\left(p\right)$ constitutes the resulting material parameter $p_{res}$ for the FE model, which approximately results in the smallest error to the experimental data (best fit) over all indentation depths and the selected η . The 2D grid of P is based on ***C*** and ***M***, which comprise the evenly spaced Ogden material parameters *c* and *m*: C=1:1:60 kPa , and M=4:1:125 .

We evaluated a trim factor $\delta_{Trim}$ in the range 0.1−1.2 mm best suitable for evaluating the objective function for each measurement region (Table 2) using six parameters: 1) The SD of distances between $F_{fu}^{tot}\left(p\right)$ and all $F_{fu}\left(p;\delta_{I}\right)$ , 2) the proportion of $F_{fu}^{tot}\left(p\right)>1.5$ in P , 3) the circularity measurement for $F_{fu}^{tot}\left(p\right)\leq 1.5$ in P , 4) the relative change of mean $F_{f}\left(p;\delta \right)$ to $F_{u}\left(p;\delta \right)$ over all $\delta_{I}$ , and 5) and 6) the change in the resulting relative material parameters *c* and *m*. Their results were analyzed for the entire parameter space and are visualized as an example in Supplementary Material S2 over the range of $\delta_{Trim}$ . To determine the shape parameters 1–3, the parameter space was nondimensionalized using the respective $p_{\max}=\left(c_{\max},m_{\max}\right)$ . For parameter 4, the minimum difference to 0.5 (equal weighting), assuming the lowest possible trim factor, was aimed for. Convergence was the aim for all other parameters. For 1, 2, and 3 with <2% and for 5, and 6 <1%. The rounded mean value of all six individual target trim factors resulted in the $\delta_{Trim}$ per region used in the objective function analysis.

## 3 Results

All participants were students or academic employees. No complications occurred during the measurement sessions, there were no interruptions, and a complete set of data was recorded in each case. The results of the sEMG measurements are summarized in Table 3. Because the MVCs primarily targeted the muscle regions over which direct measurements were conducted, the most relevant values are highlighted. With regard to the MVC in the supine position, the anterior relative muscle activities at E1 amounted to an average of 3.9% during R1-R3, and 33.2% during A1-A3 (see Figure 2 for measurement regions). The anterolateral measurement region 4 was located lateral to the rectus abdominus. The subjects were in supine position, which is why the MVC in this position was used. Because this and the active posture took place within the sagittal plane, the relative activity at E2 was higher by a factor of 6.4 and 1.5 for R and A respectively compared to E1. For the lateral MVC, the absolute muscle activity at E2 was 216.5% higher, which explains the increased relative activity at E2 during R4. In general, however, the measurement data also confirmed the experimental assessment that the anterolateral muscles measured with E2 were less well activated selectively by the participants. Except during the measurements in region 4, the relative muscle activities in the relevant regions for R were 5.3% ± 2.04% and for A 34.3% ± 7.03%. During measurements R1 and R2 only one participant was unable to fully relax his abdominal muscles (E1 ≥ 10.4%, E2 ≥ 42.1%), as a result of which the forces for δ>5 mm were up to 90% higher than the mean. With the aim of determining physiological standard values, these data sets were excluded from all subsequent evaluations.

**TABLE 3:** Surface EMG amplitude of the participants. Electrode positions E1, E2, and E3 are displayed in Figure 2 and data is given as mean with standard deviation (mean ± SD). The most relevant values for the evaluations are printed in bold (activity of the muscle group on which the indentation was conducted).

	Body position	E1	E2	E3
Baseline activity in µV	Supine	**6.8 ± 1.04**	6.9 ± 2.30	5.0 ± 0.31
	Lateral	8.3 ± 5.09	**6.4 ± 1.42**	4.8 ± 0.41
	Prone	5.2 ± 0.42	5.0 ± 0.34	**6.1 ± 1.73**
MVC in µV	Supine	**235.4 ± 168.95**	61.7 ± 39.46	40.2 ± 35.09
	Lateral	72.7 ± 75.25	**133.6 ± 101.62**	50.5 ± 31.79
	Prone	16.1 ± 10.19	33.3 ± 18.56	**105.2 ± 45.32**
	**Measurement region**			
Muscle activity in %	R1	**3.9 ± 2.4**	14.1 ± 9.3	36.4 ± 30.8
	R2	**4.1 ± 2.1**	18.9 ± 16.5	36.1 ± 30.2
	R3	**3.7 ± 2.3**	14.5 ± 11.5	38.8 ± 33.3
	R4	4.0 ± 2.4	**24.8 ± 23.8**	36.2 ± 30.7
	R5	33.6 ± 41.0	**8.3 ± 5.7**	14.9 ± 10.5
	R6	42.6 ± 18.4	20.3 ± 10.3	**6.6 ± 2.8**
	A1	**30.2 ± 9.4**	40.7 ± 25.2	35.3 ± 32.3
	A2	**32.3 ± 6.6**	41.8 ± 22.7	34.6 ± 28.6
	A3	**37.2 ± 7.4**	51.6 ± 29.8	35.4 ± 29.3
	A4	34.4 ± 5.0	**51.7 ± 30.8**	38.6 ± 37.6
	A5	44.1 ± 39.8	**26.8 ± 13.2**	42.8 ± 33.3
	A6	61.6 ± 22.6	33.3 ± 17.4	**44.9 ± 5.5**

### 3.1 Force-displacement curves

The processed force-displacement curves for the measured regions with distinction between R (fully relaxed musculature) and A (controlled activated musculature) are visualized in Figure 4 and the mean curve fits $f\left(\delta \right)$ are listed in Table 4. As usual for mechanically loaded layered biological soft tissue (Zhang et al., 1997; Huang and Zheng, 2015), non-linear courses with a toe region with very small stiffnesses at low deformation, followed by tissue stiffening with increasing indentation depths, can be observed for the relaxed curves. Transitions between the two sections were on average 12.1 ± 2.5 mm for R and 4.3 ± 1.6 mm for A. The measurements for A were less compliant, and the curves were almost linear after the toe region. The activation of the muscles under the skin and subcutaneous tissue after uniaxial compression therefore resulted in a stiffer overall system that behaved less like usual soft tissue. In comparison, participant 6 (P06) had the lowest body fat percentage at 10.3% (*cf.* Table 1) and a pronounced lateral abdominal musculature due to his athletic background. The measured force at A4 was therefore more than twice as high as the average. For A1, A4, and A5, P06 forms the upper range limits (*cf.* Supplementary Material S3) but shows no deviations from the average when his muscles were relaxed. It could also be seen that the lack of subcutaneous fat <13.6% led to an approximate linearization of the overall anterolateral force-displacement curves in P01 and P06. P02 set the upper limit for A2. He had pronounced trunk muscles to prevent back pain and an average body fat percentage of 17.2%. After a toe region of less than 1.3 mm, the force-displacement curves of P02 were almost linear. The force ranges increased with greater displacements and were 14.6 ± 11.57 N at $\delta_{\max}^{*}$ .

**TABLE 4:** Coefficients of the experimental mean force-displacement curves (Figure 4). Each polynomial curve fit $f\left(\delta \right)$ describing this is defined for $0\leq \delta \leq \delta_{\max}$ with $f_{\max}=f\left(\delta_{\max}\right)$ . The complete data set after curve fitting for the force-displacement measurements can be found in Supplementary Material S1.

Measurement region	$\delta_{max}$ in mm	*f*_*max*_ in N	Degree (*n*-1)	Polynomial curve fit $f\left(\delta \right)$						
				Coefficients $f_{n}$						RMSE in N/mm
				*f*_*1*_	*f*_*2*_	*f*_*3*_	*f*_*4*_	*f*_*5*_	*f*_*6*_	
R1	25	11.85	2	8.684e-03	2.462e-01	0	-	-	-	1.256
R2	30	13.33	4	−2.937e-06	2.590e-04	2.479e-03	2.163e-01	0	-	0.963
R3	30	11.52	2	8.604e-03	1.260e-01	0	-	-	-	0.512
R4	30	10.33	2	5.279e-03	1.859e-01	0	-	-	-	0.660
R5	25	11.41	4	5.359e-06	1.710e-04	3.677e-03	1.740e-01	0	-	1.025
R6	20	32.25	4	−2.765e-05	2.665e-03	2.231e-02	3.214e-01	0	-	2.639
A1	20	20.94	2	3.456e-02	3.556e-01	0	-	-	-	2.913
A2	25	29.51	5	−1.119e-05	−6.415e-04	4.969e-02	5.176e-02	4.619e-01	0	3.320
A3	25	27.01	5	9.067e-06	−5.547e-04	1.047e-02	−2.882e-02	3.835e-01	0	3.922
A4	25	22.44	3	−3.641e-04	2.890e-02	4.072e-01	0	-	-	4.682
A5	24	23.86	2	2.864e-02	3.068e-01	0	-	-	-	4.037
A6	14	55.98	4	−3.118e-04	−3.885e-03	3.465e-01	7.624e-01	0	-	10.959

Overall, measurement regions 2, 3, 4, and 6 showed large linear sections with muscles activated. In the center of the linea alba and laterally (regions 1 and 5) the curve curses remained predominantly non-linear. Mean gradients of the linear sections for regions 1 to 6 with activated musculature could be approximated with 1.42, 1.39, 1.43, 1.08, 1.36, and 5.22 N/mm. For better comparability, we subdivided the curves with relaxed muscles at $0.45\cdot \delta_{\max}$ and specified a stiffness value for each segment. From region 1 to 6, these were 0.34, 0.29, 0.24, 0.26, 0.25, and 0.72 N/mm for the first segment. For the second segment, linearly approximated stiffnesses were 0.56, 0.56, 0.49, 0.41, 0.62, and 2.31 N/mm. The subjects with a body fat >21% represented the lower anterolateral limits of the ranges. For relaxed musculature, no participant represented a systematic upward or downward outlier. The lowest variance between the subject data was observed for R3 and the highest for A5. The resulting RMSEs for the mean curve fits *f* were 1.18 ± 0.765 N/mm and 4.97 ± 2.996 N/mm for all regions with R and A. Consequently, interindividual differences have increased due to the activation of the trunk muscles.

The Kolmogorov-Smirnov tests (T1) were statistically significant ( p<0.001 ) for the indentation depths considered across all participants. This means that neither the six measurement regions for relaxed nor for active musculature originate from the same distribution. Analysis of variances of the various measurement regions also showed that the results for R6 differed significantly ( p<corrected α ) from R3, R4, and R5 as well as R3 from R1. Limited to δ=7 mm, this also applied for R6 to R2. For an activated musculature, only region 6 did not differ significantly from region 2 (*cf.* Figure 3B). Highly significant differences were also found in the Friedman test (T2): Depending on a relaxed or activated musculature, the group means of measurement regions 1 to 6 differ from each other ( p<0.001 ).

### 3.2 Surface displacements

The experimental mean surface displacements for all measurement regions at $\delta_{I}$ are listed in Table 5 and are visualized for region 3 in Figure 5D. All other regions and indentation depths are visualized in Supplementary Material S4. Each displacement curve was calculated using the individual curves of all participants from both body sides. As can be seen in Figure 5, the left and right sides of the measurements differ. The mean deformation curve therefore represents an approximation for the respective region. The weakest symmetry is found in regions 5 and 6 (see colored indication of the sides). Fits of third degree were suitable for describing the measured displacement in all cases. In individual cases, lower or higher degrees led to abrupt transitions, no intersection with the reference plane, or oscillations. A qualitative comparison of relaxed and activated musculature showed that curve shapes for R had greater slope, $x_{\min}$ were smaller (closer to the center of the indenter tip), and $x_{\max}$ were therefore also relatively smaller. In addition, the displacement curves for regions 3, 4, and 5 were concave and for 1, 2, and 6 they were more linear.

**TABLE 5:** Experimental mean surface displacements (Figure 5) for all measurement regions with mean indentation forces $f^{*}=f\left(\delta_{I}\right)$ (Table 4) at the discrete indentation depths $\delta_{I}$ as reference parameters for the inverse FE analysis. Each polynomial curve fit *u(x)* is defined for $x_{\min}\leq x\leq x_{\max}$ (Figure 5D) for an inverted *z*-axis. $x_{\min}$ and $x_{\max}$ are the intersection points with the indenter tip and the reference plane (*δ* = 0), respectively. A visualization of all displacements can be found in Supplementary Material S4.

Measurement region	$\delta_{I}$ for I = 1, 2, 3, 4 in mm	$f^{*}$ in N	Polynomial curve fit *u(x)*					
			Range limits in mm		Coefficients *u*_*n*_			
			$x_{min}$	$x_{max}$	*u*_*1*_	*u*_*2*_	*u*_*3*_	*u*_*4*_
R1	5	1.45	6.089	42.859	−1.065e-04	6.927e-03	−3.190e-02	−2.971
	10	3.33	7.831	53.080	−4.762e-05	3.425e-03	8.567e-02	−7.078
	15	5.65	8.837	62.945	−4.873e-05	4.178e-03	1.030e-01	−10.89
	20	8.40	9.671	66.729	−6.188e-05	5.926e-03	8.836e-02	−13.9
R2	6.25	1.51	7.342	37.005	−1.993e-04	1.256e-02	−1.166e-01	−2.782
	12.5	5.53	8.943	45.946	−1.246e-04	8.224e-03	6.108e-02	−8.087
	18.75	6.27	9.756	57.187	−5.454e-05	3.227e-03	2.287e-01	−13.43
	25	9.86	10.000	65.213	−5.666e-05	4.188e-03	2.368e-01	−17.54
R3	6.25	1.12	7.566	35.484	−2.180e-04	1.351e-02	−1.365e-01	−2.433
	12.5	2.92	9.293	42.561	−1.745e-04	1.119e-02	5.990e-03	−7.075
	18.75	5.39	9.942	52.568	−6.188e-05	3.089e-03	2.467e-01	−12.52
	25	8.53	10.000	60.609	−6.175e-05	4.191e-03	2.418e-01	−16.31
R4	6.25	1.37	7.348	36.761	−1.688e-04	1.022e-02	−6.487e-02	−3.037
	12.5	3.15	9.026	49.639	−3.117e-05	3.522e-04	2.401e-01	−8.972
	18.75	5.34	9.831	61.055	−1.742e-05	−5.439e-04	3.221e-01	−13.67
	25	7.94	10.000	67.845	−1.813e-05	−4.167e-04	3.827e-01	−18.39
R5	6.25	1.28	7.331	39.196	−1.194e-04	7.065e-03	−8.779e-03	−3.318
	12.5	3.21	8.955	43.200	−1.068e-04	5.409e-03	1.698e-01	−8.818
	18.75	6.34	9.697	52.244	−4.996e-05	1.204e-03	3.545e-01	−14.68
	25	11.41	9.990	61.790	−5.988e-05	3.226e-03	3.379e-01	−19.07
R6	4	1.81	6.811	43.314	6.69e-07	−4.281e-04	5.611e-02	−1.682
	8	5.25	8.518	45.484	−4.42e-05	2.257e-03	7.747e-02	−4.033
	12	11.10	9.272	51.617	2.646e-07	−2.121e-03	2.639e-01	−8.007
	16	19.96	9.865	57.954	8.678e-06	−3.241e-03	3.435e-01	−10.71
A1	4	1.98	6.425	45.758	−7.288e-06	1.786e-04	5.058e-02	−1.99
	8	5.06	8.082	45.302	−6.09e-05	3.765e-03	5.473e-02	−4.543
	12	9.24	8.921	49.949	−8.825e-05	6.103e-03	6.618e-02	−3.34
	16	14.54	9.463	55.936	−5.966e-05	4.297e-03	1.412e-01	−10.9
A2	5	3.52	7.095	46.183	−1.724e-05	5.926e-04	6.401e-02	−2.522
	10	9.04	8.513	50.939	−3.94e-05	2.501e-03	9.710e-02	−6.23
	15	15.84	9.315	57.370	−4.751e-05	3.453e-03	1.354e-01	−10.16
	20	23.02	9.878	65.679	−3.791e-05	2.684e-03	1.961e-01	−13.72
A3	5	2.19	7.553	39.789	1.582e-06	−3.823e-04	6.318e-02	−2.008
	10	6.77	9.309	47.862	−2.645e-05	1.298e-03	9.510e-02	−4.625
	15	13.42	9.795	56.835	−2.952e-05	1.614e-03	1.561e-01	−8.663
	20	20.12	10.000	65.710	−2.59e-05	1.704e-03	1.772e-01	−11.65
A4	5	2.74	7.252	49.359	5.653e-06	−9.252e-04	8.103e-02	−2.425
	10	6.66	8.761	49.564	−8.992e-06	−9.275e-04	1.988e-01	−6.479
	15	11.44	9.584	57.680	−7.473e-06	−1.211e-03	2.744e-01	−10.36
	20	16.80	9.981	63.514	−1.605e-05	−1.236e-04	2.837e-01	−13.41
A5	5	2.25	6.772	47.663	7.959e-06	−1.075e-03	9.514e-02	−2.954
	10	5.93	8.856	53.956	2.45e-05	−3.237e-03	2.213e-01	−6.363
	15	11.04	9.616	54.403	1.31e-05	−3.276e-03	3.356e-01	−10.67
	20	17.59	9.969	59.772	7.236e-06	−3.051e-03	3.979e-01	−14.43
A6	3.25	5.97	6.440	52.595	1.001e-05	−9.623e-04	4.477e-02	−1.149
	6.5	17.97	8.652	54.386	3.466e-05	−3.406e-03	1.263e-01	−2.373
	9.75	33.95	9.422	61.089	3.14e-05	−3.384e-03	1.605e-01	−4.337
	13	51.03	9.817	67.835	3.628e-05	−4.454e-03	2.358e-01	−6.824

### 3.3 FE mesh convergence analysis

We ran all simulations on a PC with Windows 11 Pro, Intel Core i7-10700K 3.80 GHz CPU, and 32 GB RAM. The results for the FE mesh convergence analysis are shown in Figure 6C. Given a short calculation time of ≤60 s, the errors for $E_{F}\left(N\right)$ and $E_{u}\left(N\right)$ are convergent and <1% with N=3 . Lower *N*, however, provided a barely relevant time advantage, but increased the error for the surface displacement almost threefold, especially with more compliant material.

### 3.4 Hyperelastic abdominal material parameters

**FIGURE 7:**
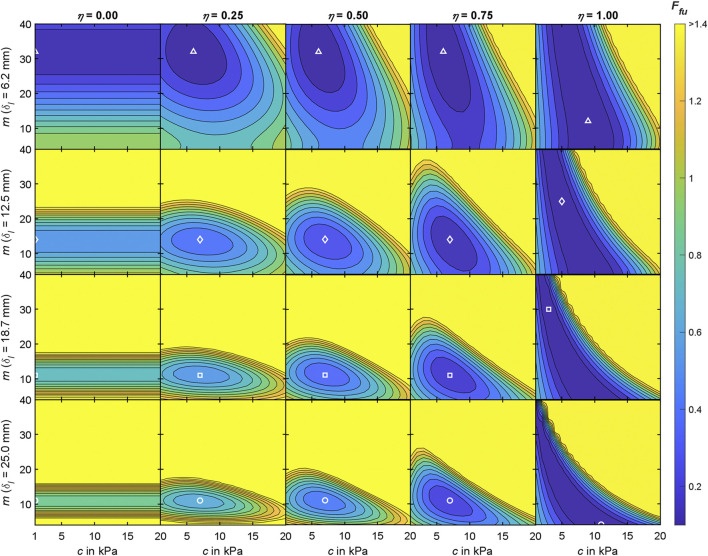
Interim results of the objective function analysis for the Ogden material model at measurement region 3 with C=1:1:20 kPa, and M=4:1:40 . The 20 contour plots depict the resulting dimensionalized shapes of $F_{fu}\left(p;\delta_{I}\right)$ [*cf.* Eq. (6)] for five modulation factors η and the four indentation depth steps $\delta_{I}$ (Table 5). For recognizable scaling of the contours, values > 1.4 are discarded and shown in light yellow. White, unfilled markers indicate parameter sets $p_{I}$ as locations of min $F_{fu}\left(p;\delta_{I}\right)$ for $\delta_{I}$ from small to large: Triangle, rhombus, square, and circle. For η=0.5 , min $F_{fu}\left(p;\delta_{I}\right)$ are 0.06, 0.27, 0.37, and 0.42 and $p_{I}$ are (6 kPa, 32), (7 kPa, 14), (7 kPa, 11), and (7 kPa, 11).

**FIGURE 8:**
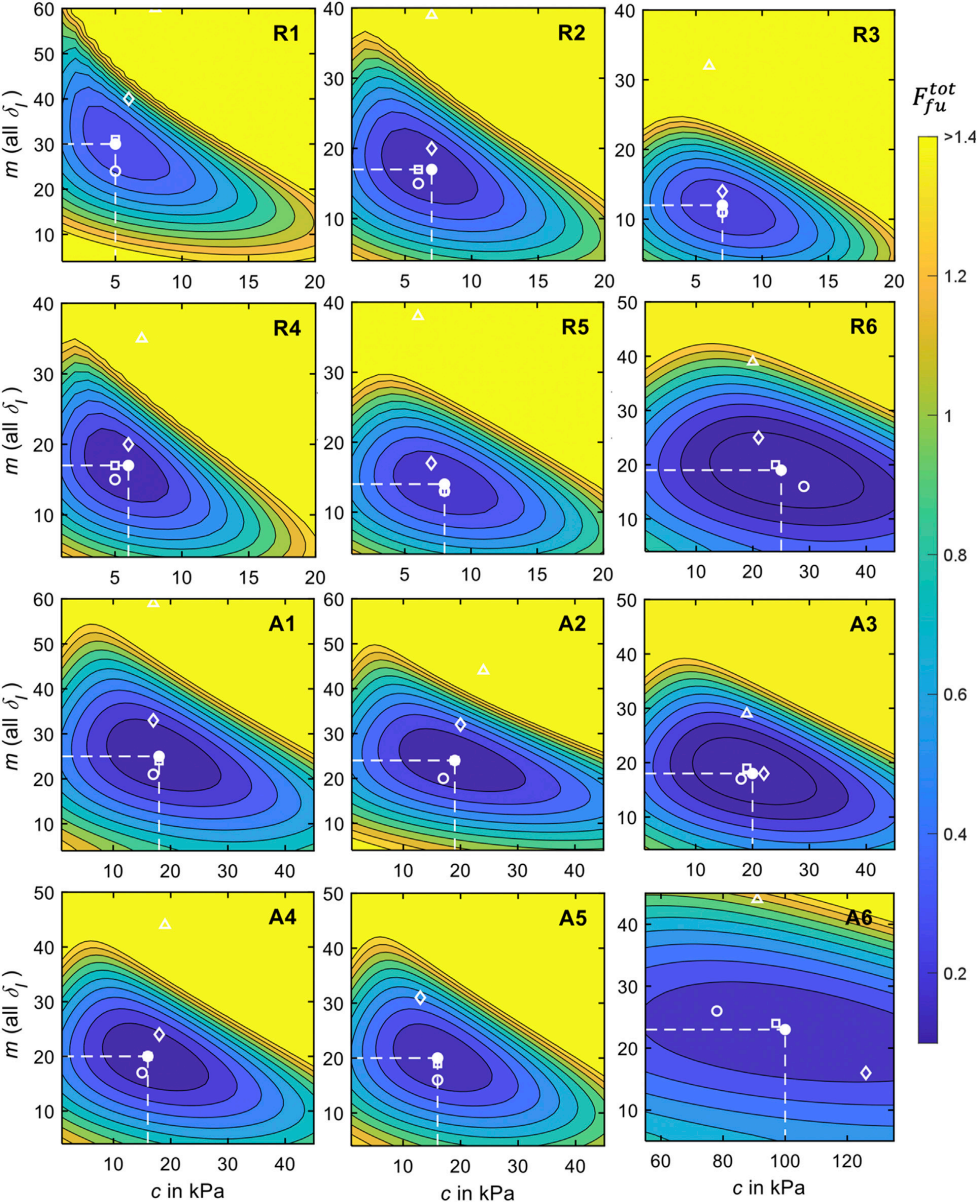
Results of the objective function analysis for all measurement regions and relaxed and activated musculature for η=0.5 . The contour plots depict the resulting shapes of $F_{fu}^{tot}\left(p\right)$ [*cf.* Eq. (10)] with white, unfilled markers indicating the resulting locations of min $F_{fu}\left(p;\delta_{I}\right)$ for $\delta_{I}$ from small to large: Triangle, rhombus, square, and circle (*cf.* Figure 7). Min $F_{fu}^{tot}\left(p\right)$ are each indicated with white, filled circles and the resulting material parameter sets $p_{res}$ from ***P*** are listed in Table 6. Certain parameter spaces are cropped for better visualization.

Four parameter spaces ***P*** with a total of 3321, 2565, 2565, and 2115 parameter sets for $\delta_{\max}^{*}=$ 13, 16, 20, and 25 mm were calculated respectively, depending on the properties of the experimental reference data. Sets with $\delta_{\max}^{*}=25$ mm took an average of 69.1 ± 23.84 s each. Figure 7 shows a representative example for the numerical interim results of the objective function analysis. The resulting shapes of $F_{fu}\left(p;\delta_{I}\right)$ are visualised as contour plots for each specific indentation depth step $\delta_{I}$ . The centers highlighted with markers define the minima of these contour plots and thus the respective parameter set $p_{I}$ , which represent optimal solutions for $\delta_{I}$ and η . The optimization results for η=0.5 (center column) together with the intermediate results (Figure 7) are compiled in Figure 8 for each measurement region investigated. The resulting material parameter sets $p_{res}$ at the minimum of $F_{fu}^{tot}\left(p\right)$ [see Eq. (10)] are indicated as well. All results are listed in Table 6.

**TABLE 6:** Resulting (best fit) Ogden material parameter sets $p_{res}$ with the parameter sets $p_{I}$ for the four simulated $\delta_{I}$ as ranges, and the respective values used in the inverse FE analysis. The identification of $p_{res}$ from the discrete parameter range P=C×M was carried out using the minimum of the objective function Eq. (10) with the reference parameters from Table 5. For *κ* see Section 2.8.

Measurement region	$\delta_{\max}^{*}$ in mm	$\delta_{trim}$ in mm	$f_{sim}$ in N for $\delta_{\max}^{*}$	$p_{res}$		$p_{I}$ range		$\min\left(F_{fu}^{tot}\right)$
				c in kPa	m	c in kPa	m	
R1	20	0.5	10.78	5	30	5–8	24–60	0.43
R2	25	0.6	13.11	7	17	6–7	15–39	0.33
R3	25	0.5	9.62	7	12	6–7	11–32	0.38
R4	25	0.5	11.24	6	17	5–7	15–35	0.31
R5	25	0.6	12.46	8	14	6–8	13–38	0.35
R6	16	0.6	19.60	25	19	20–29	16–39	0.12
A1	16	0.6	17.08	18	25	17–18	21–59	0.26
A2	20	0.5	30.37	19	24	17–24	20–44	0.29
A3	20	0.5	23.91	20	18	18–22	17–29	0.13
A4	20	0.5	21.07	16	20	15–19	17–44	0.26
A5	20	0.6	21.07	16	20	12–16	16–60	0.33
A6	13	0.4	59.48	100	23	78–126	16–44	0.34

**FIGURE 9:**
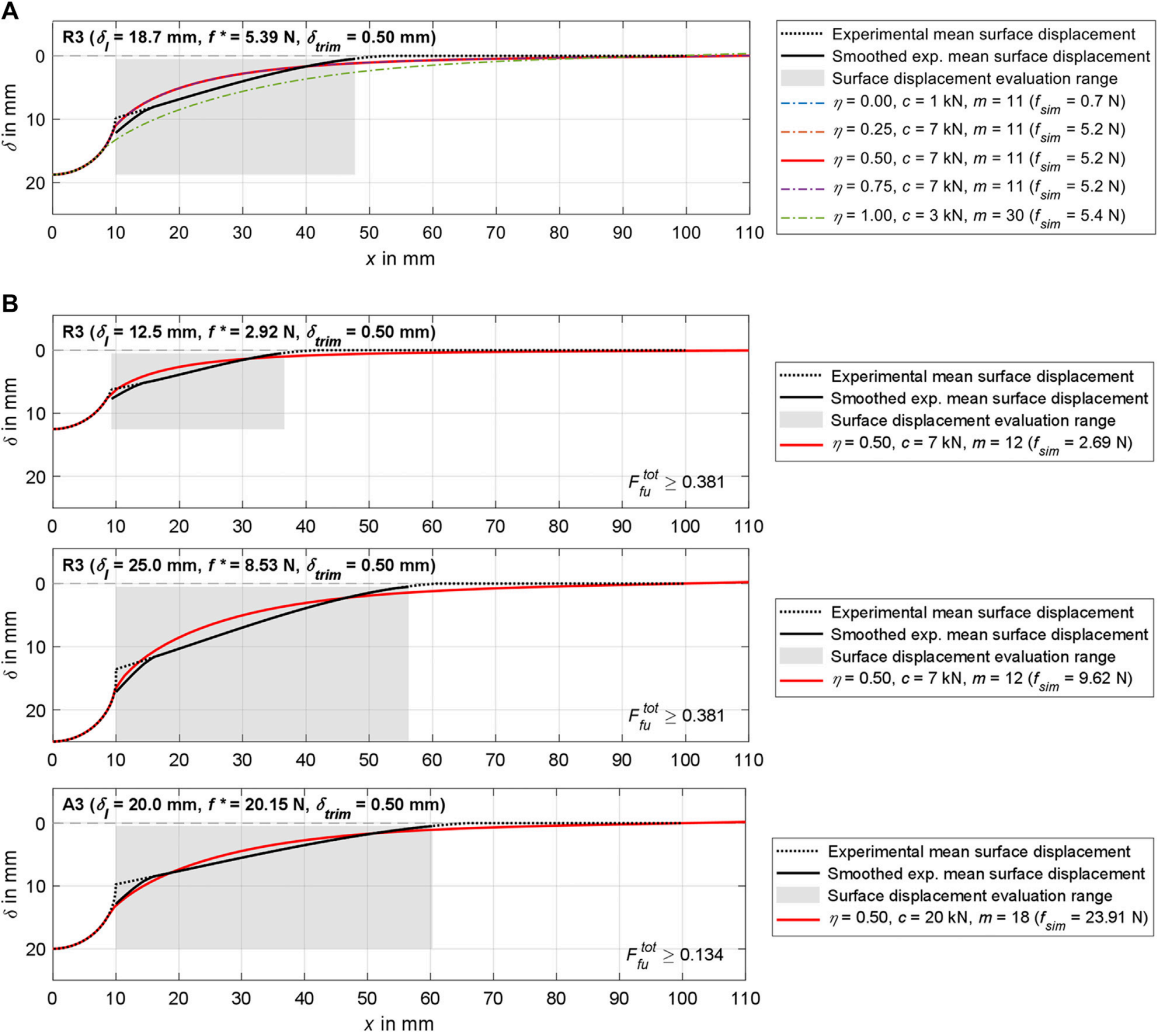
Visualization of simulation results for region 3 and various $\delta_{I}$ in comparison with the experimentally determined indentation forces and surface displacements. To calculate $F_{u}$ , only the smoothed section of the surface displacement (solid black line) within the evaluation range, marked in gray, was considered. **(A)** Contrasting the separate preliminary results for all five η (*cf.* Figure 7), the resulting $p_{I}$ , and $f_{sim}$ from which $F_{fu}\left(p;\delta_{I}\right)$ was calculated. **(B)** The solid red lines represent the surface displacements with the best fit Ogden material parameter sets $p_{res}$ from Table 6 for respective $\delta_{I}$ .

From the closed shapes of $F_{fu}^{tot}\left(p\right)$ , a unique $p_{res}$ could be identified for all regions with the optimization conditions used. The smallest absolute error occurred for R6 with $F_{fu}^{tot}=0.12$ and the largest for R1 with $F_{fu}^{tot}=0.43$ . Partial errors of $F_{f}\left(p;\delta_{I}\right)$ and $F_{u}\left(p;\delta_{I}\right)$ were accumulated in $F_{fu}^{tot}$ over all $\delta_{I}$ . As an example, for region 3, Figure 9 compares the FE results of the indentation FE model with the experimental data used. Deviations between the surface displacements are thus recognizable. However, their quantitative comparison for $F_{u}$ was conducted solely in the surface evaluation range indicated, between the intersection of the indenter tip profile and $\delta_{trim}$ (see Section 2.9). In general, a more concave surface deformation was observed for the material model used compared to some of the experimental data. The resulting material parameters for the different abdominal regions can be characterized as follows: With relaxed musculature c=6.6±1.14 kPa, m=18±7.04 anterior-lateral, and c=25 kPa, m=19 posterior. With activated musculature c=17.8±1.79 kPa, m=21.4±2.97 anterior-lateral, and c=100 kPa, m=23 posterior. The absolute deviations between $f^{*}$ and $f_{sim}$ were maximum at $\delta_{\max}^{*}$ with 3.4 ± 2.36 N.

## 4 Discussion

In this study, macro-indentation and time-of-flight skin deformation measurements were conducted to obtain data sets quantifying the *in vivo* material behavior of the physiological adult male abdomen. The data sets comprise the slow and continuous (non-ballistic) force-displacement curves of six abdominal regions, each synchronized with the displacements of the surrounding soft tissue resulting from the indentation. To account for the influence of the trunk muscles, the measurements were conducted with both fully relaxed and controlled activated muscles in various lying positions. Due to the small scatter range of the physiological parameters, standard values were calculated for all participants combined, which served as reference for inverse FEA. The inverse FEA was used to numerically estimate material parameters, which allowed the mechanical responses of the soft tissues to be simulated as a whole. All processed data sets and the material parameters of this study are described in detail or made available in the Supplementary Material S1–S4.

Our focus was on the *in vivo* characterization of the mechanical responses of the human abdominal wall and the structures it encloses in the abdominal cavity. During trunk muscle activation, the sections assumed to be linearized after foot regions of 3.5–8 mm were on average 5.2 N/mm posteriorly and 1.1–1.4 N/mm elsewhere (anterior-lateral). During relaxation, stiffnesses for small displacements were 0.72 N/mm posteriorly, and 0.24–0.34 N/mm anteriorly to laterally. The stiffnesses of the abdomen under large deformations (45% $\delta_{\max}$ , see Figure 4) were approximately 0.41–0.56 N/mm anteriorly, 0.62 N/mm laterally, and 2.31 N/mm posteriorly. This is in high agreement with previous measurements of local abdominal stiffnesses in which postures and experimental procedure were different (van Ramshorst et al., 2011; Tran et al., 2016). Although not statistically significant, the compliance with relaxed muscles is reduced anterolaterally (region 4) compared to the central abdomen (linea alba).

For each measurement region (Figure 2), we conducted an objective function analysis in which the mean experimental indenter force-displacement curve (Table 4) and the mean surface displacement (Table 5) served as the optimization targets. The accumulated differences between these and the simulation results were visualized using contour plots (Figure 8) and the convergence to a minimum was marked. Regarding the material stiffness and the surface displacement, the sensitivities of *m* and *c* [see Ogden strain energy density function in (5)] become apparent in Figure 7 for η=0 and η=1 . There is hardly any influence on the surface shape for varying *c* (horizontally orientated valleys). In case of η=1 , we observed vertically orientated deep valleys, which show that the material stiffness is not very sensitive to *m*. Only for modulation factors 0<η<1 , and thus with the superposition of tissue stiffness and surface deformation information, rounded and closed valleys resulted within the parameter space. For equal weighting, we evaluated all shapes and minima of the contour plots for η=0.5 . As already described by Oddes and Solav (2023) for synthetic material data, material parameter sets could only be determined reliably in our study with the consideration of $F_{f}$ and $F_{u}$ . Force-displacement data of the indenter alone is insufficient, although it is commonly used as the sole basis for parameter identification in inverse FEA (Fougeron et al., 2023). Frequently used alternatives are therefore multiple starting points optimizations, or genetic or evolutionary algorithms that are globally convergent (Oddes and Solav, 2023).

To the best of our knowledge, our study is the first of its kind, but has limitations in several aspects. Only young and healthy male participants with a similar BMI (Table 1) were involved in the study. As expected, we only found small inter-individual differences. Consequently, no general conclusions can be drawn about the entire population. However, other publications report significant differences in soft tissue behavior (Song et al., 2006; van Ramshorst et al., 2011; Sadler et al., 2018) as well as distribution of subcutaneous adipose tissue and body composition (Esparza-Ros et al., 2022) between women and men. Men’s tissue is often stiffer and less deformable. In 2011, van Ramshorst et al. (2011) reported that abdominal wall tension was on average 31% higher in men than in women, but that BMI had no significant influence. We were able to confirm this for the BMI, except for one participant with activated and pronounced abdominal muscles and the lowest percentage of body fat in regions 1, 4, and 5.

The experimental setup we developed (Figure 1) was accurate in terms of repeatability and absolute accuracy when compared to a mechanical testing machine with gelatine and 3D printed surface profiles. Overall, the test setup proved to be suitable for the aims of this study. Because the application of the test procedure is not limited to young male participants, data should also be gathered from other populations in the future, for example, women, people with a higher BMI, people with pathologies, and older people. This data could have a major impact on the computer-aided development of medical aids and products for which patient compliance is important. As only changes in the surface profile of the skin were measured using ToF, only tissue displacements in the axial direction could be captured and not, for example, strains. In addition, the initial positions and displacements of the soft tissues and organs of the abdomen were unknown in our study. Technically, these could not be determined with the current measurement setup. Supplementary MRIs or ultrasound scans can provide additional information to better categorize possible inter-individual variances, internal tissue deformations, and improve models with heterogeneous material or individual organs. The circumvention of physical limitations during MRI measurements has already been addressed and solved, for example, by Moerman (2012) by use of a custom designed, hydraulically powered, and MRI compatible soft tissue indenter.

Because both ToF sensors were mounted on the indenter housing, the field of view and thus the covered measuring range around the indenter tip was limited. As part of the data processing, we approximated laterally exceeding displacements of the skin with a multifactorial optimization using polynomial curve fits of third degree and base points (Figure 5). This procedure proved to be reliable, albeit time-consuming, because it had to be performed for each data set at the desired indentation depth. Nevertheless, in our opinion, this approach provides an accurate approximation of the total deformations under the assumption of an initially planar surface. In further studies, ToF sensors with a higher resolution could be mounted further away from the skin, or ToF sensors with a wider field of view could be used. Alternatively, laser scanning (Todros et al., 2019; Todros et al., 2020) or 3D digital image correlation (Moerman et al., 2009) would be accurate and reliable methods for superficial measurement of the abdominal wall, although they could make the experimental setup more complex. As interactions with skin are an important topic in modelling (Sanders et al., 1998; Portnoy et al., 2008), these should be examined more closely on the abdomen in the future. Detailed results from previous mechanical studies of the skin (Ní Annaidh et al., 2012) can serve as basis for this. However, due to the anisotropy of the skin, the orientation of the Langer lines is also relevant. To quantify the proportional influence of the skin on overall *in vivo* reactions, the effective movements of natural or applied surface patterns of the skin can be measured using digital image correlation (Szepietowska et al., 2023). Analyzing the displacement of the discrete structures of the overall image provides information about the anisotropic skin strains, which is not possible with ToF sensors that only return distance images.

In the current study, the sEMG signals were used to monitor whether the participants followed the instructions regarding muscle activation. Due to our primary aim of generating experimental reference data for the inverse FEA, we did not perform subject- or region-specific correlation analyses with other results. A potential shortcoming of the measurement in the anterolateral region 4 was that the supine position and movements in the lateral plane did not explicitly activate the abdominal oblique muscles (E2). Anterolateral crunches or a posterolateral position could be better suited in the future. For the anterior measurement regions 1-4, the participants were either fully extended or held their legs at a right angle with their lower legs horizontal. Due to the macroscopic nature of our study, we consider any subsequent influences on the tissue density and tension of the skin as a result of the postural changes to be negligible. The same applies to possible alterations in abdominal organ location, morphology, and rib coverage due to postural changes (Hayes et al., 2013a; Hayes et al., 2013b). The large anterior and lateral tissue deformations required ultrasound gel between indenter tip and skin, as the contact friction would otherwise have been highly unpleasant for the participants. In addition, this simplified the contact condition for the inverse FEA.

The present work is limited to one isotropic and hyperelastic material, thus the application to anisotropic and viscoelastic materials requires further research. For fat, the assumption of isotropic behavior is accurate (Dubuis et al., 2012), but the approximately 30 mm thick abdominal wall consists of numerous layers of muscles, tendinous structures, and blood vessels with fibers oriented in different directions (Song et al., 2006; Hernández et al., 2011; Deeken and Lake, 2017). However, the interaction of the layers decreases the degree of anisotropic response significantly (Hernández et al., 2011; Simón-Allué et al., 2017; Lohr et al., 2022). In addition, we used a 2° sector of an axisymmetric FE cylinder (Figure 6A). Further adaptations of the framework used in combination with FEBio and the opportunity to freely customize the codes offer a wide range of possibilities for more detailed inverse FEA. A first and already integrated approach should be the investigation of other hyperelastic material models (Mooney-Rivlin, Neo-Hookean, and Ogden-Moerman) that can be used for soft tissue modelling (Alawneh et al., 2022) as well as higher order Ogden models. There is also potential for improvement in terms of computational efficiency and model stability. Even though our study showed that a first-order Ogden model already provides good approximations for all regions, the numerical results for larger deformations offer the potential to be closer to the experimental data (Figure 8). Because the volumetric behavior depends on the bulk-like modulus and we determined it via a ratio to the varied material parameter *c*, its influence on the FE surface deformation is to be analyzed systematically in further studies.

Another simplification worth mentioning is the planarization of the locally compressed abdominal surface, here in the form of a right circular cylinder. As was also evident in the ToF raw data, the undeformed measurement regions were mostly cylindrical surfaces, but were converted to a planar reference plane during data processing. This means that all $x>x_{\max}$ (Table 5) were assumed not to be displaced and a comparison with the FE cylinder was made possible [*cf.* Eq. (8)]. Depending on ***p*** and *δ*, it could be seen that areas near the outer edge of the FE cylinder shifted in the direction opposite to the direction of indentation (*cf.* Figure 9). This material uplift was due to the compensation of the central uniaxial compression and the finite cylinder diameter. As the uplifts were <0.35 mm at the outer edge for all $p_{res}$ and $\delta_{\max}^{*}$ , we consider them to be negligible. To compensate for this, the cylinder radius would have to be increased, $\delta_{\max}^{*}$ reduced, or an additional boundary condition applied to the cylindrical surfaces. For buttock compression with a plate, for example, Moerman et al. (2017) have shown that geometric differences influence the results of an inverse FEA. This is one of the reasons why an FE model with a cylindrical surface, that mimics the participant-specific curvature of the abdominal wall, could provide an even more accurate description in the future. The 3D surface shapes required for this can be obtained using the integrated ToF sensors (Figure 5A).

The use of four equidistant indentation depth steps $\delta_{I}$ is another shortcoming of our inverse FEA approach. For an optimal numerical approximation of the experimental material behavior, more than one material parameter set $p_{I}$ for each of the four $\delta_{I}$ was required for all measurement regions. In the example of R3, $p_{I}$ resulted in $p_{1}=\left(6\text{ kPa},32\right)$ and ${p_{3}=p}_{4}=\left(7\;kPa,11\right)$ , among others. Assuming that the abdominal material response under compression is continuous, $p_{res}$ is therefore only an approximation over all $\delta_{I}$ . A higher amount of discrete $\delta_{I}$ can improve the numerical accuracy of the overall result. In contrast to synthetically generated reference data (Oddes and Solav, 2023), which are based on an already known material parameter set, the experimental reference data of the surface displacements for the respective $\delta_{I}$ was calculated independently from all participants as an average value. The multi-criteria optimization approach [see Eq. (2)] does not ensure that the individual curve fits can be described using a material model in a way that a $p_{res}=p_{I}$ exists for $I\in N^{+}$ . In addition to a continuous determination of $p_{res}$ over the entire indenter stroke, the surface displacement in particular should not be determined independently for the $\delta_{I}$ used in future.

It should be noted that we focused on the transient material response during indentation and thus did not consider tissue relaxation. All measurements were carried out with the same indentation speed. Each test run was completed quickly and continuously in less than 16 s (extension and retraction of the indenter tip with 40 mm maximum travel), so the reaction was considered as an instantaneous response (Yu et al., 2006; Lu et al., 2009). The fact that the participants had to hold their breath and purposefully activated their muscles, however, prohibited slower tests (<5 mm/s) and prolonged holding of the indenter tip at maximum stroke (>1 s). Deep inhalation lifted the abdominal wall and the muscle activity was measurably increased. Limited by the indenter being in continuous contact with the skin, no active breathing could be examined and separate runs would be necessary. We therefore assume increased abdominal stiffness during inhalation, which should be investigated in further studies.

Modelling viscoelasticity is straightforward and can be carried out, for example, using an elastic component coupled with a viscous component, which acts as a damper that delays the stress-strain response (Wells and Liang, 2011). The use of our data for the validation of complex models, that represent the abdomen in detail, is also conceivable. Along with exact anatomical models, patient-specific material that takes biological data into account, is becoming increasingly relevant in biomedical engineering and treatment (Erdemir et al., 2006; Moerman et al., 2016; Simón-Allué et al., 2017; Macron et al., 2020; Lerchl et al., 2022). By averaging over all participants and discarding the anatomical characteristics, we are not yet able to make statements beyond the physiological standard values. Further work will seek to address subject-specific material parameters from inverse FEA using *in vivo* indentation data and tissue displacements.

A long-term goal of our work is to assess biomechanical principles of action of the lower back to improve the development of biomedical products specifically in the early design phases. We aim to achieve this by simulating the interaction between a digital human body model and a virtual technical system. This facilitates the methodical variation of the properties of the technical system as part of the engineering design process with regard to ethical and economic factors. Moreover, even the latest experimental methods are limited when it comes to studying internal mechanics such as muscle forces or stress states in soft tissues, the overall context of the stabilizing functions of individual muscles, and thus the possible causes of pain (Remus et al., 2021). To support the engineering design process, a new type of digital model of the lower trunk aims to couple a muscle-driven forward dynamical active hybrid model of the lumbosacral spine (Remus et al., 2023) with the surrounding soft tissue of the abdomen. In this regard, the work presented here contributes to a better understanding and numerical quantification of abdominal *in vivo* soft tissue behavior under local compression.

## 5 Conclusion

In this study, we presented a novel, simple, and reliable method for *in vivo* measurement of the mechanical soft tissue behavior of the human abdomen. The force-displacement curves, the associated surface displacements of the skin, and the muscle activities of ten males were measured in a total of six regions, in each case with completely relaxed and with controlled activated muscles while lying horizontally. The experimental data allowed us to identify similarities and significant differences between the regions and states of muscle activation. In addition, we used inverse FEA to derive unique hyperelastic material parameter sets that numerically approximate the experimentally measured soft tissue behaviors. This comprehensive *in vivo* dataset is not available in the current literature and represents an advance in our knowledge of abdominal material properties, enabling improved numerical human body models, interaction studies, and product development processes.

## Data Availability

The original contributions presented in the study are included in the article/Supplementary Material, further inquiries can be directed to the corresponding author.

## Supplementary material

SUPPLEMENTARY MATERIAL S1 Complete data set of coefficients and goodness of fits of the polynomial curve fitted experimental force-displacement curves $f\left(\delta \right)$ .

SUPPLEMENTARY MATERIAL S2 Exemplary visualization of the trim factor $\delta_{Trim}$ evaluations best suitable for the objective function analysis of measurement region 3.

SUPPLEMENTARY MATERIAL S3 Visualization of the experimentally determined raw force-displacement curves (left) and the participant-related curve fits calculated from these (right) for all regions.

SUPPLEMENTARY MATERIAL S4 Experimental mean surface displacements and ranges for all measurement regions at $\delta_{I}$ .
