# Supplementary figures and images for: Soft tissue material properties based on human abdominal in vivo macro-indenter measurements
Source: Front Bioeng Biotechnol. 2024 May 24;12:1384062. doi: 10.3389/fbioe.2024.1384062 (PMC11157078; doi:10.3389/fbioe.2024.1384062)

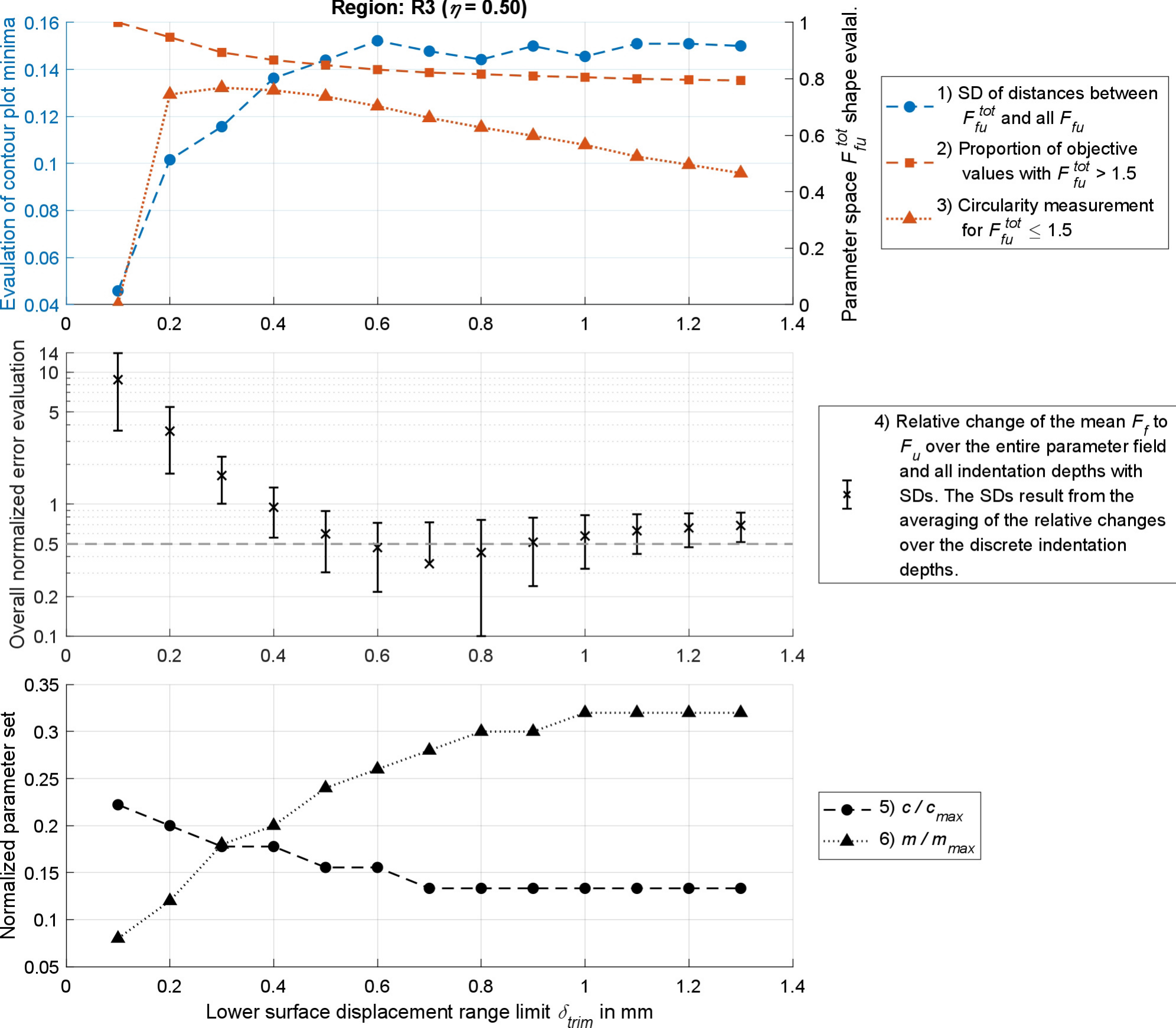

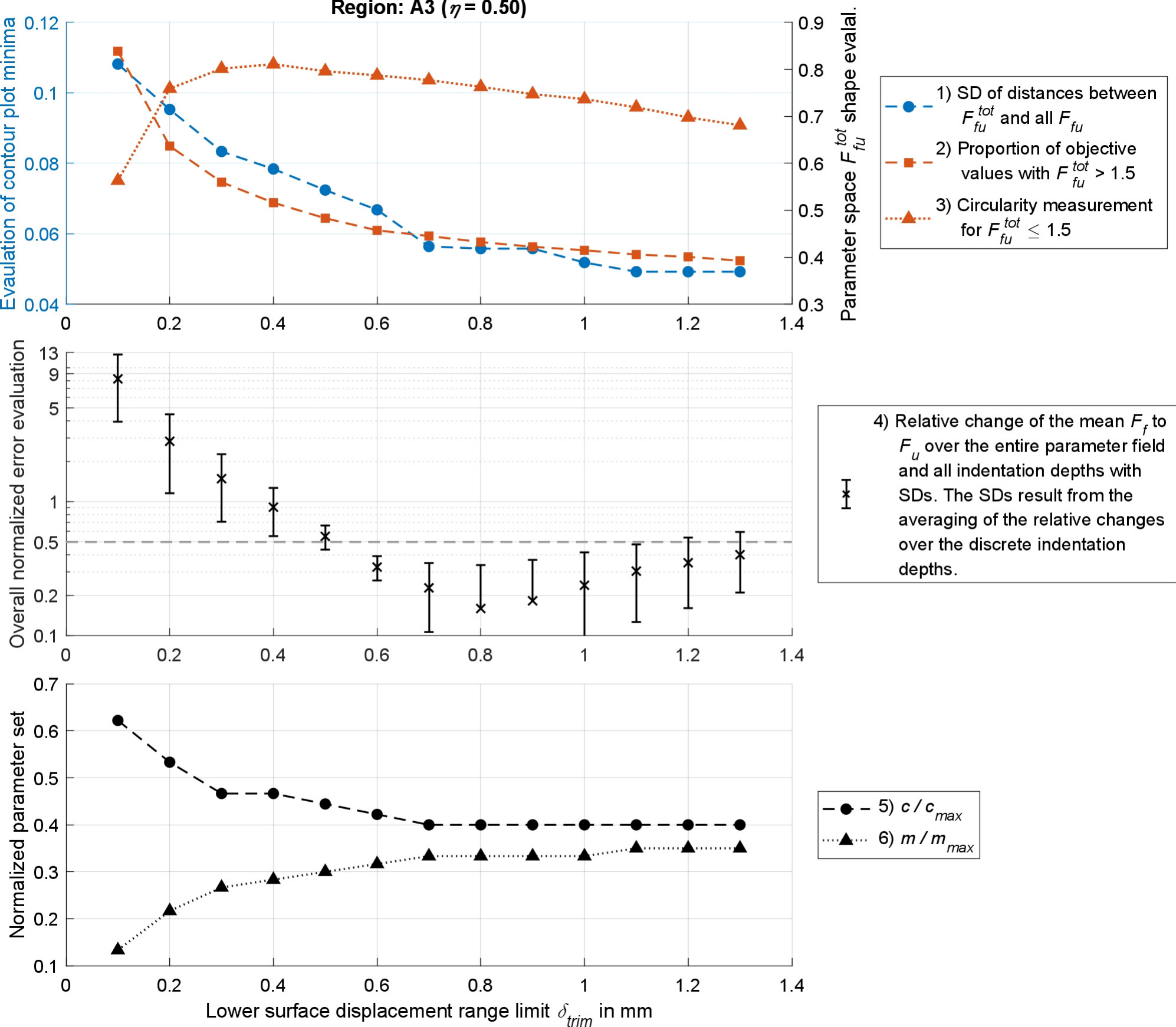

Supplement: Supplementary file 1 [file DataSheet2.PDF]

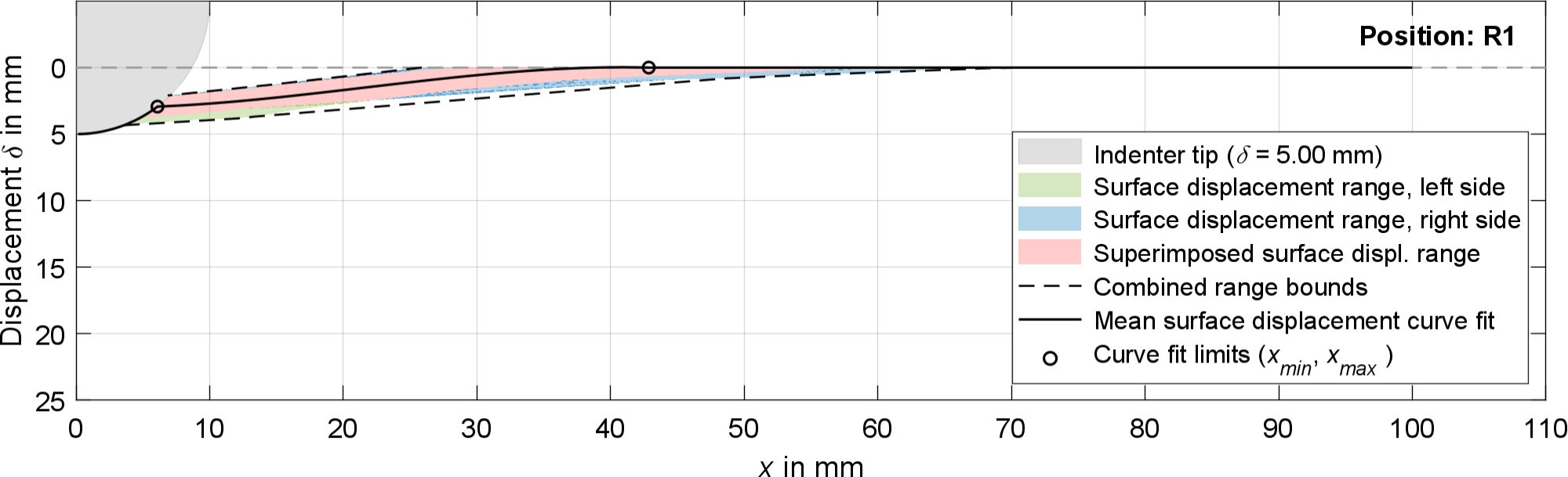

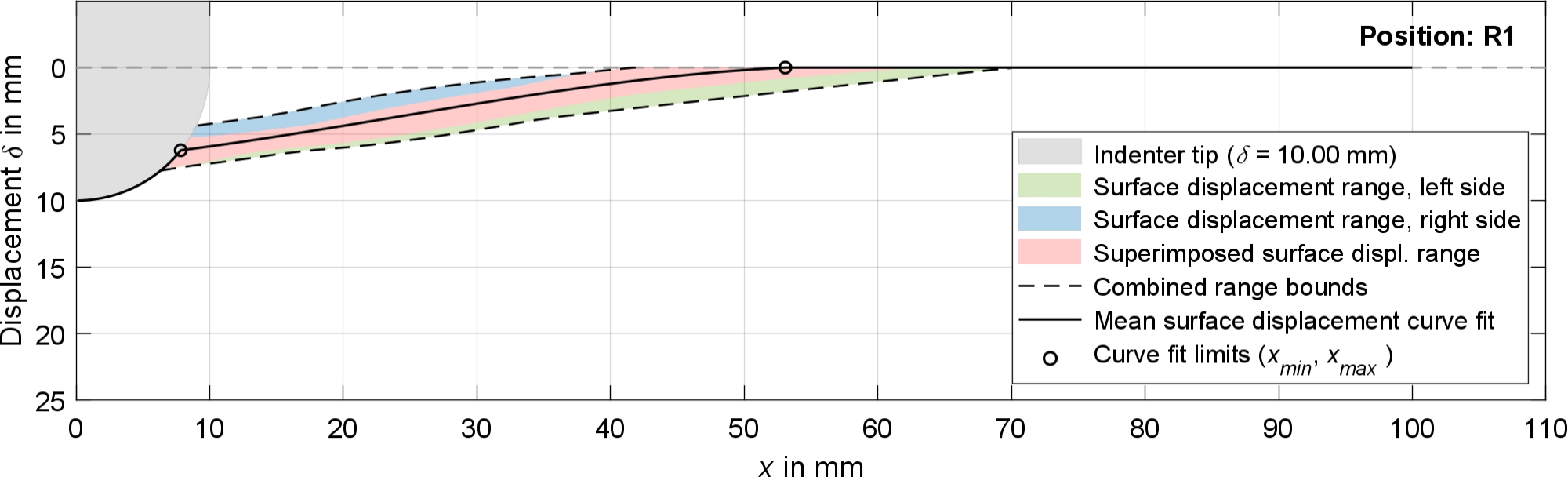

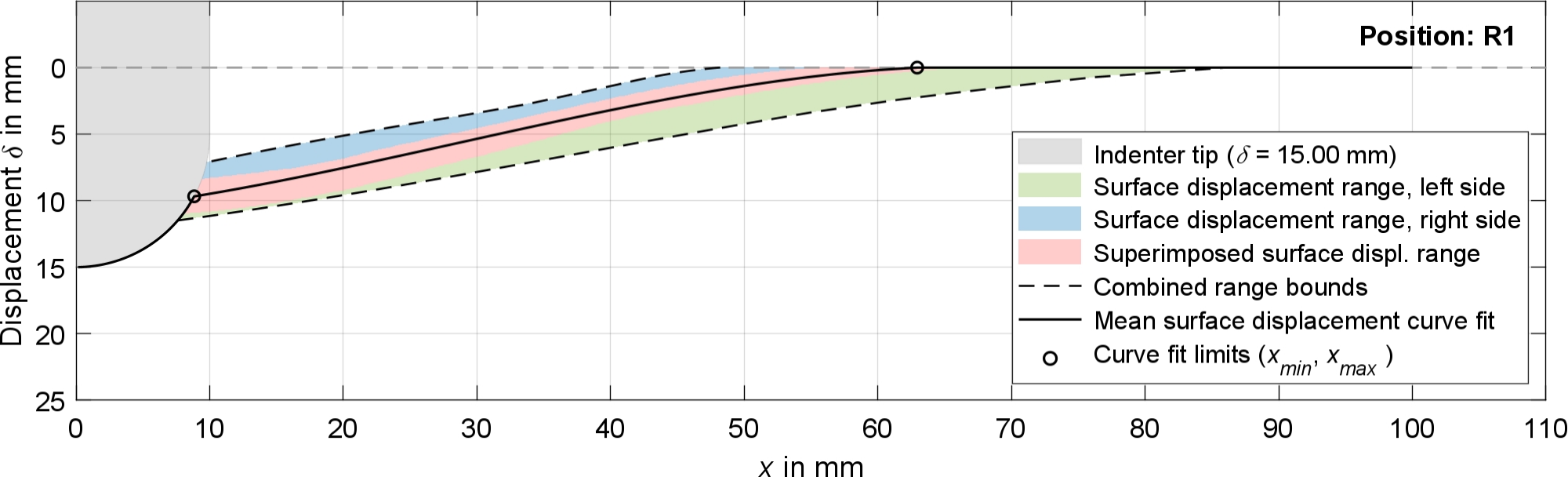

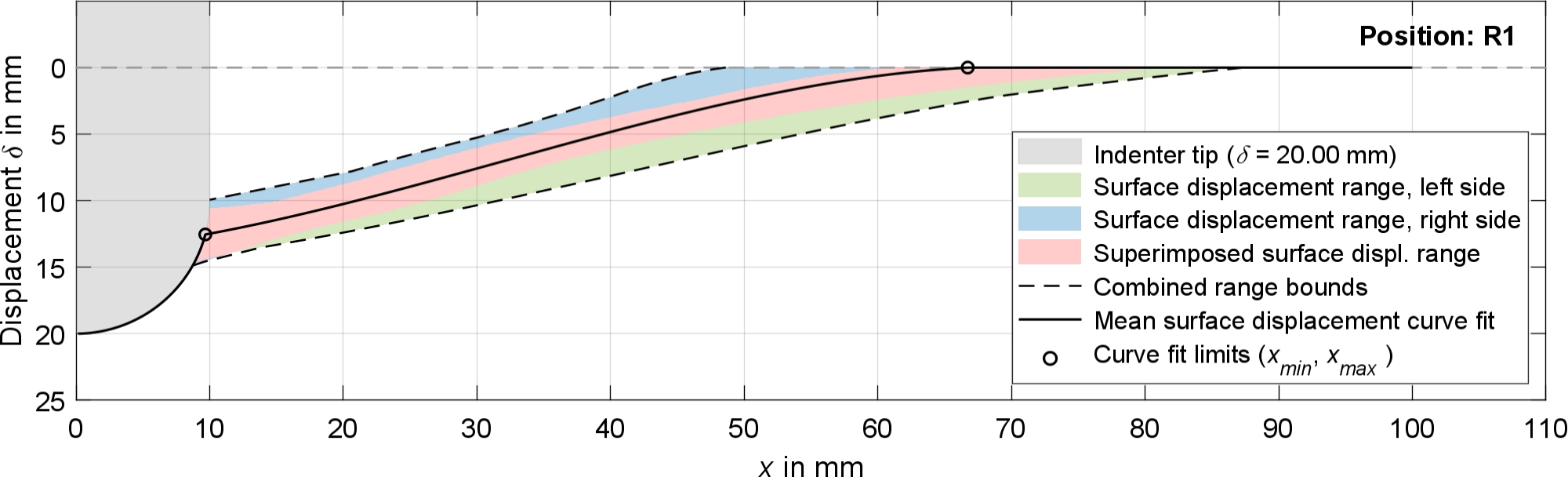

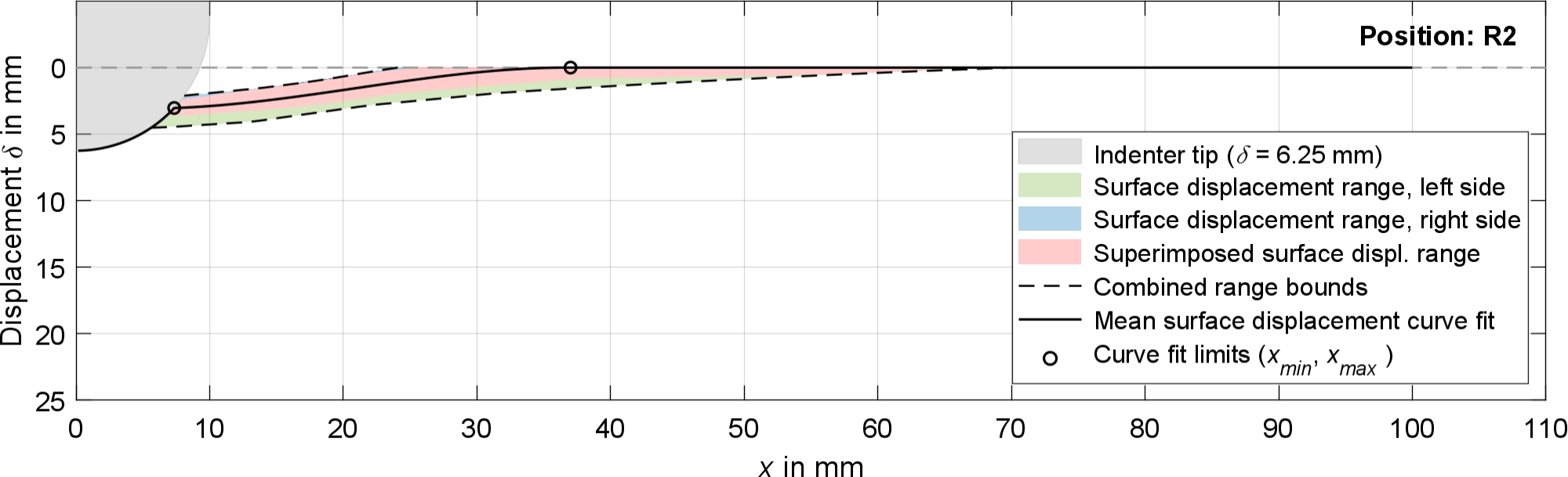

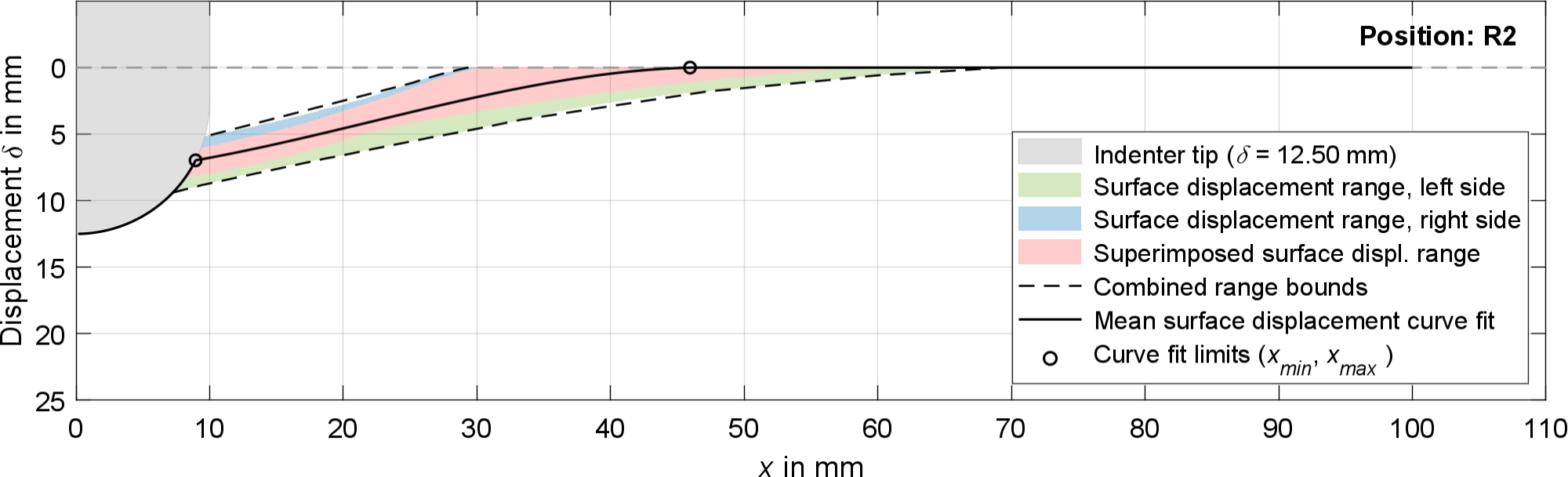

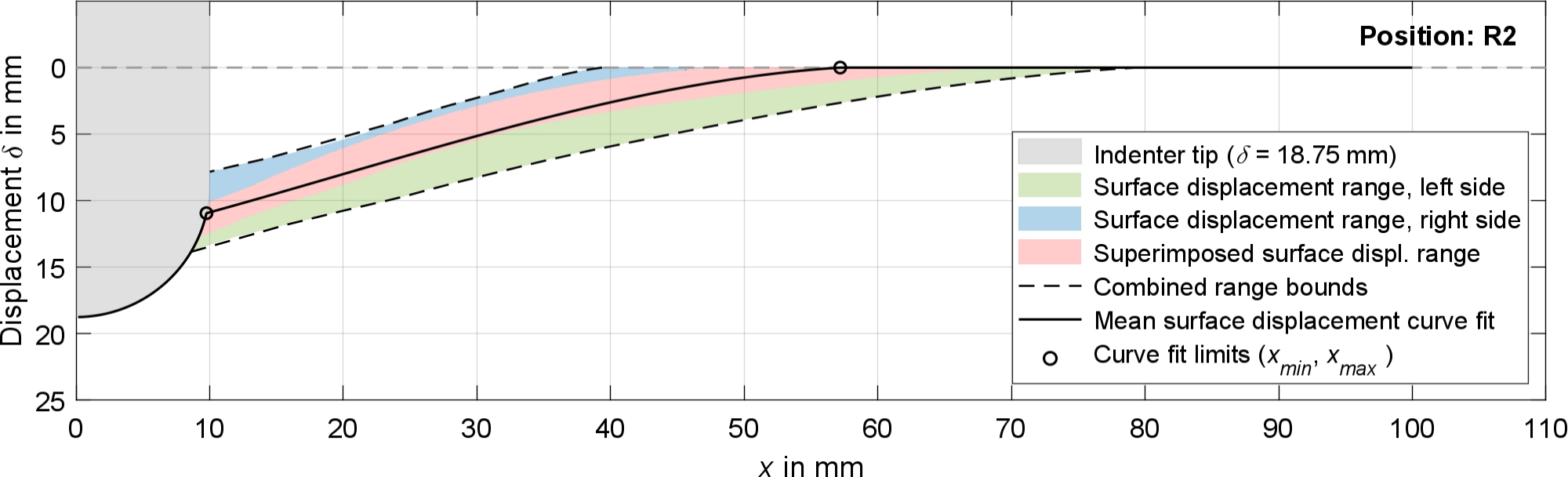

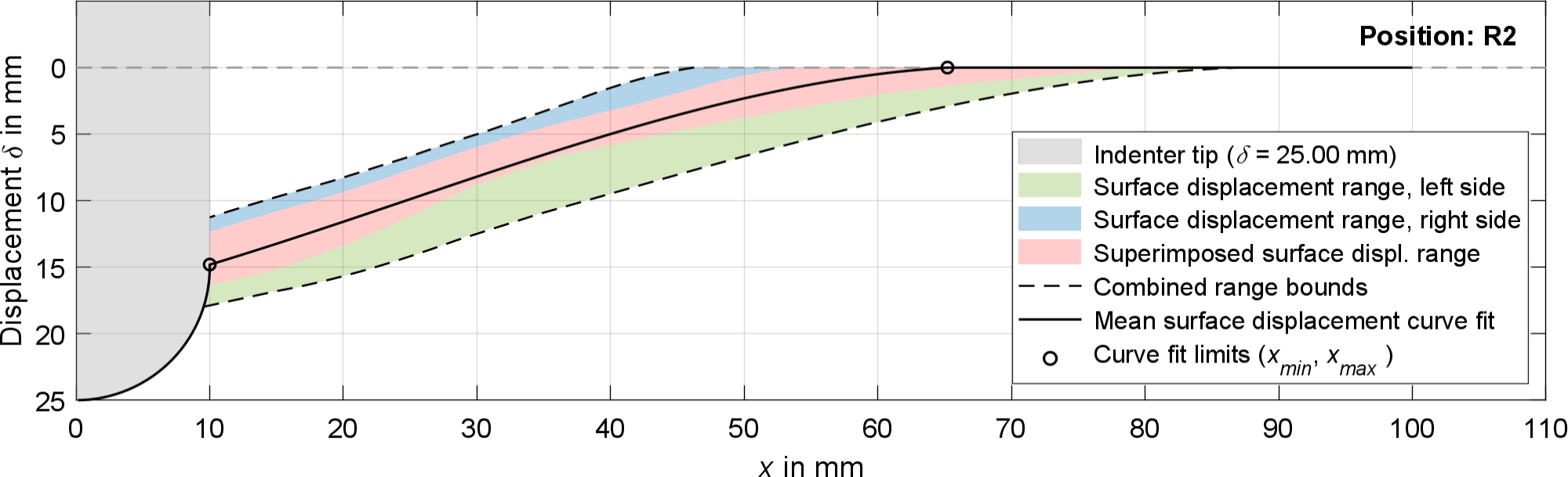

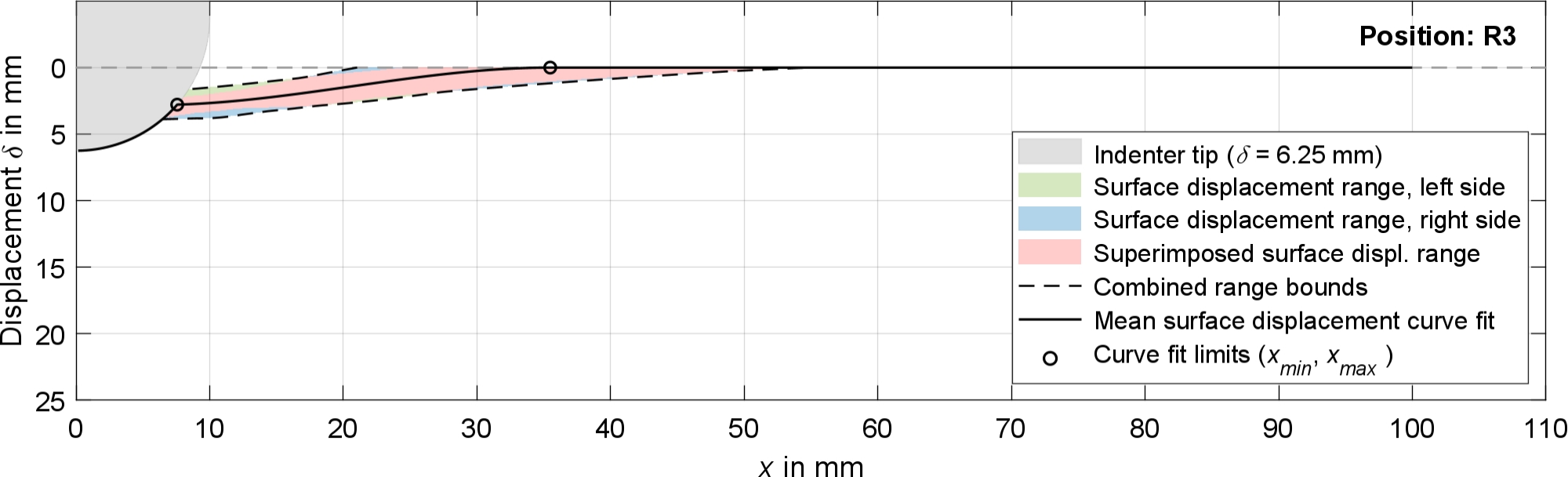

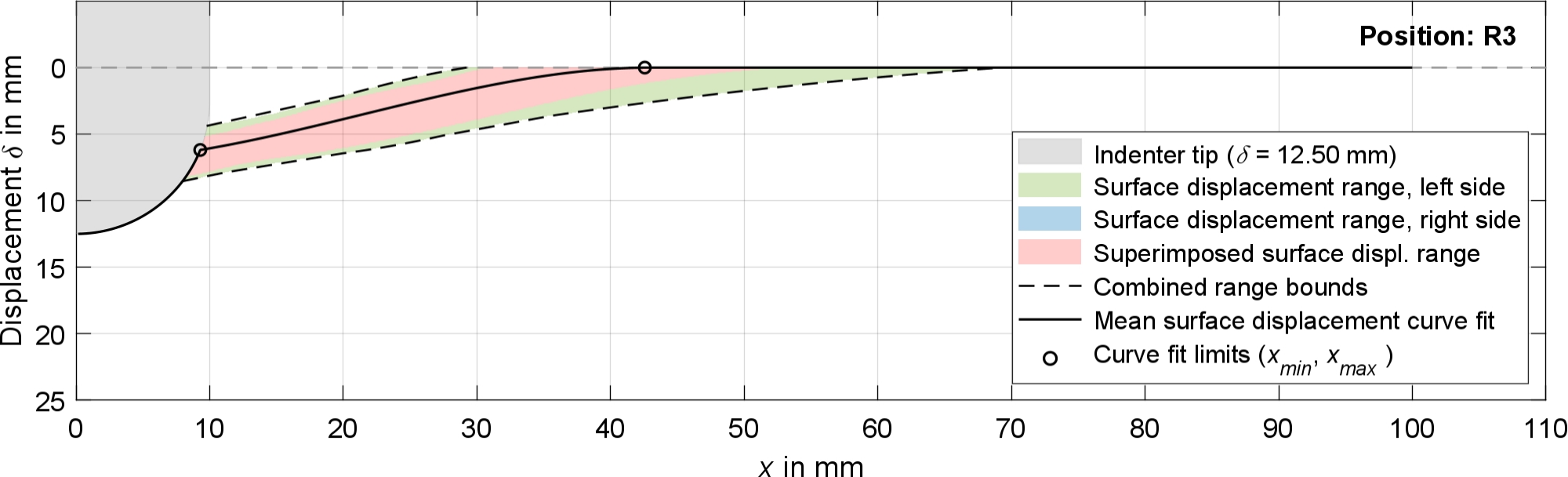

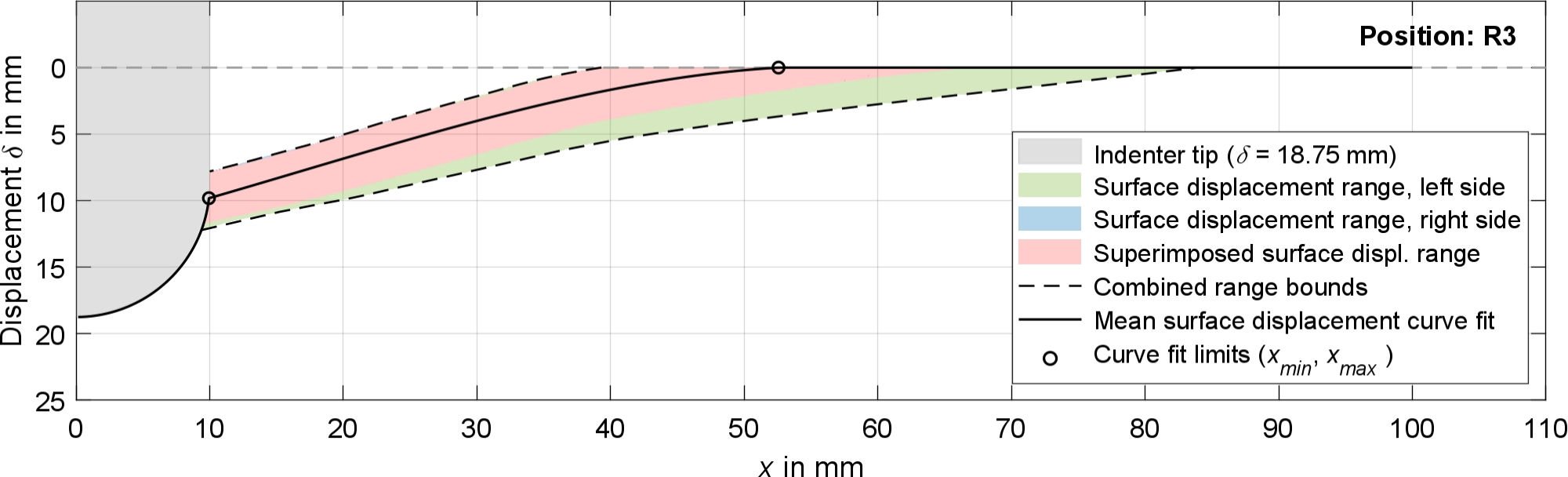

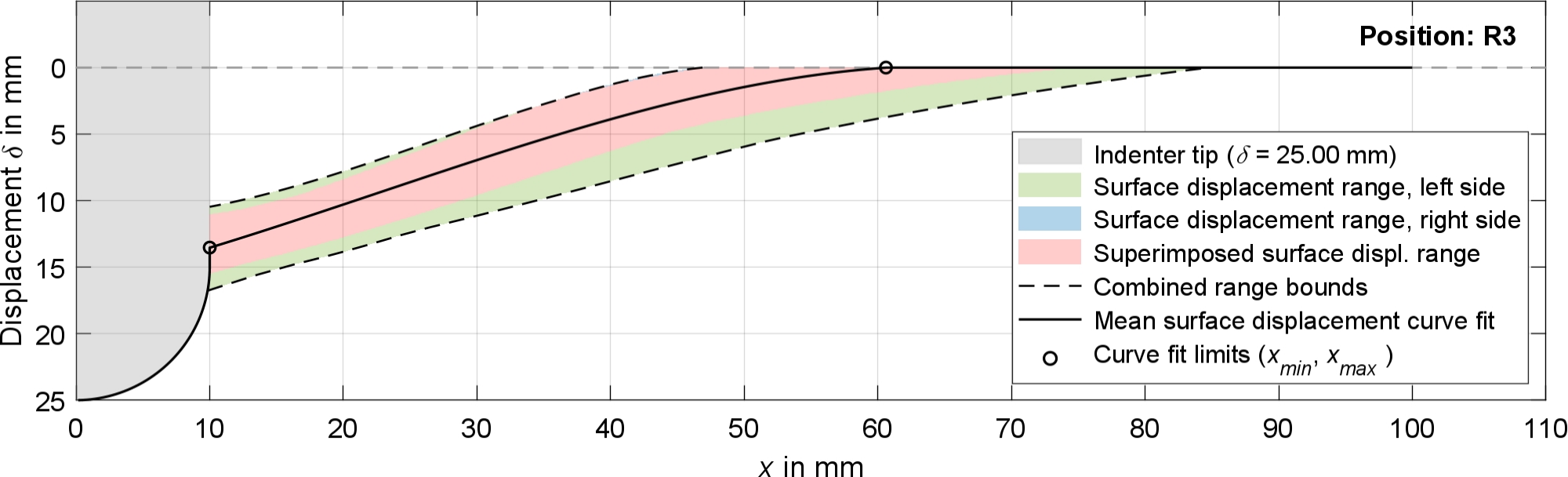

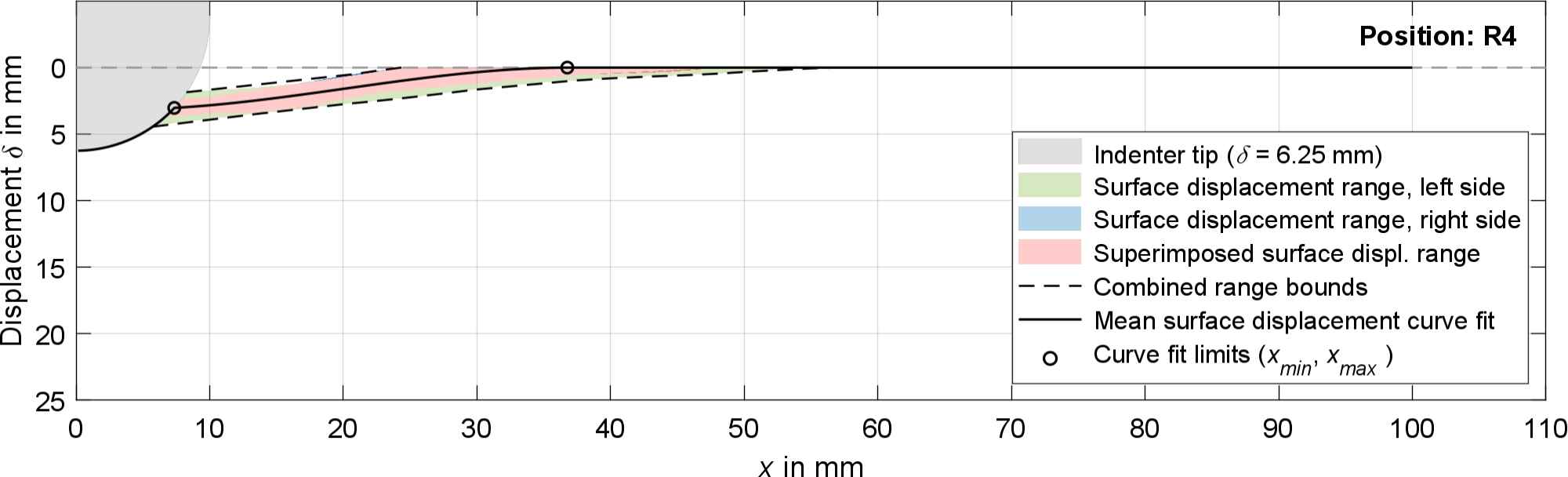

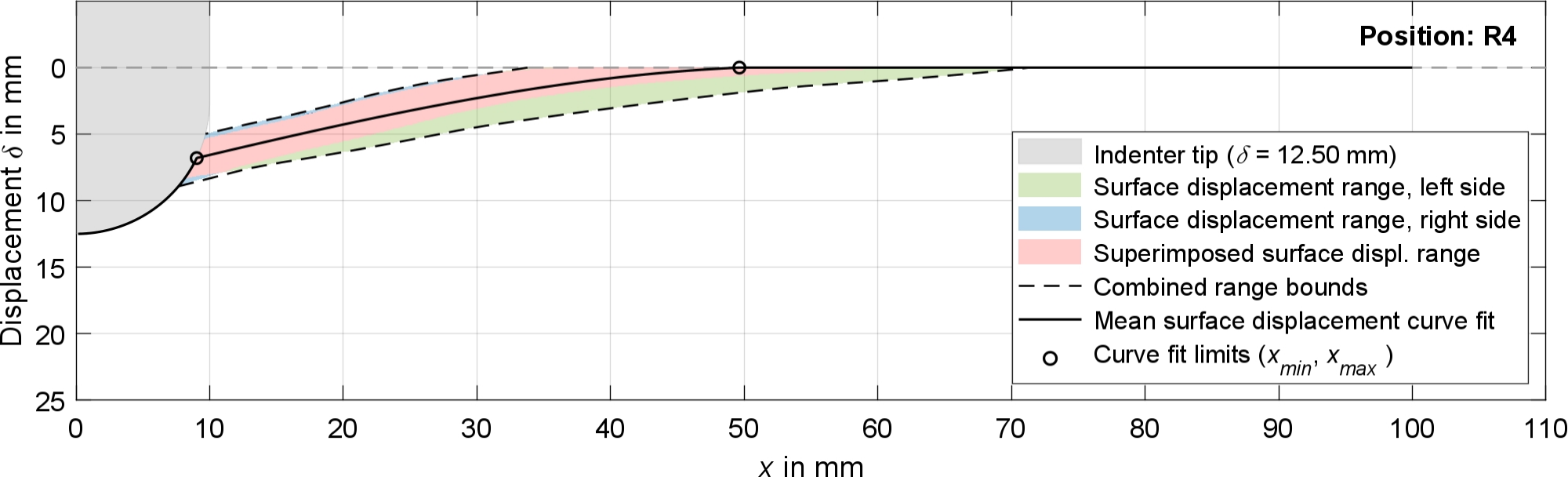

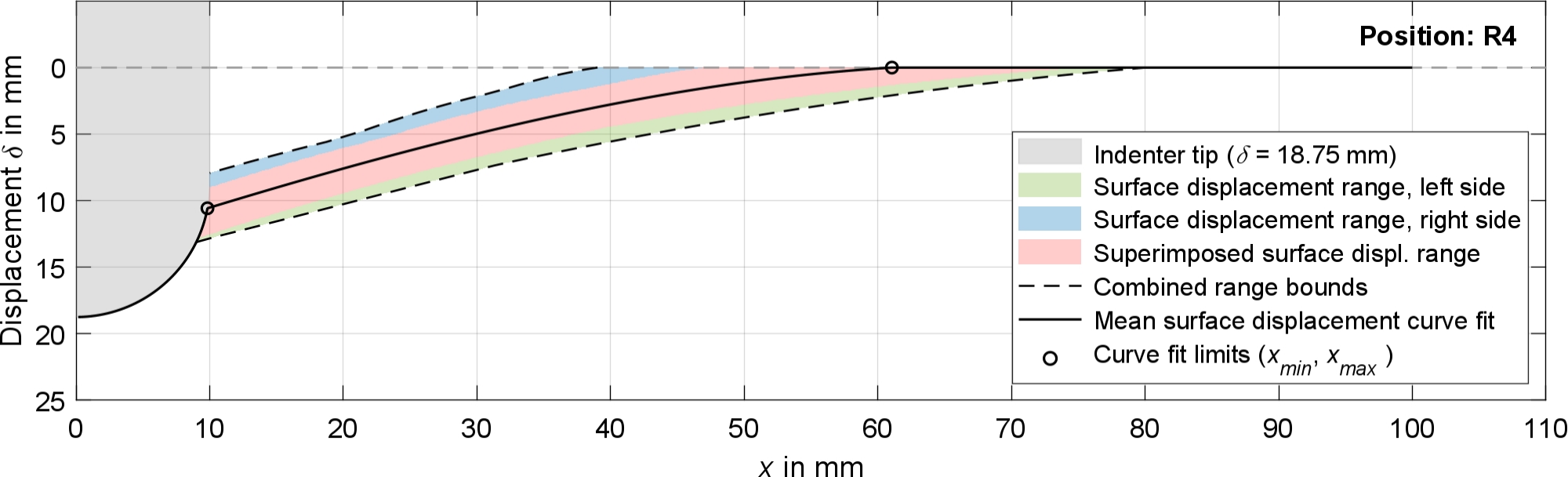

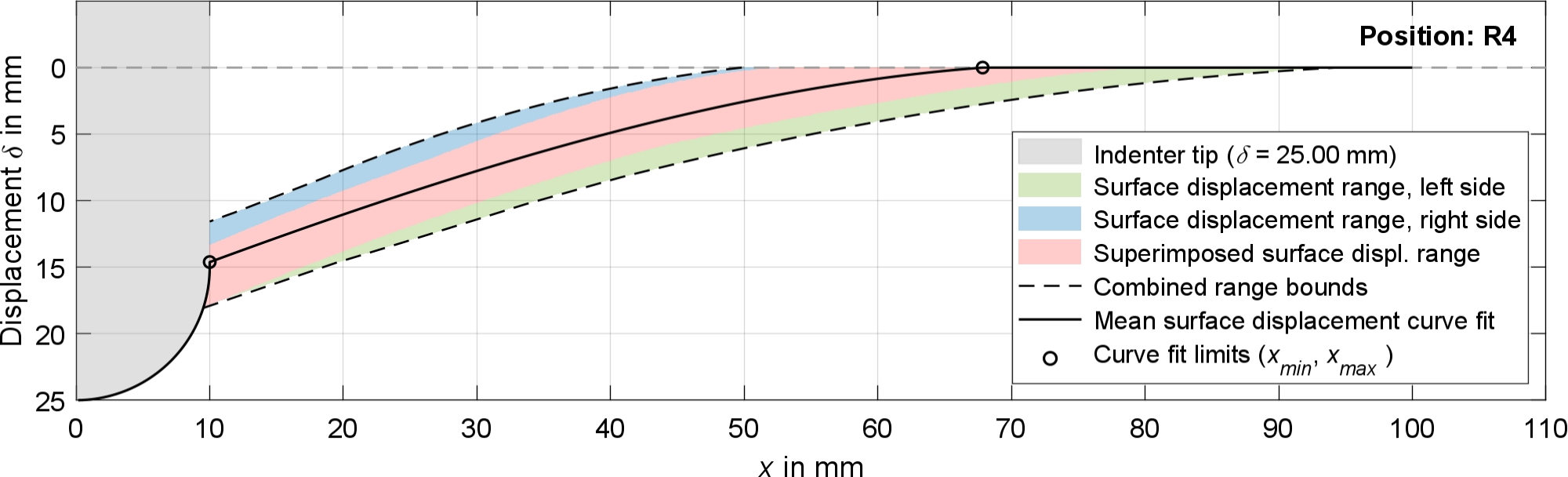

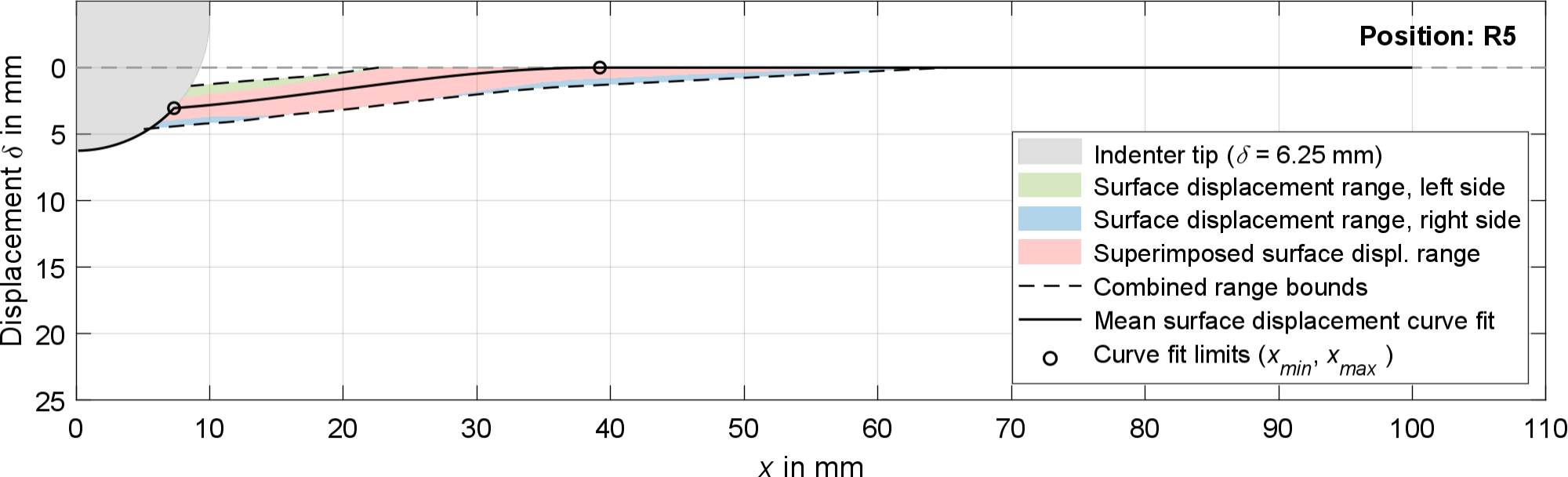

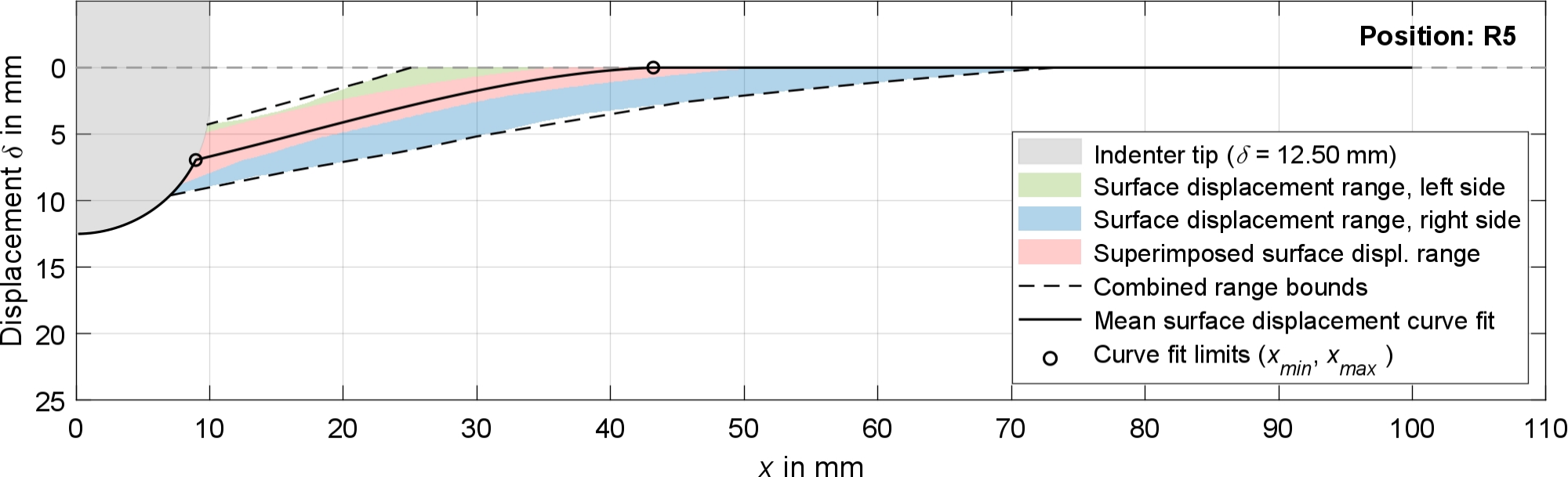

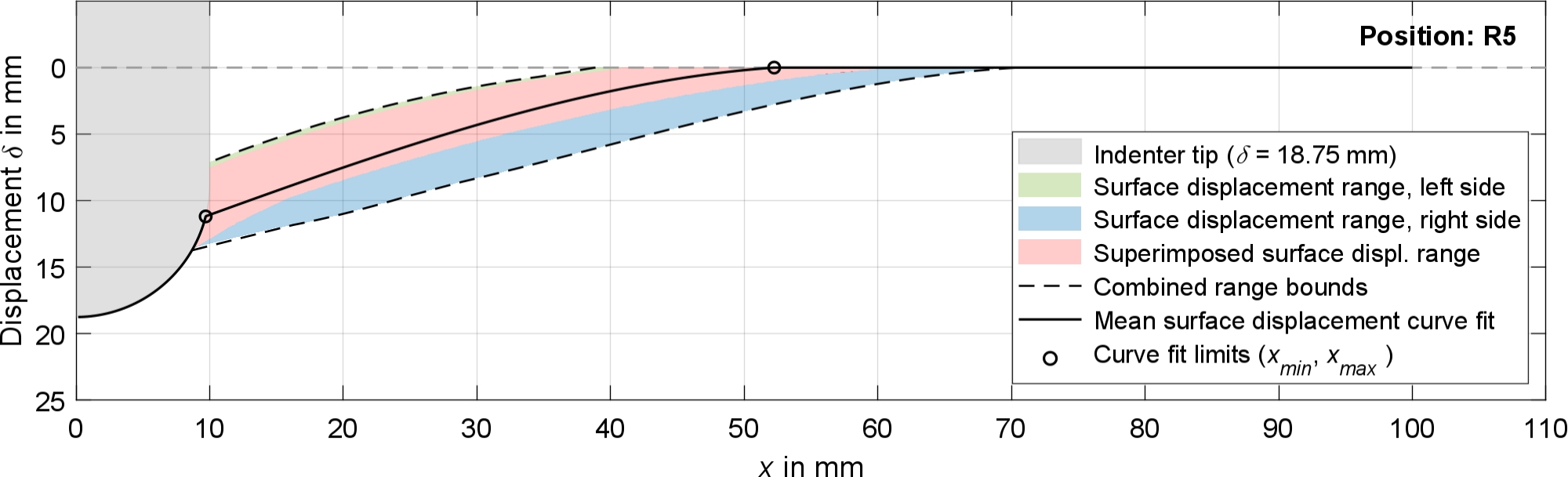

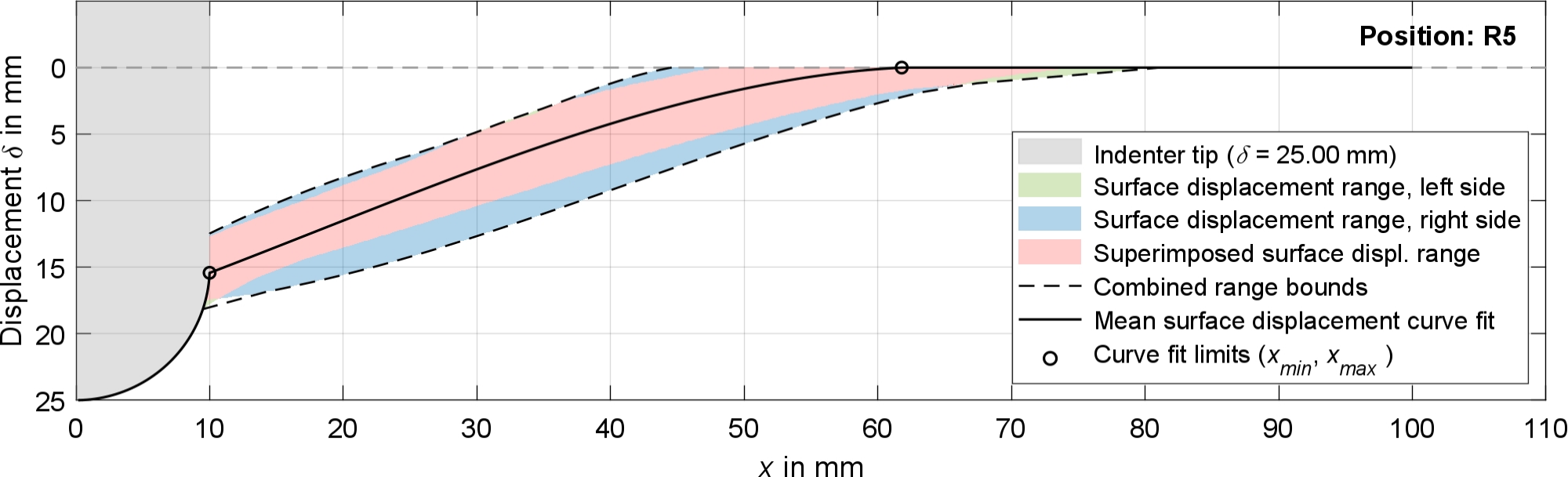

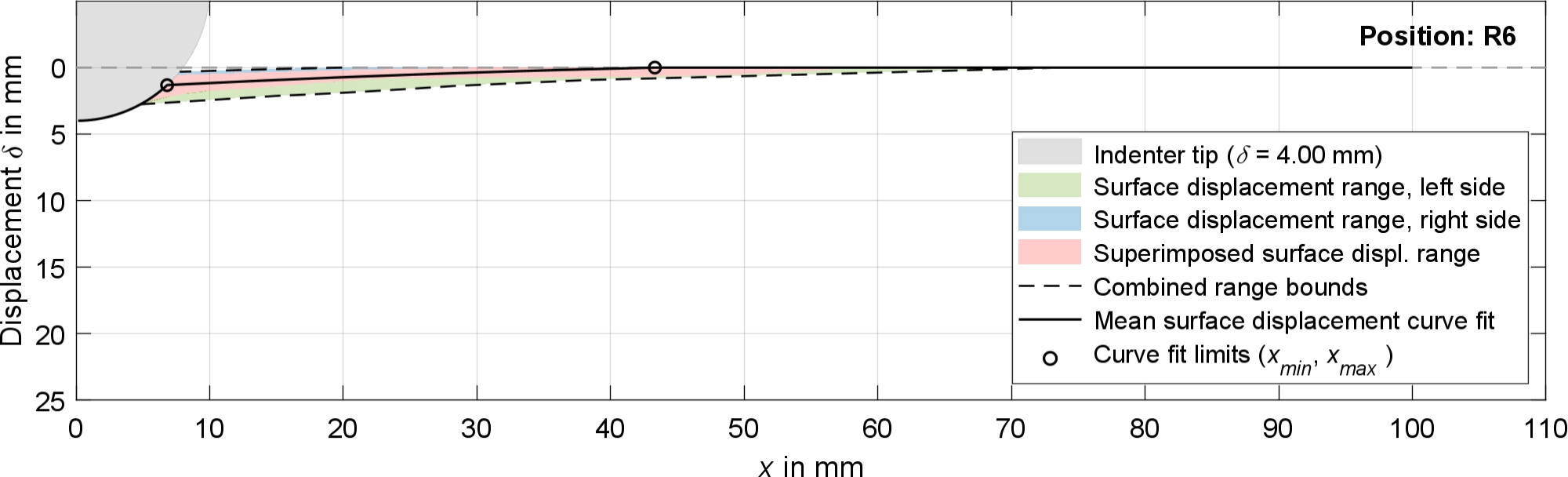

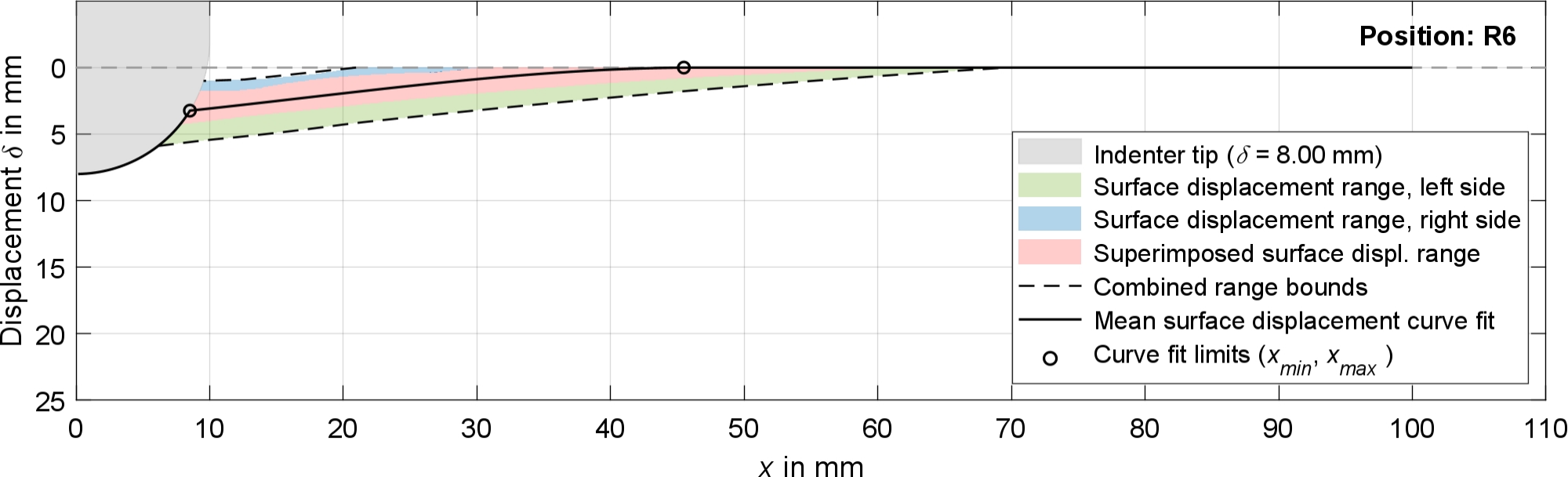

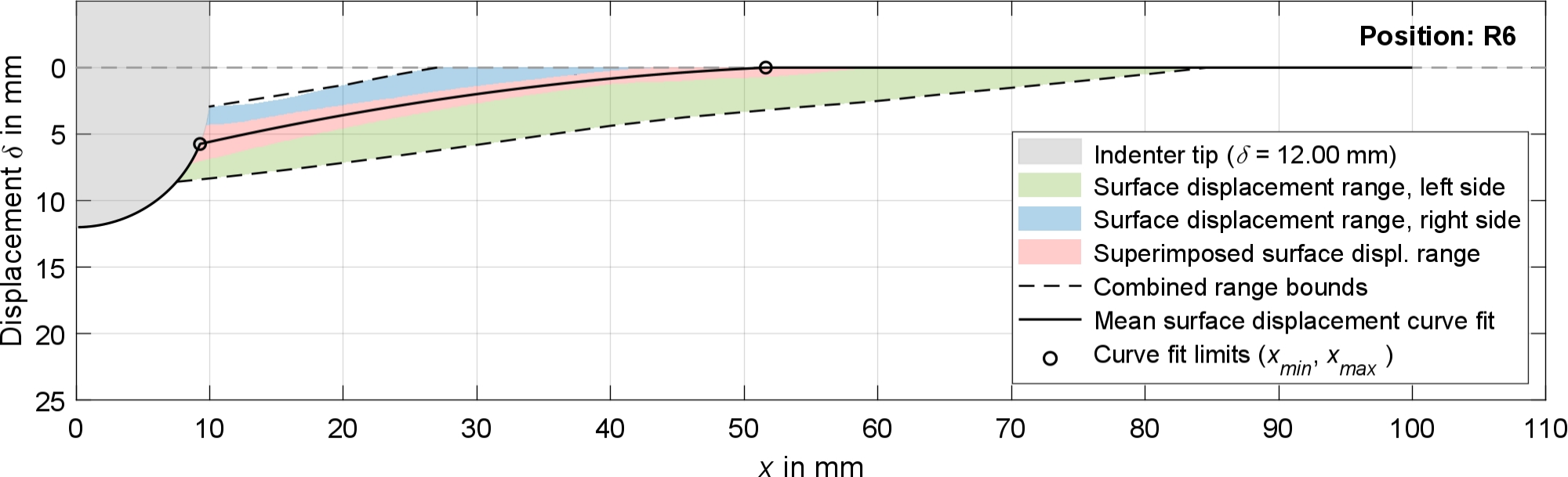

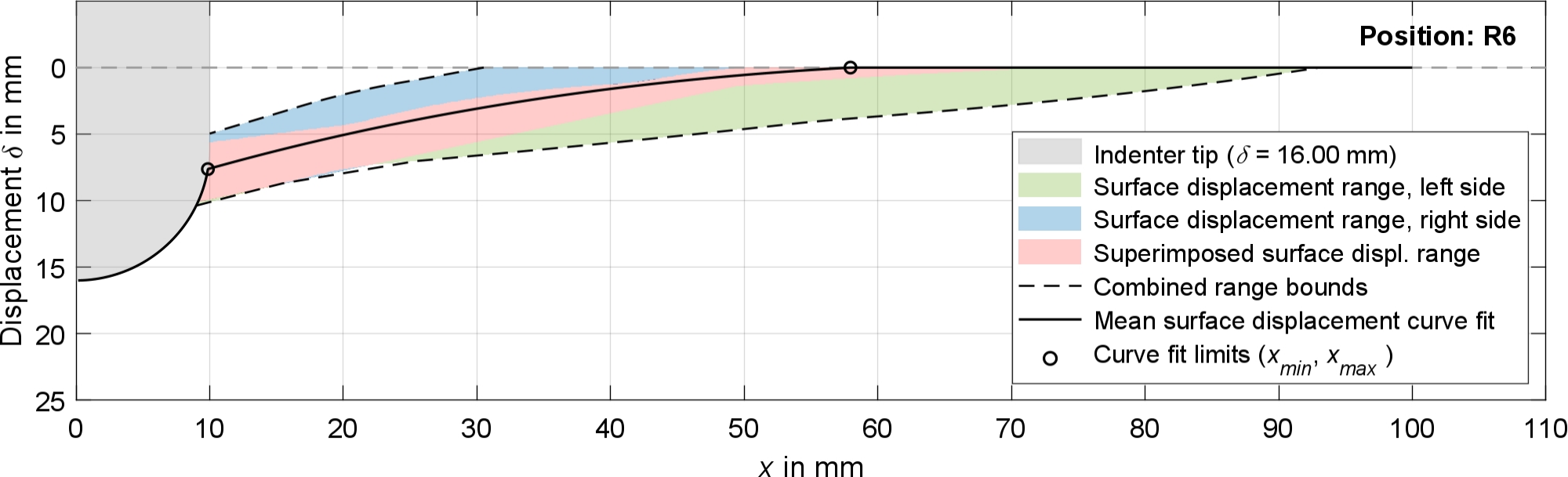

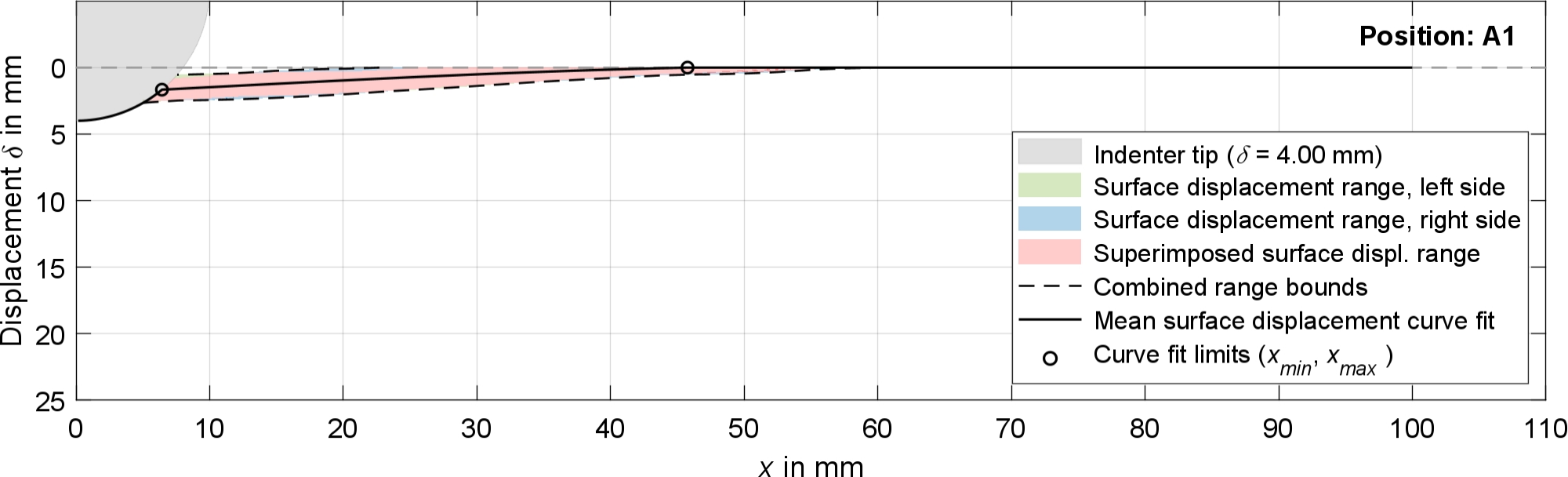

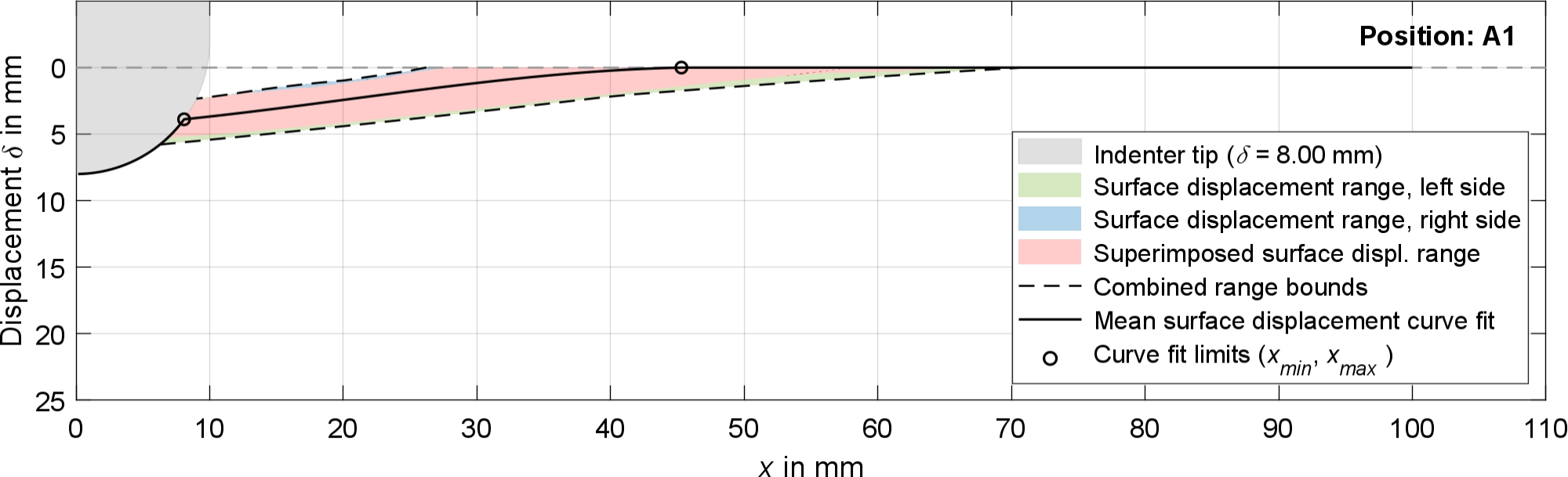

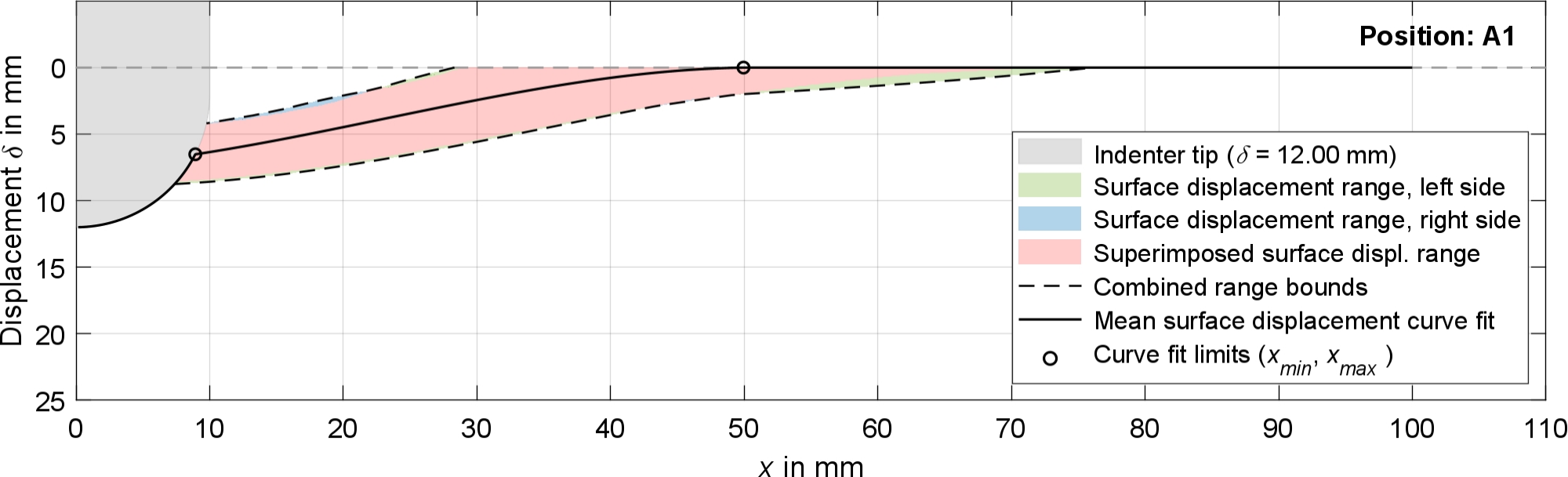

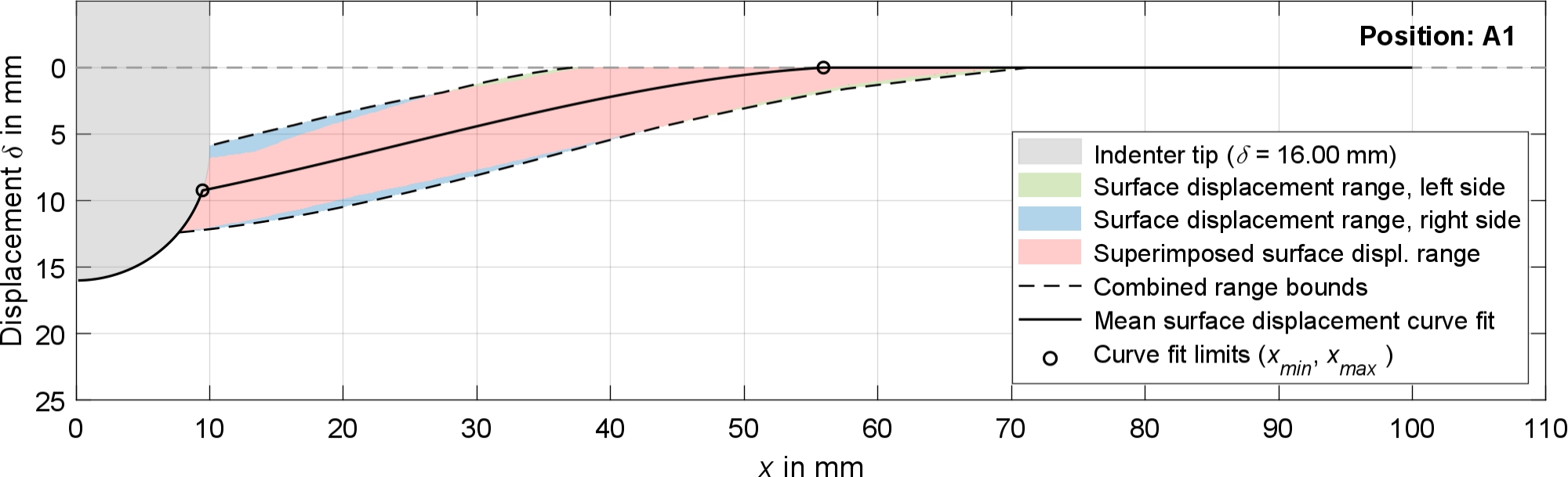

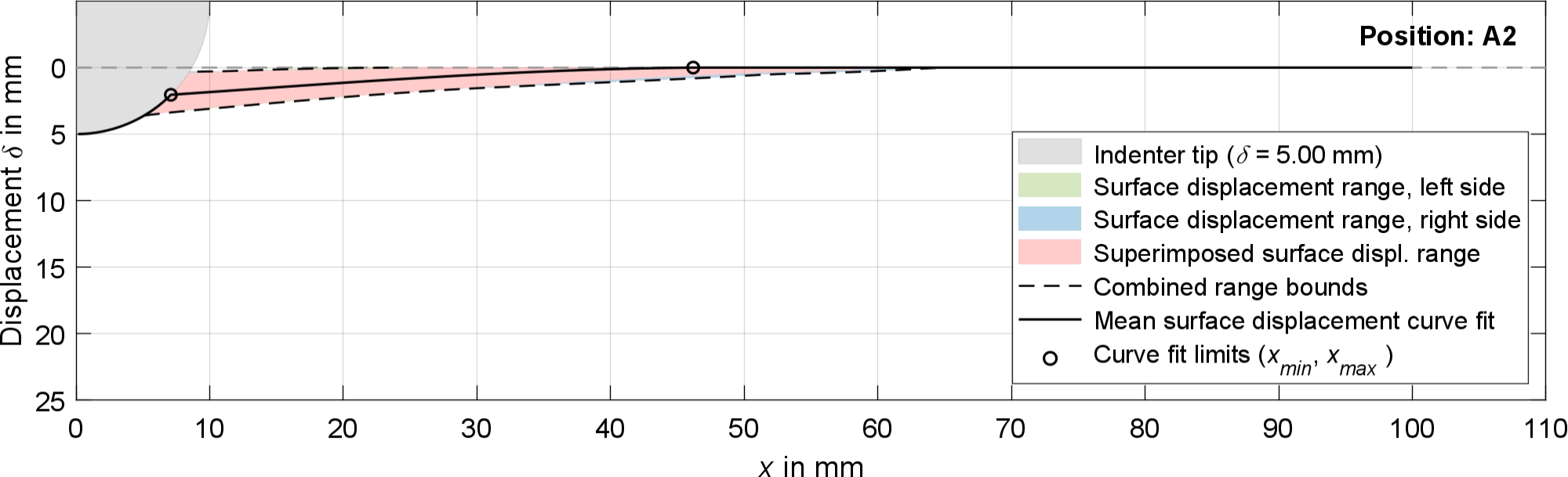

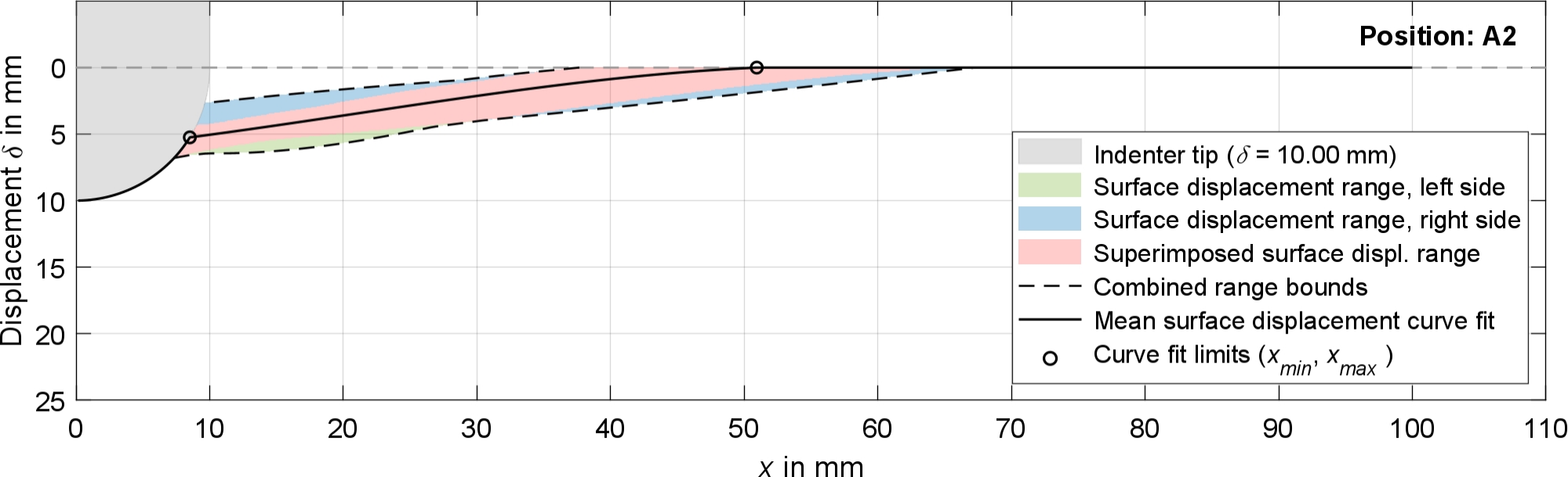

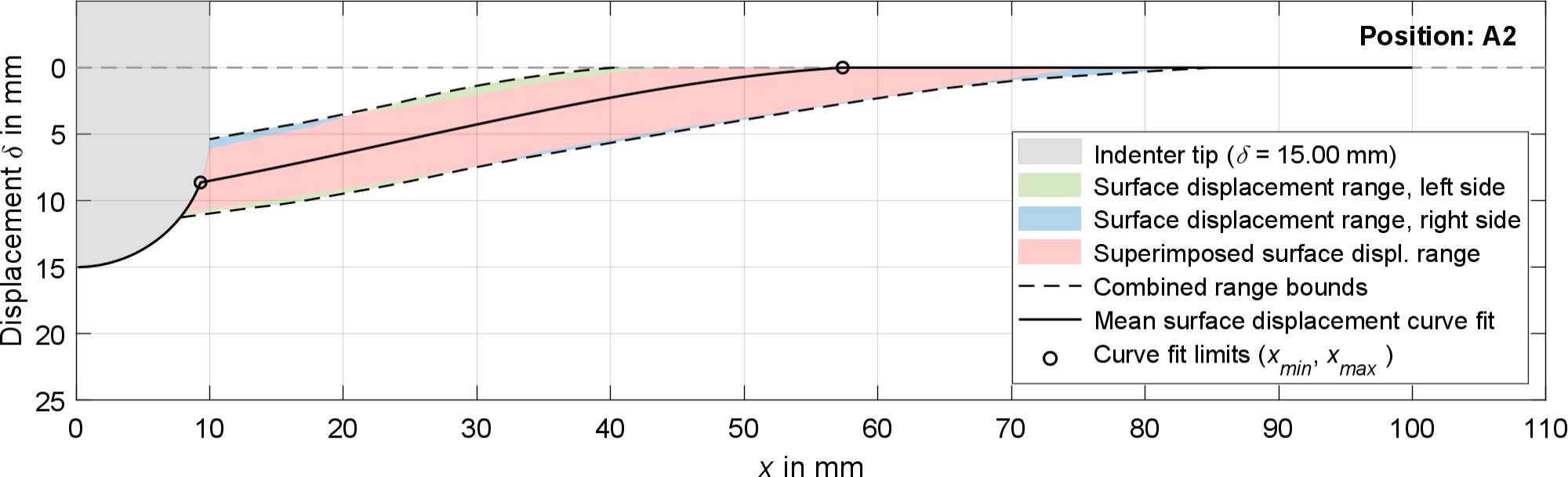

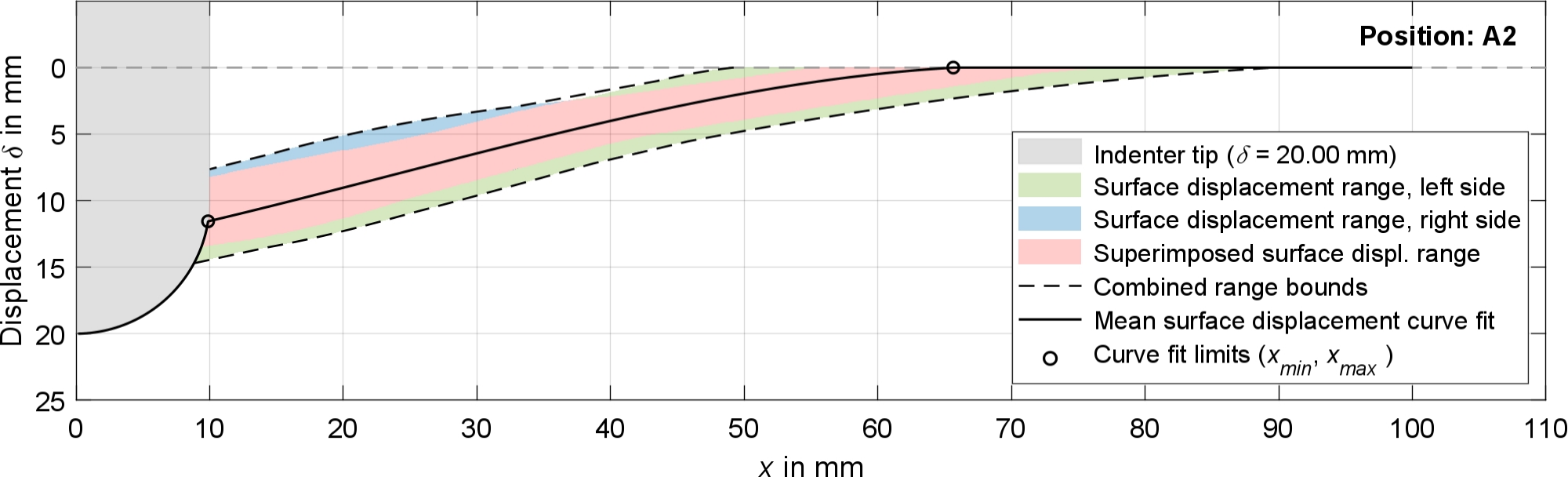

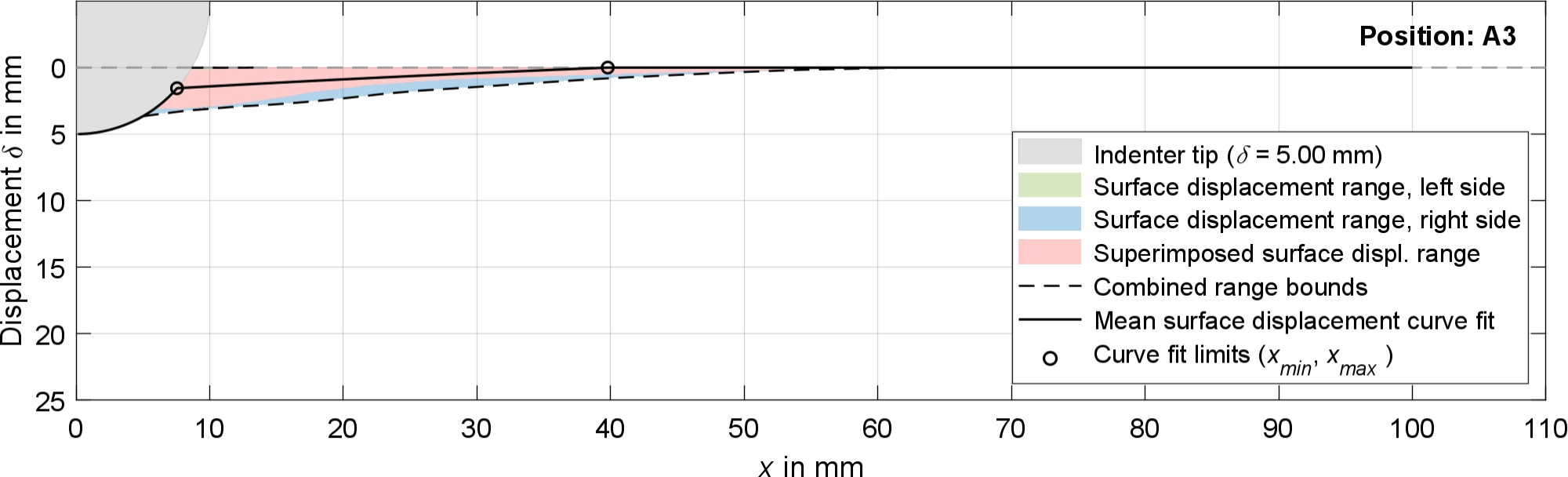

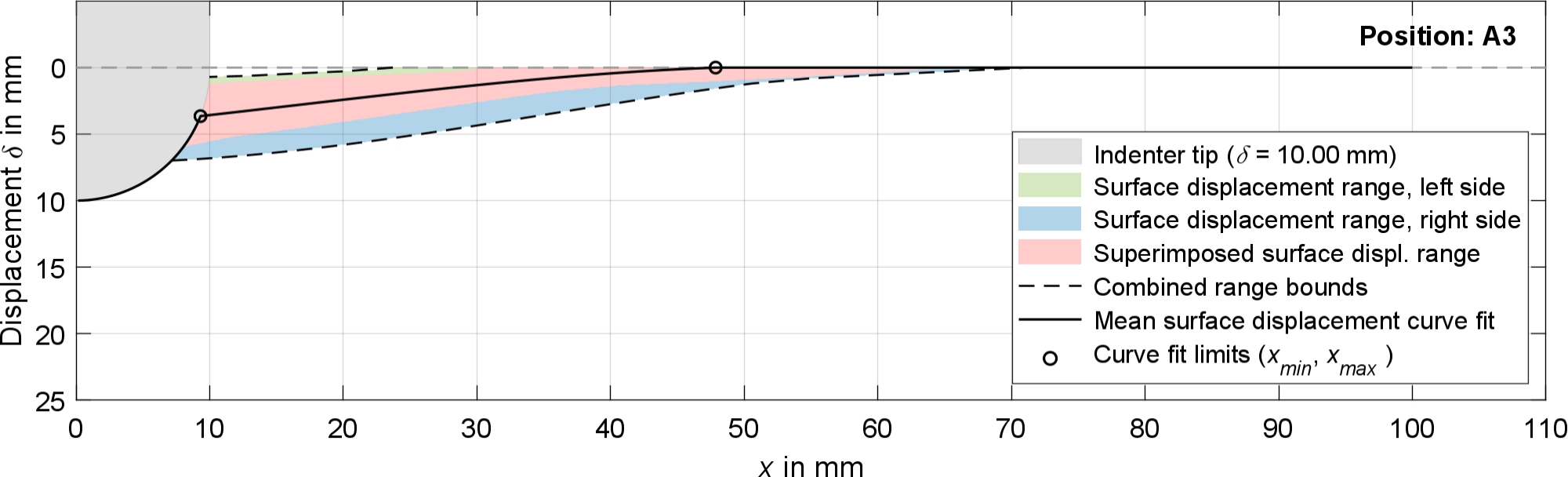

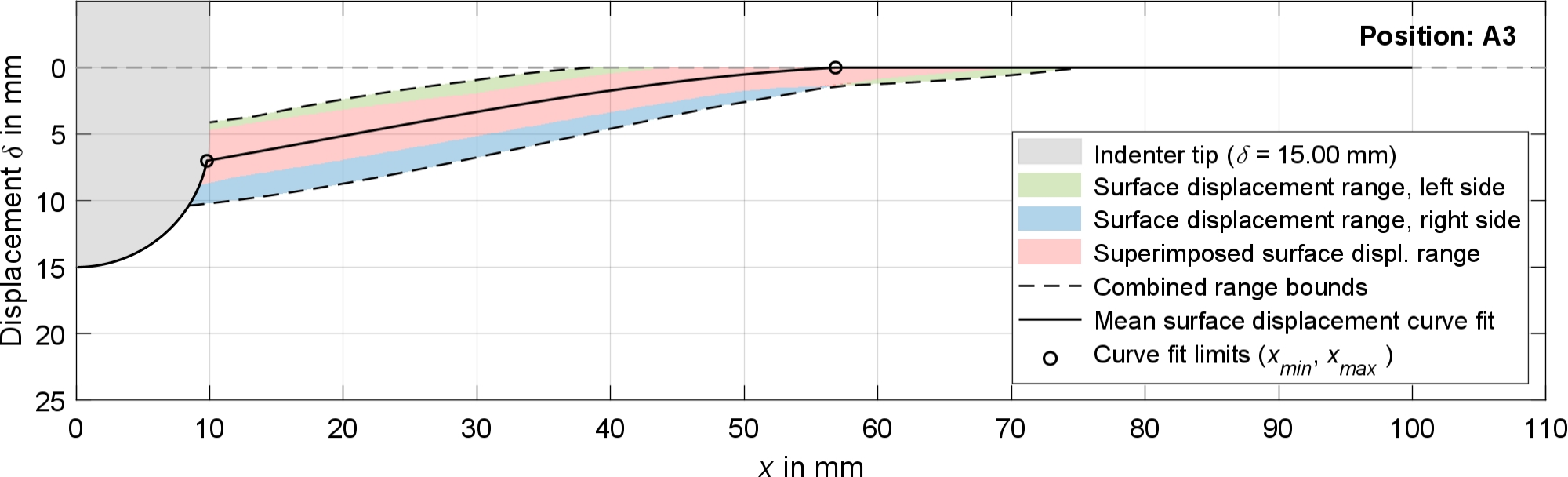

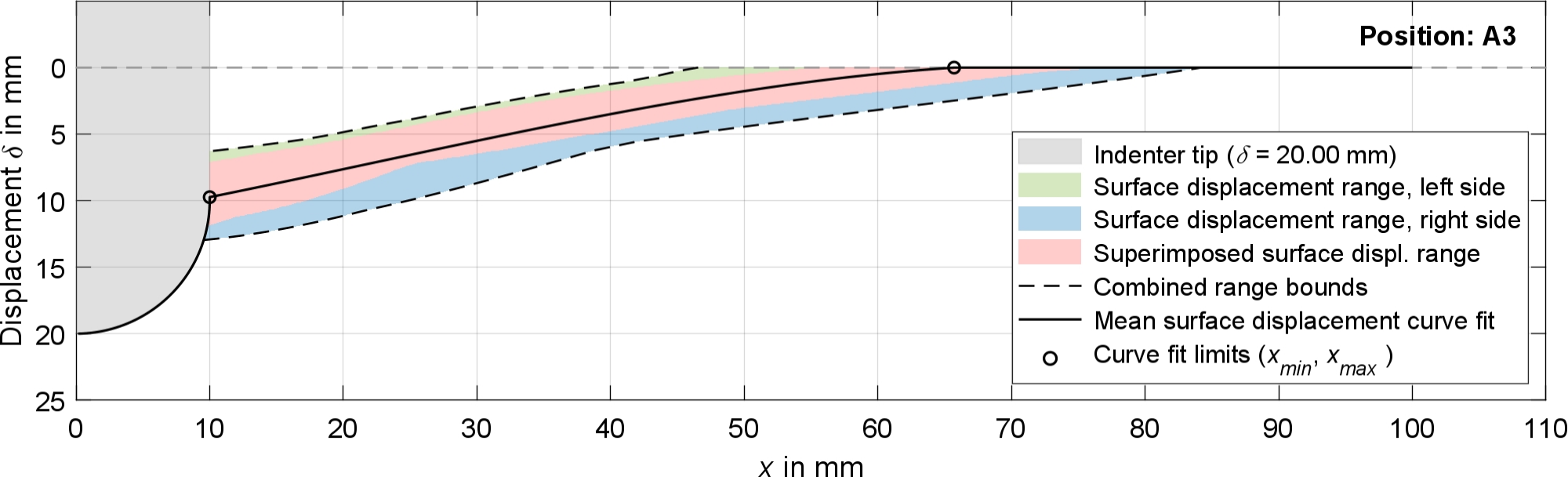

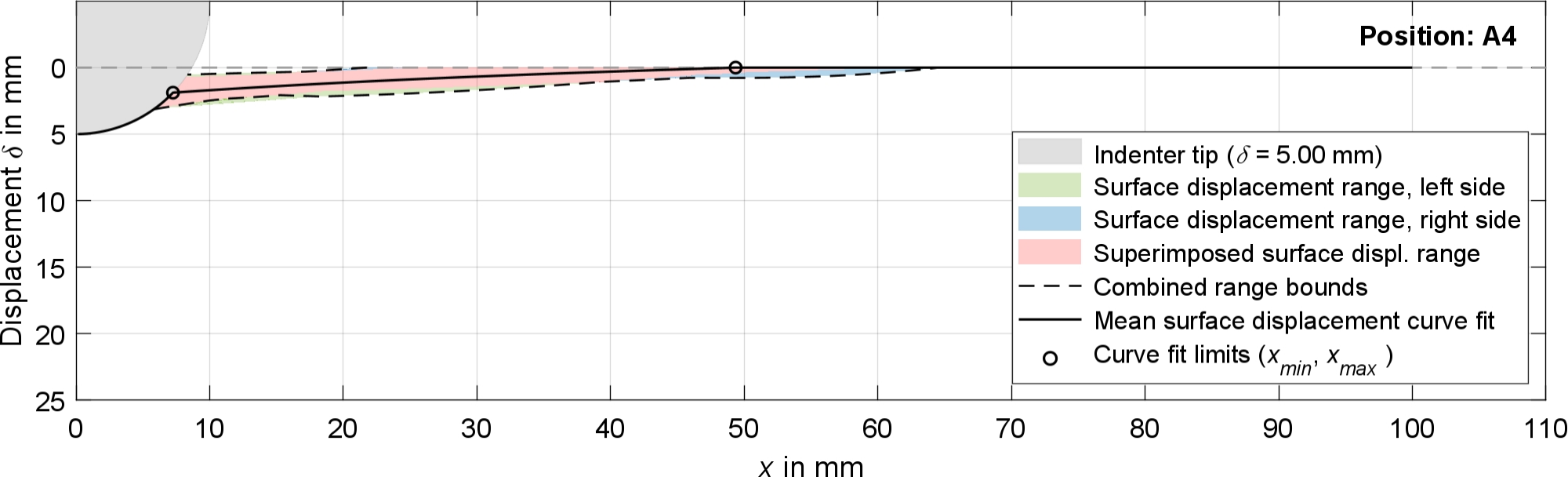

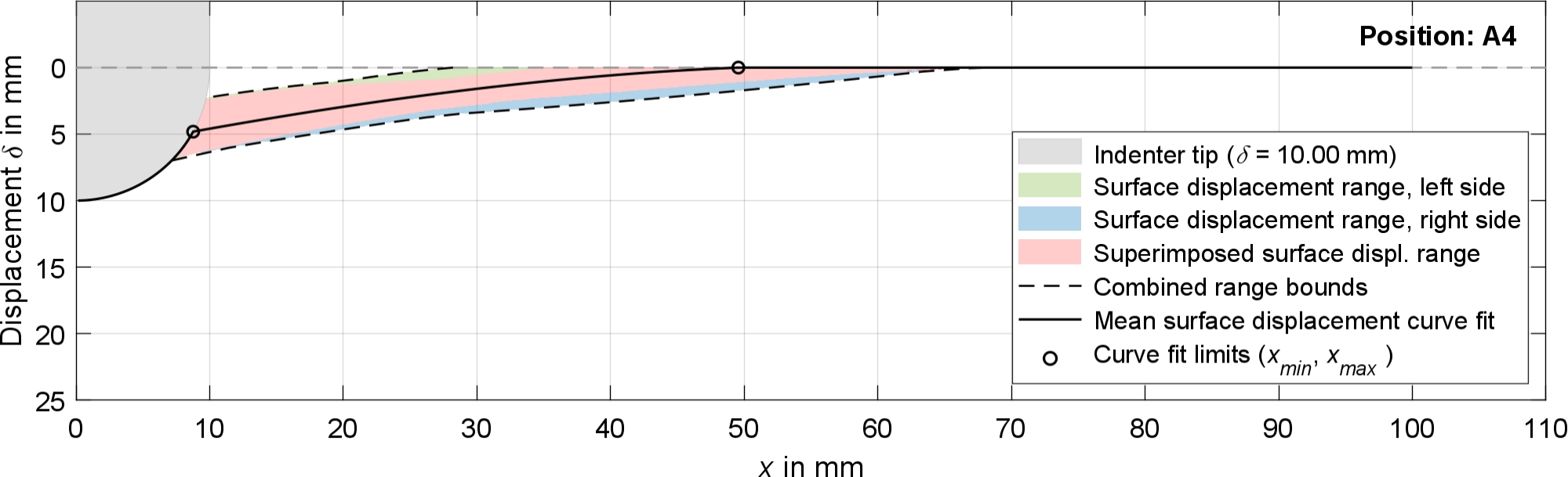

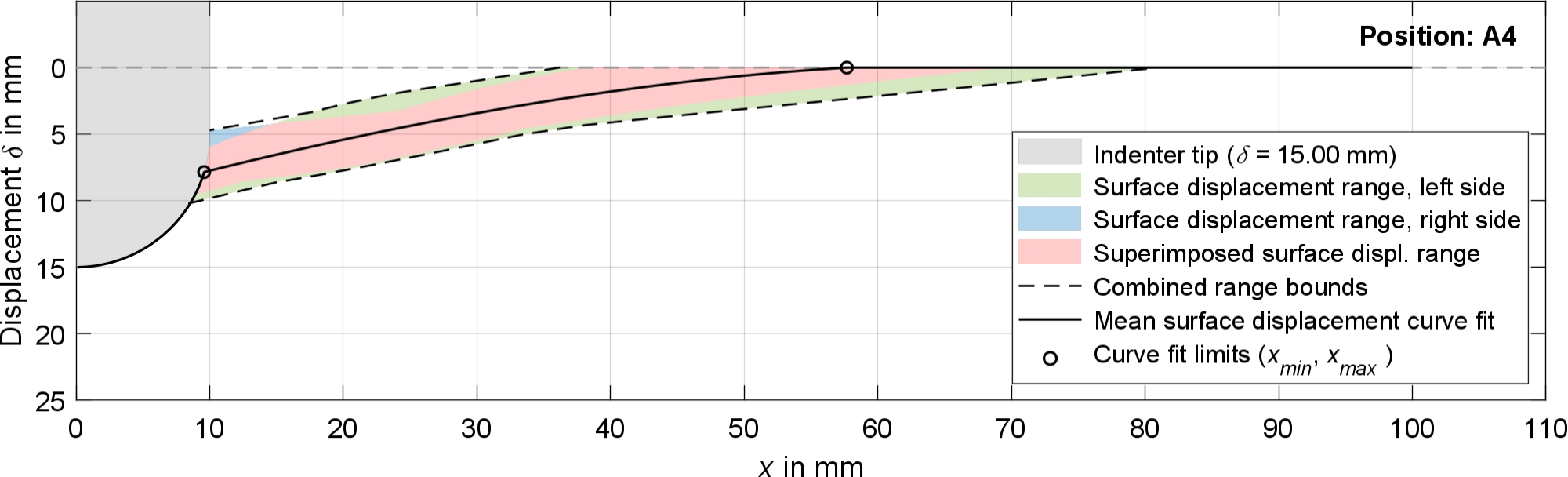

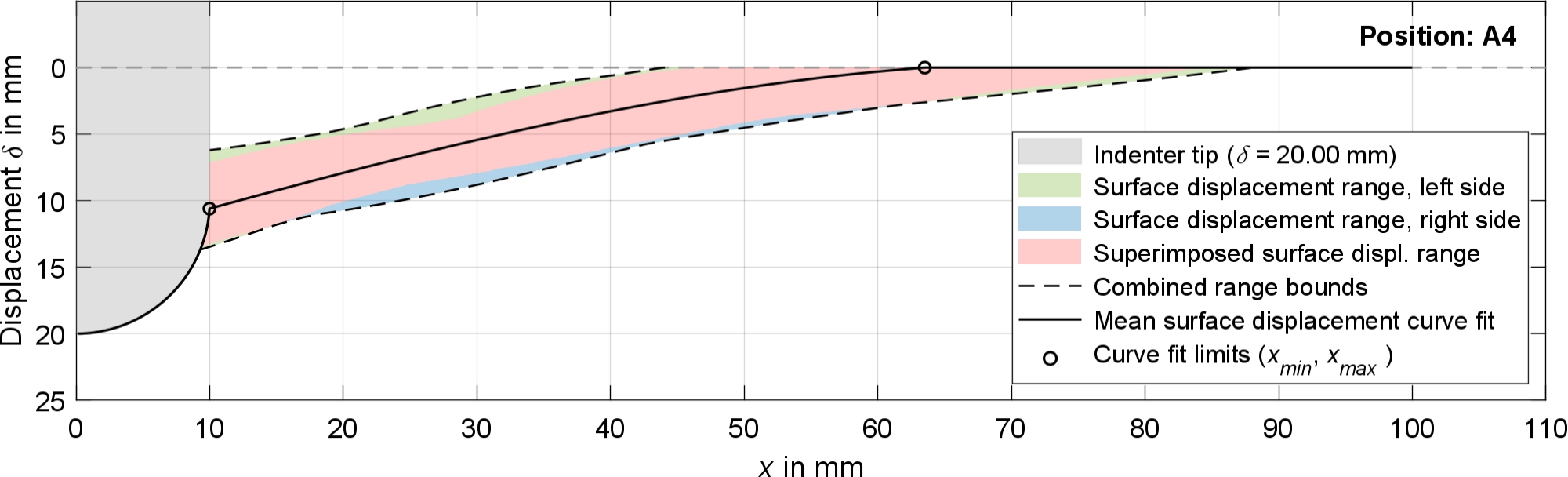

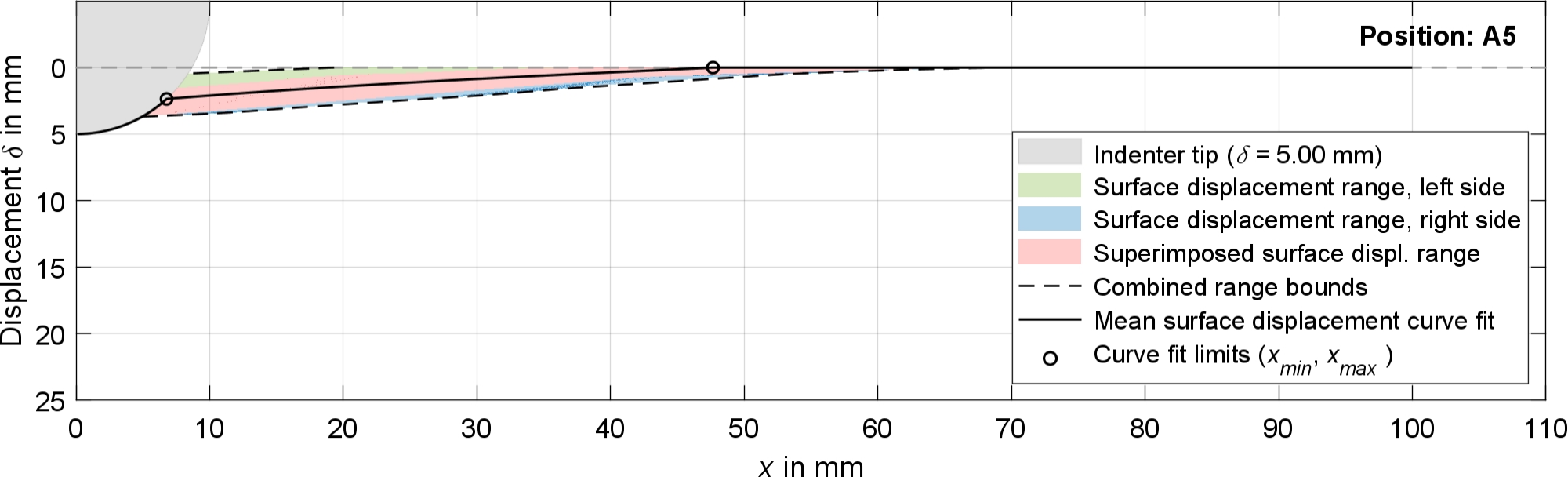

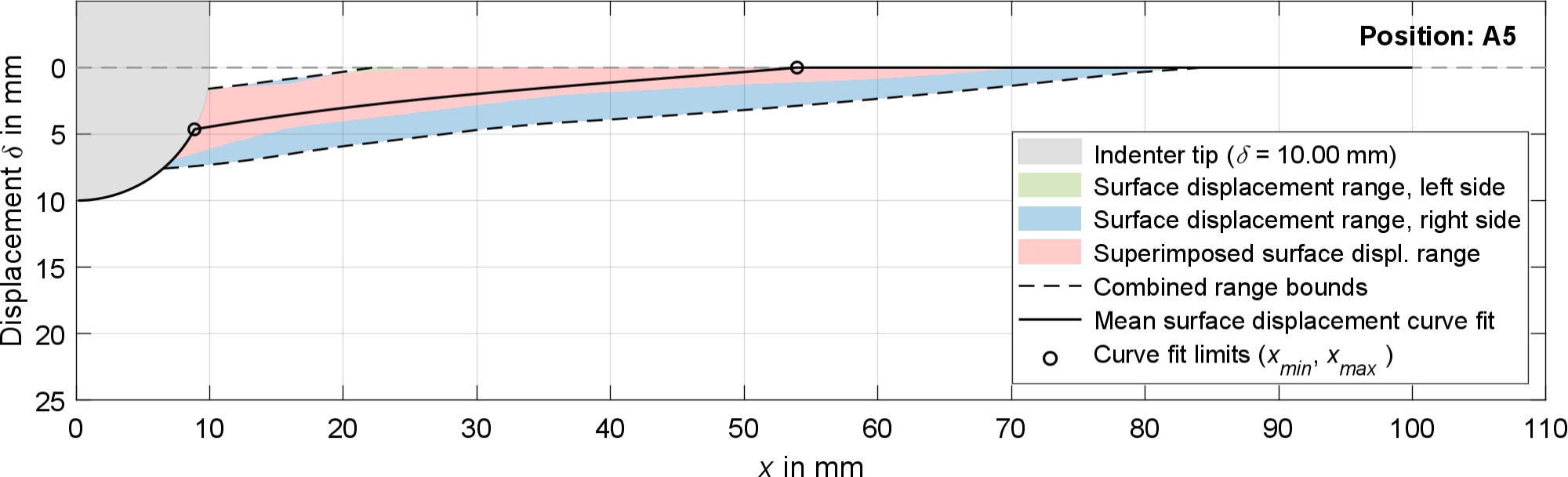

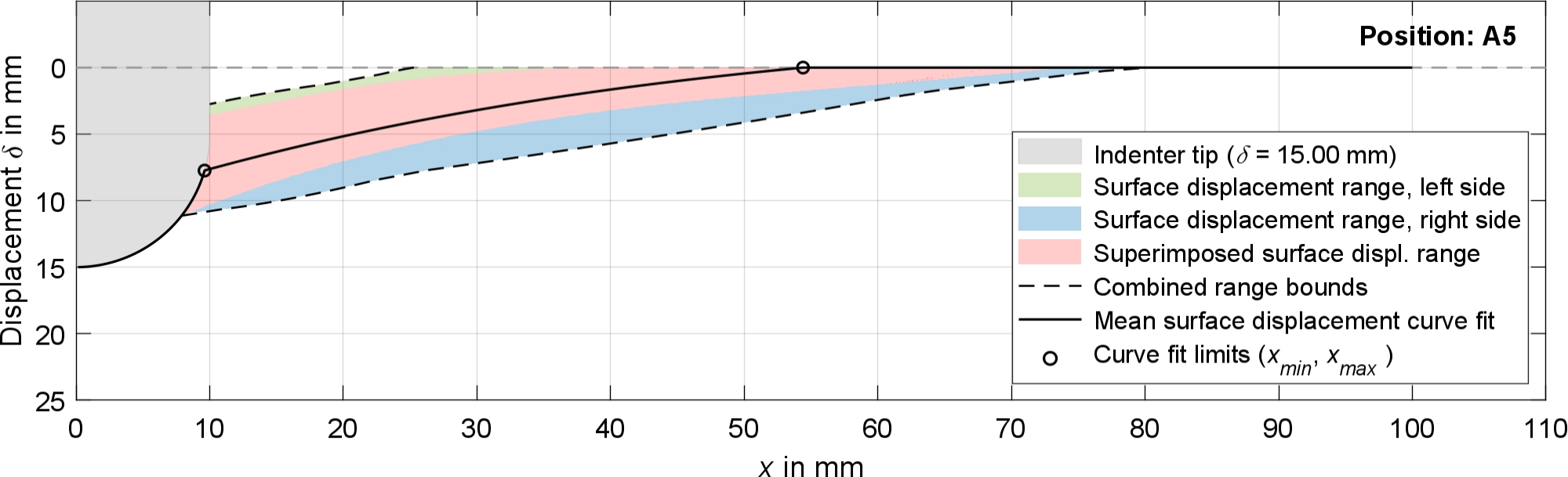

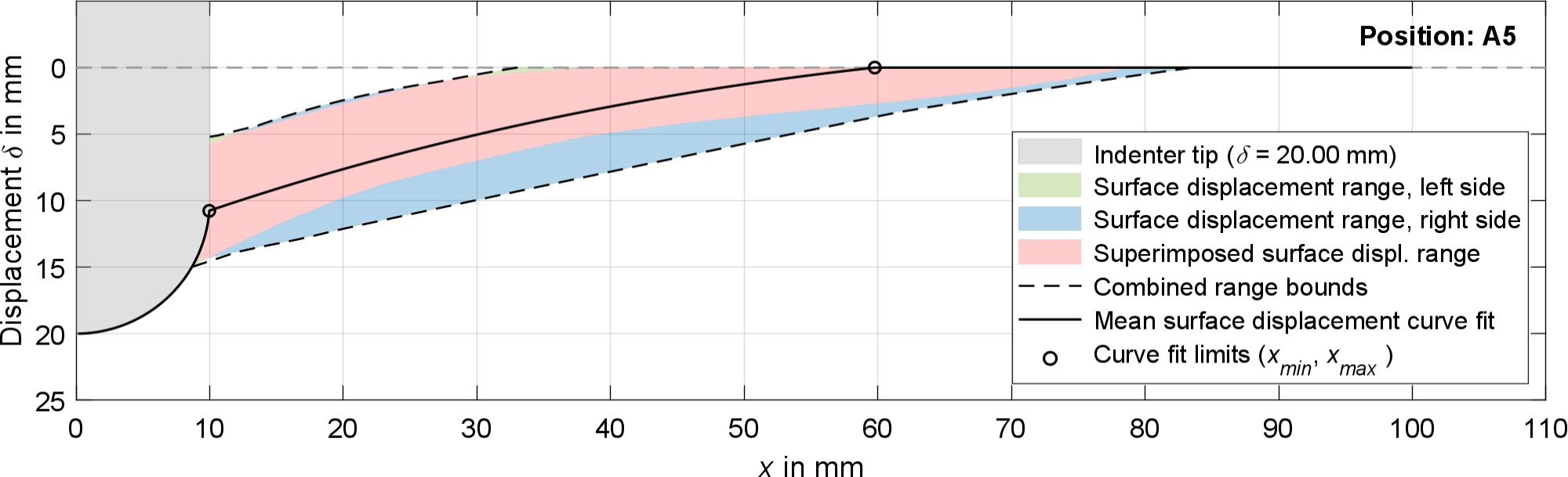

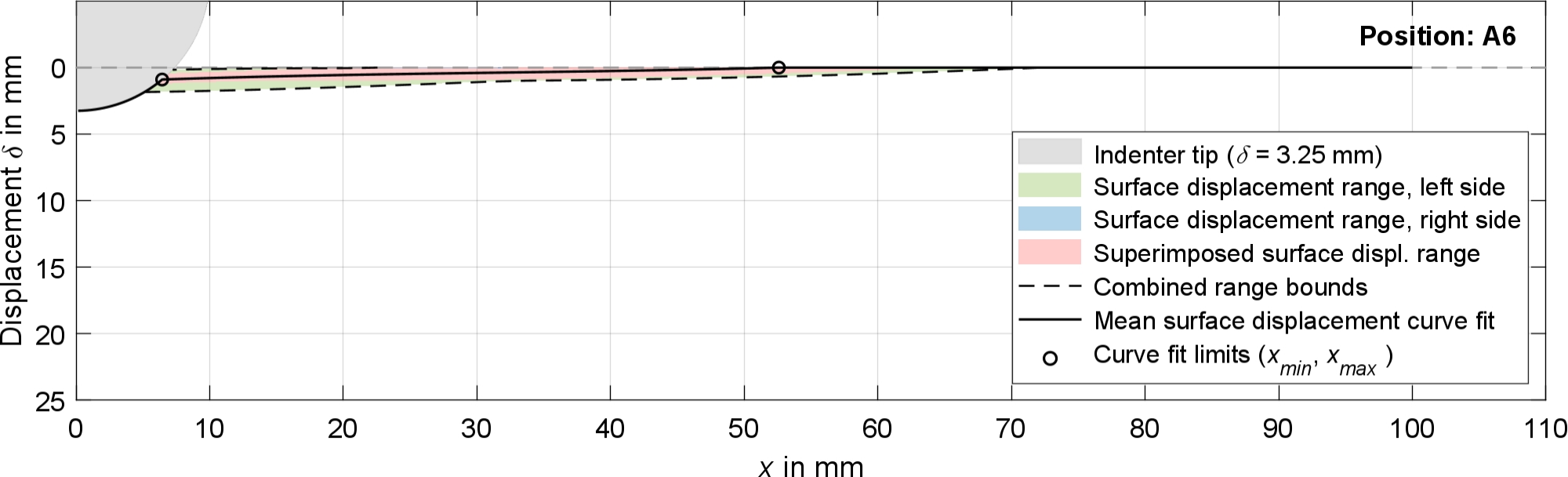

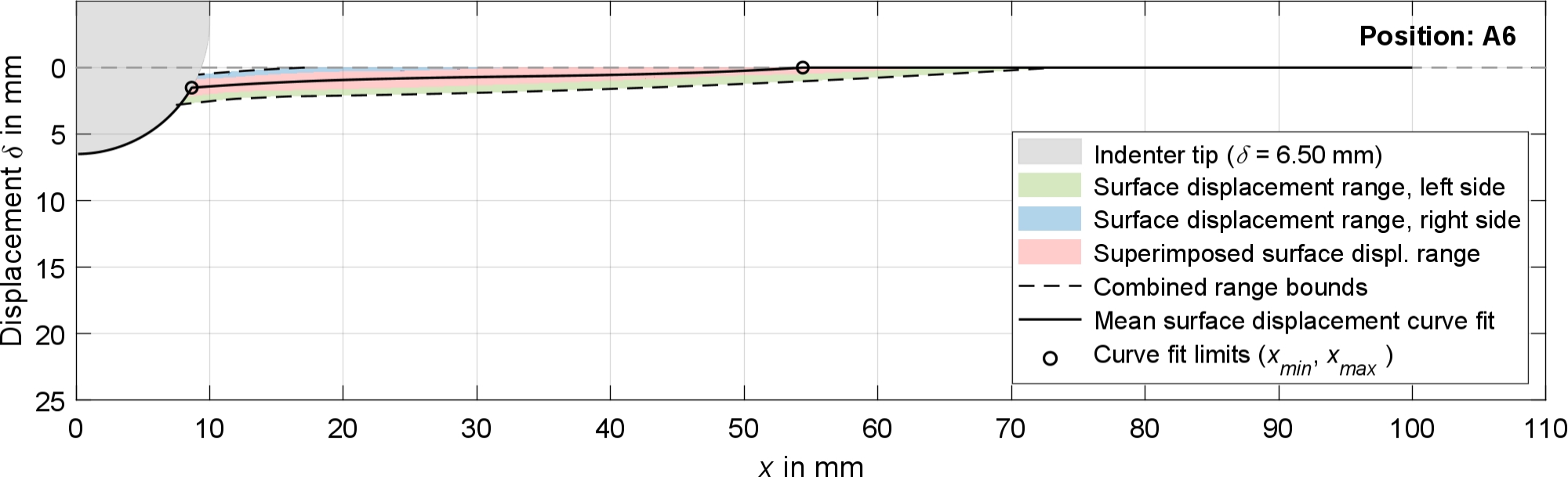

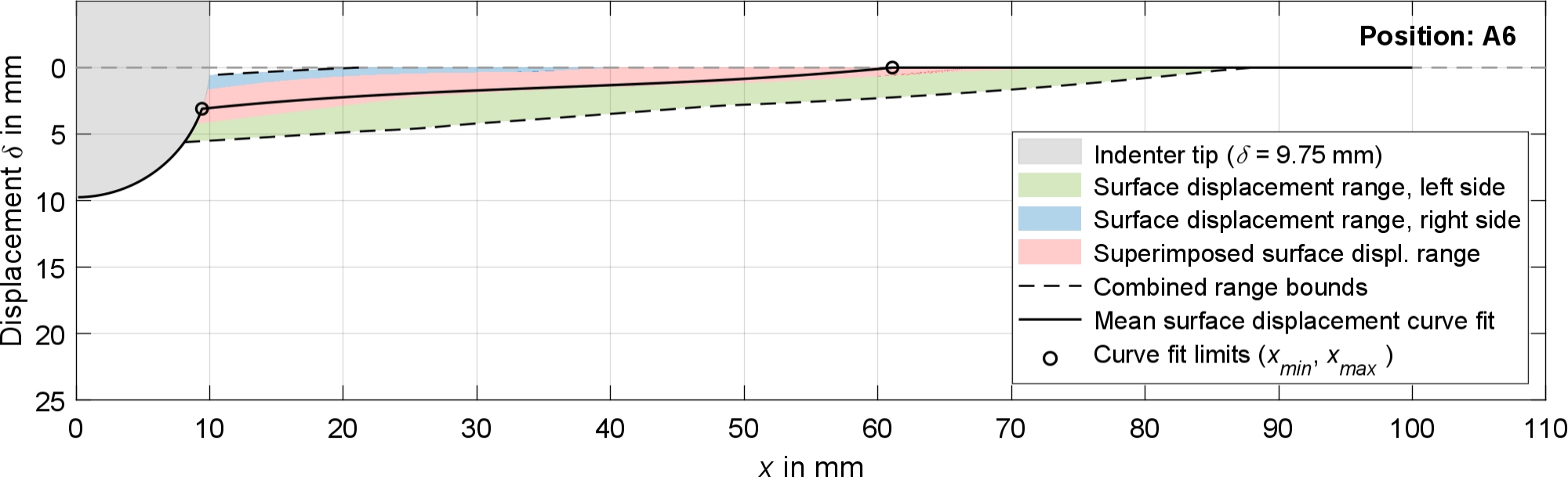

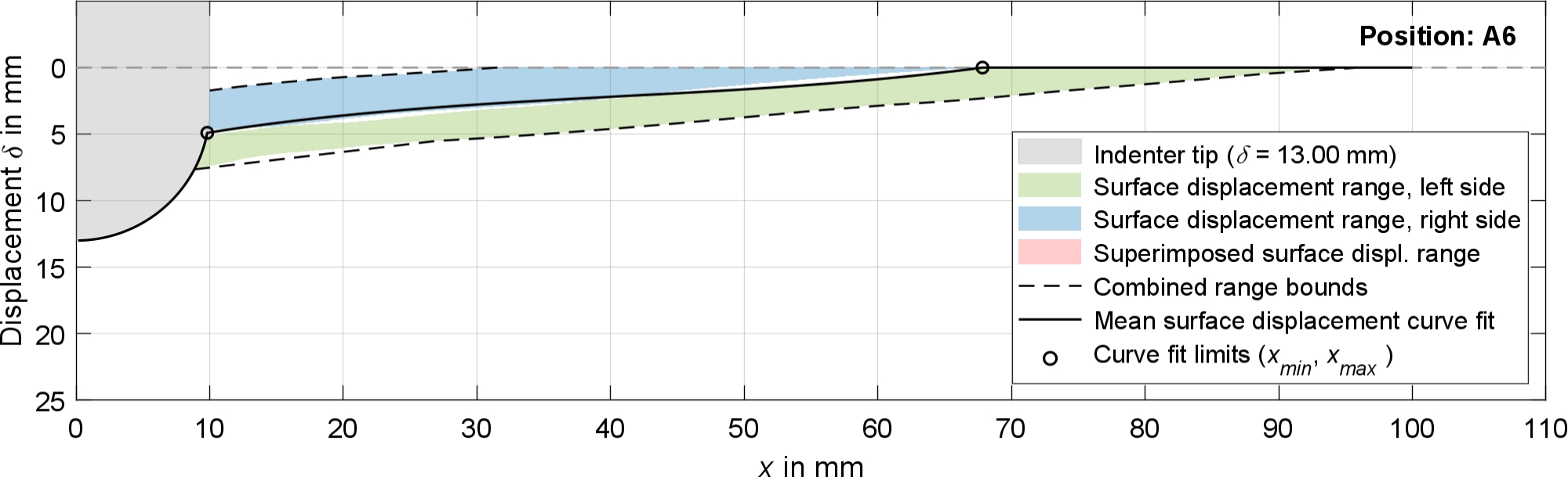

Supplement: Supplementary file 2 [file DataSheet4.PDF]

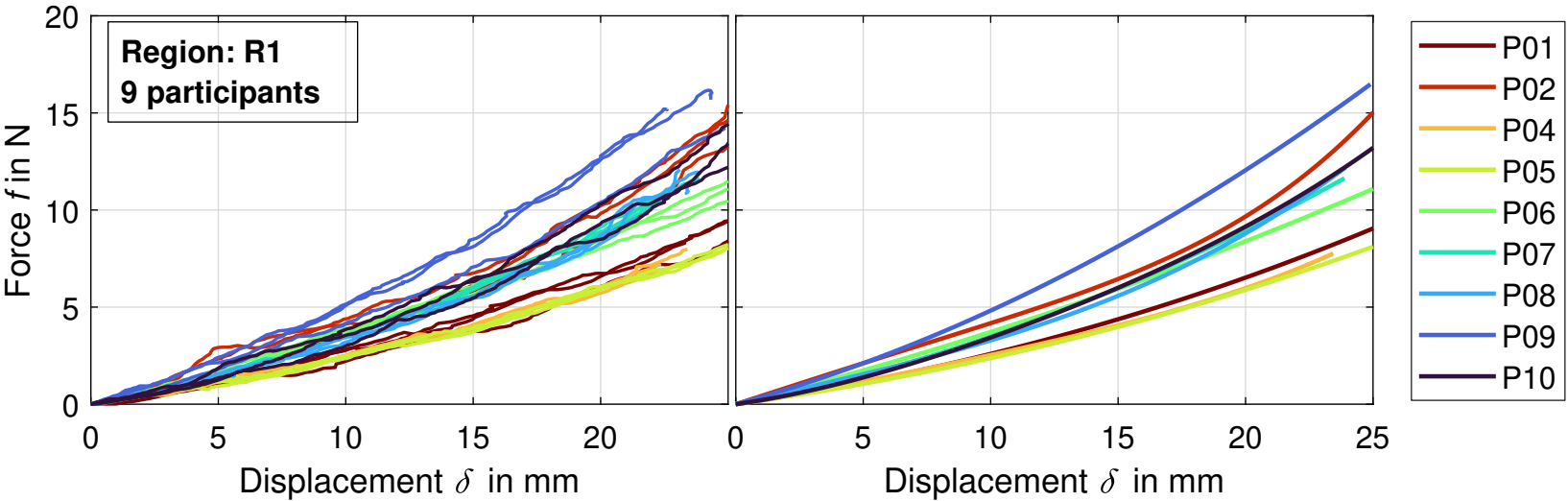

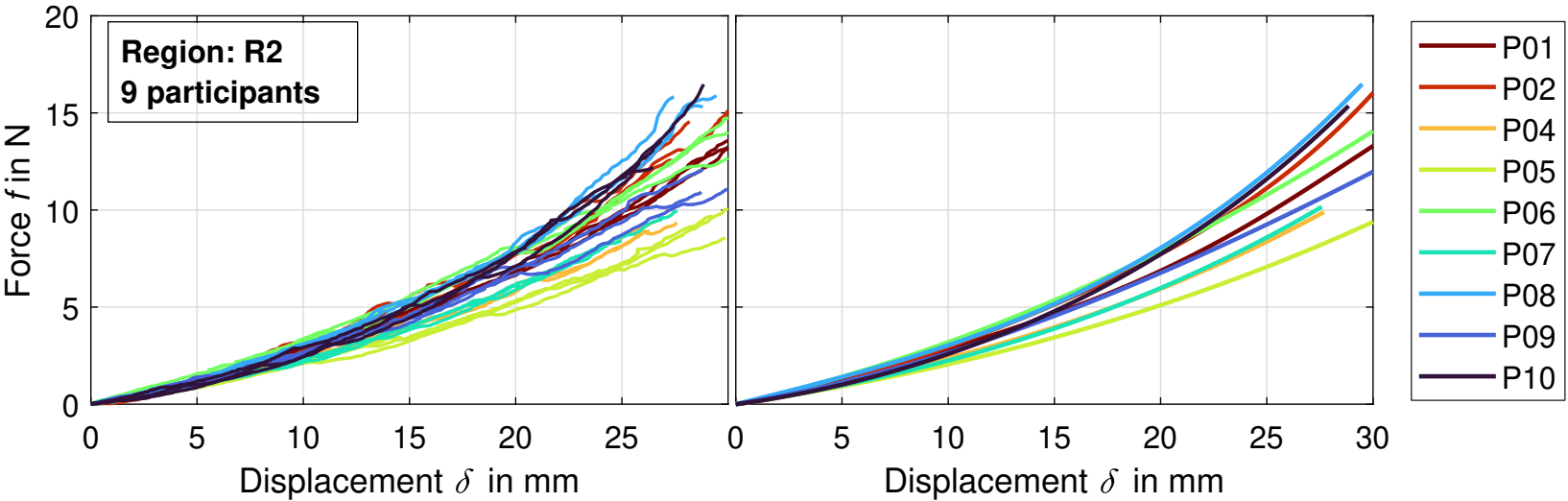

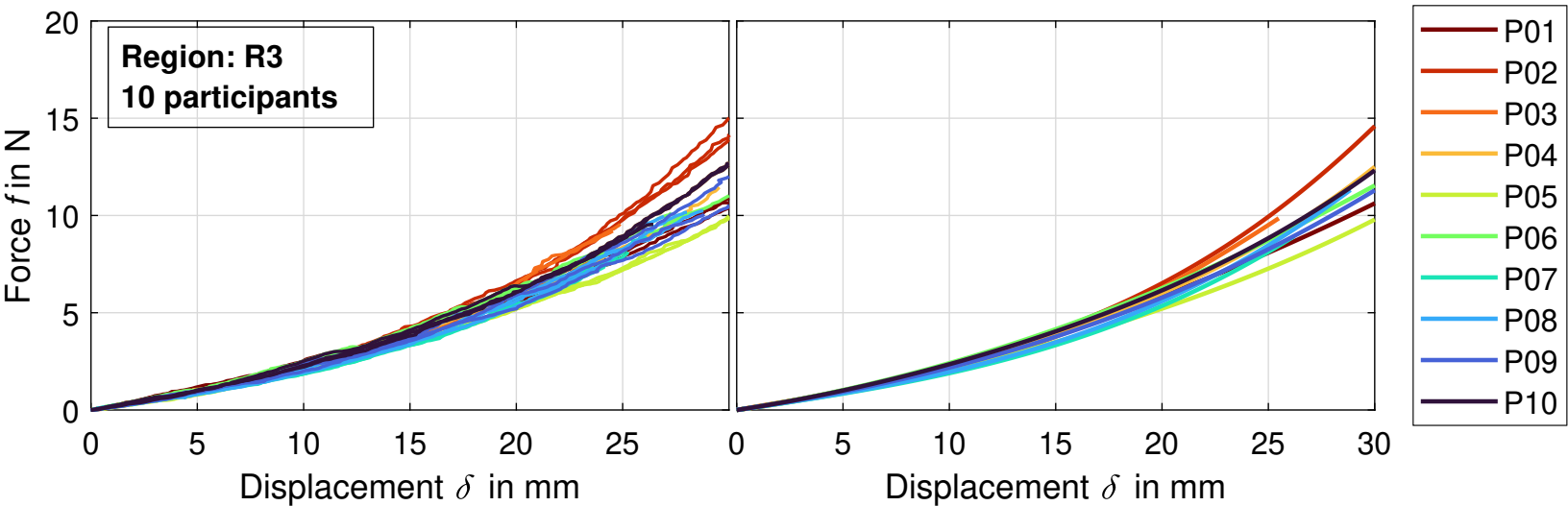

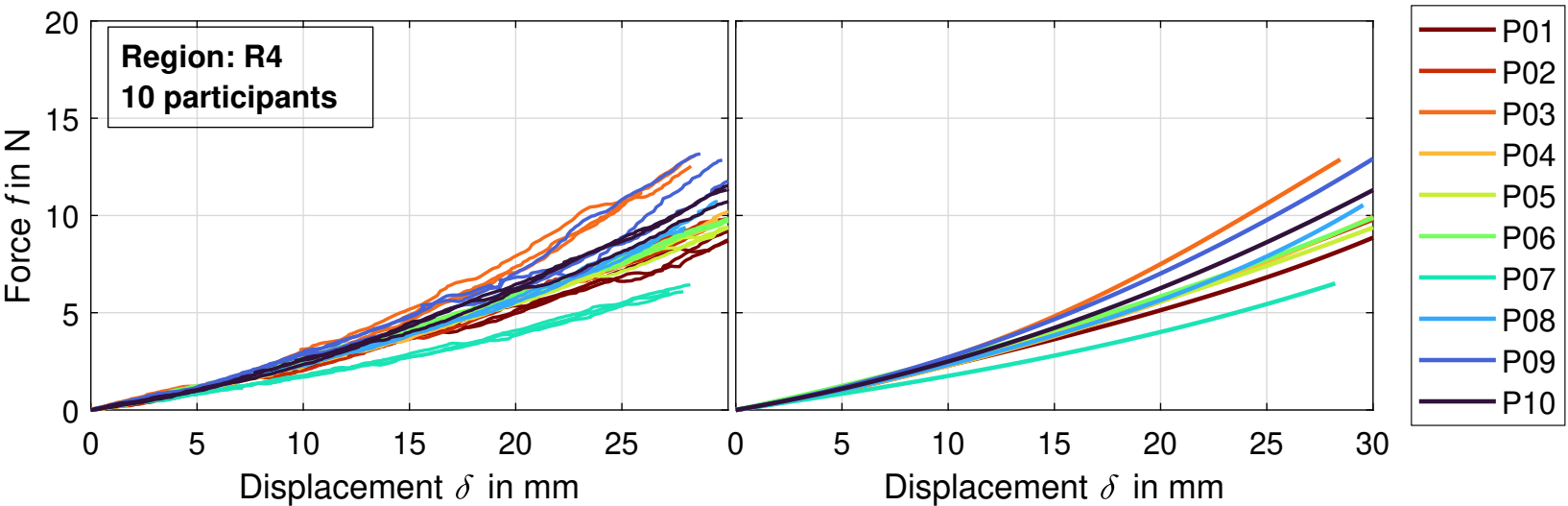

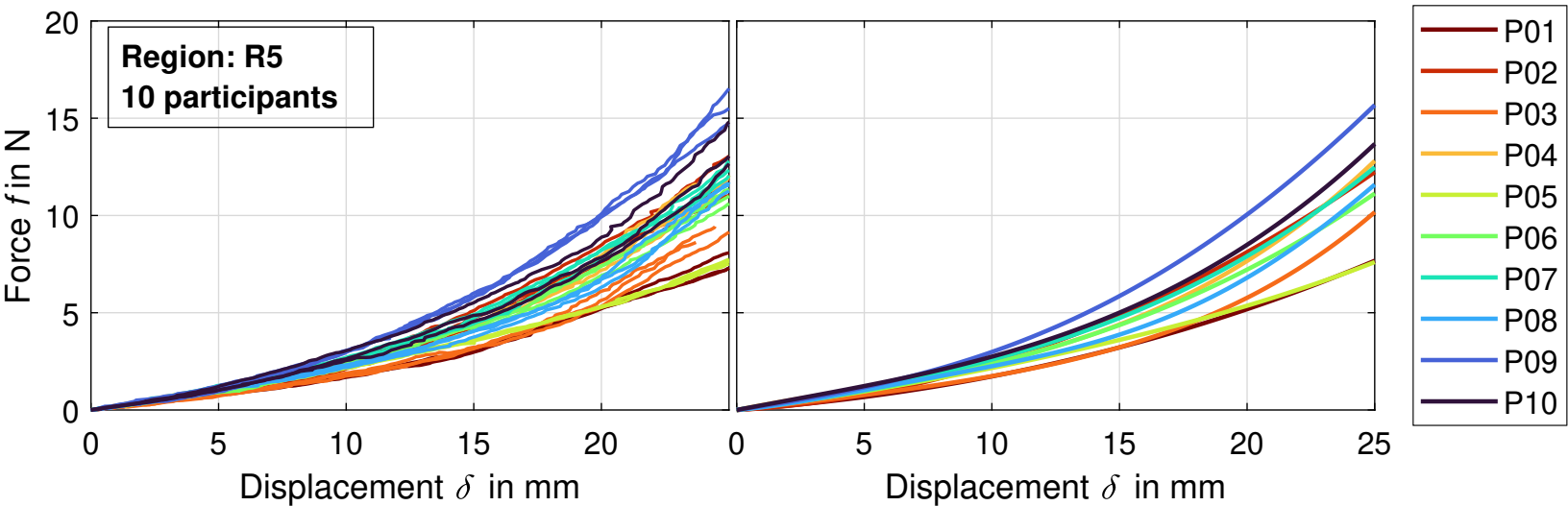

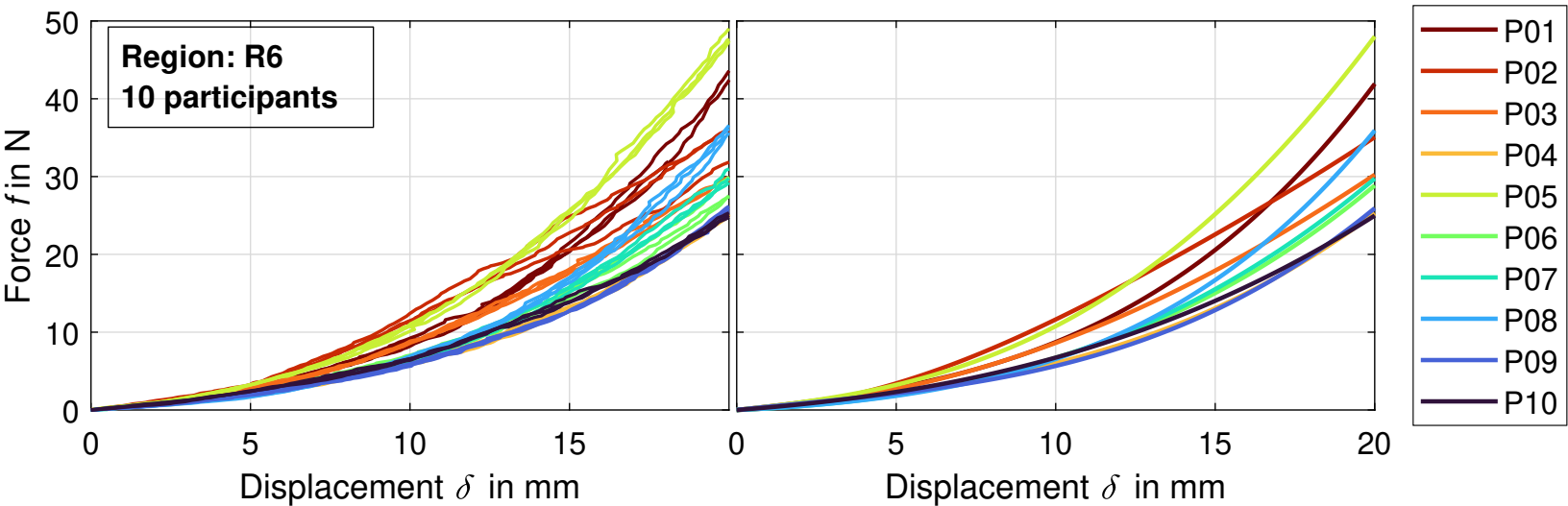

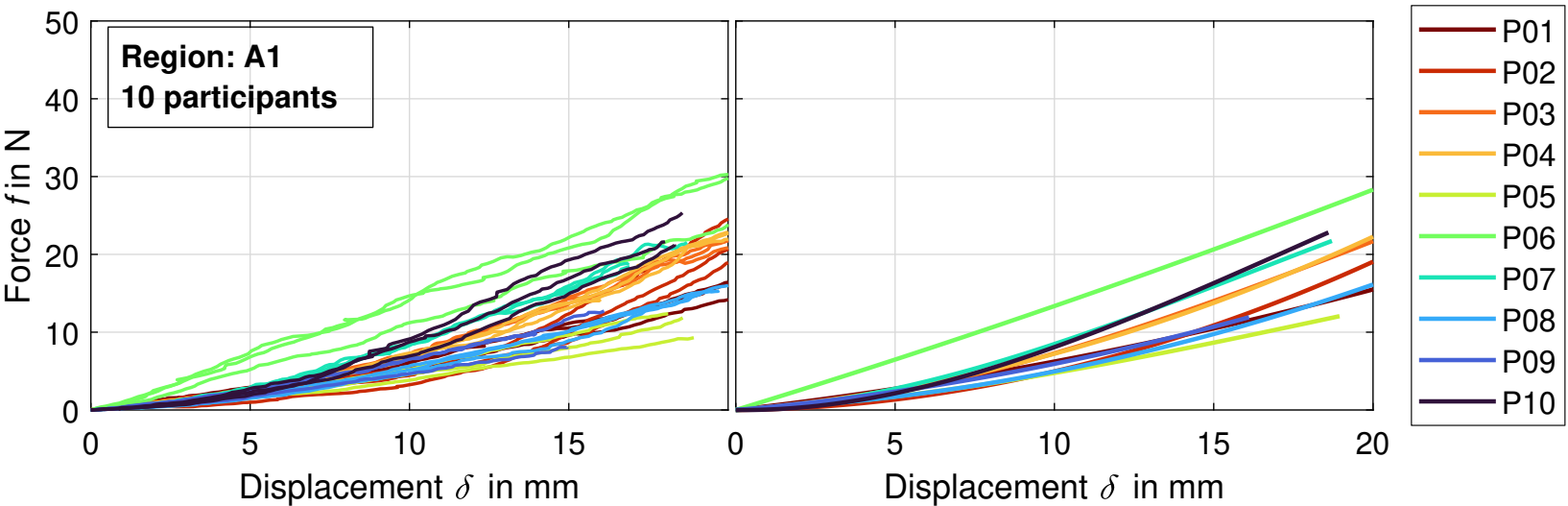

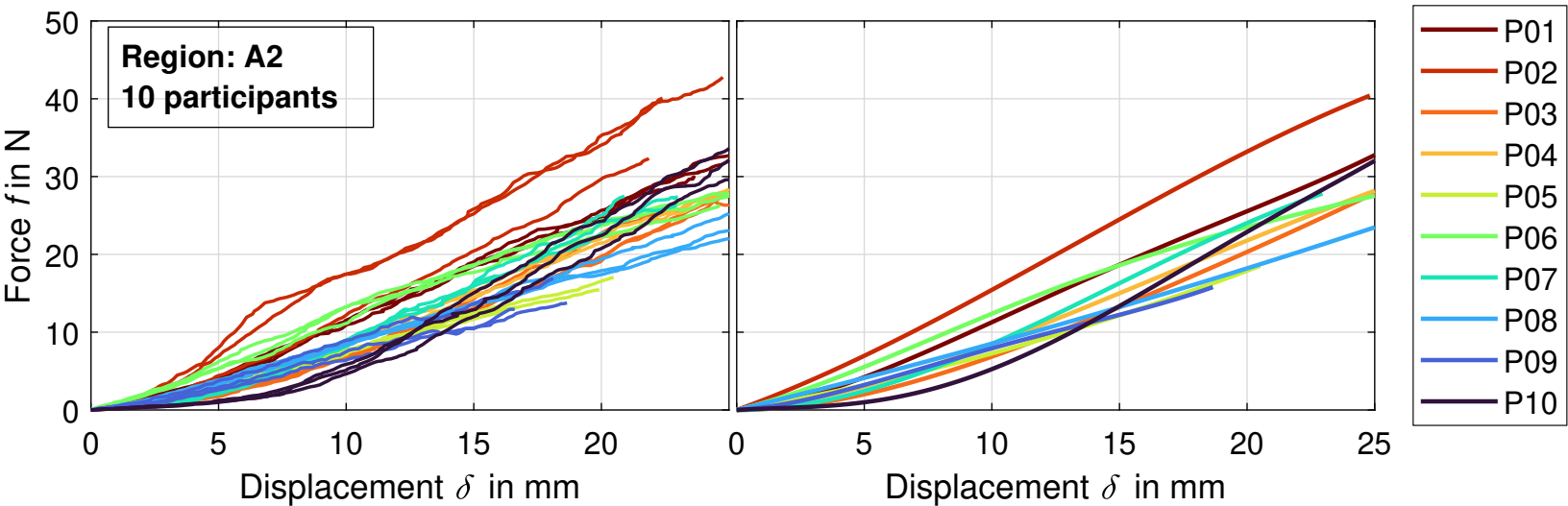

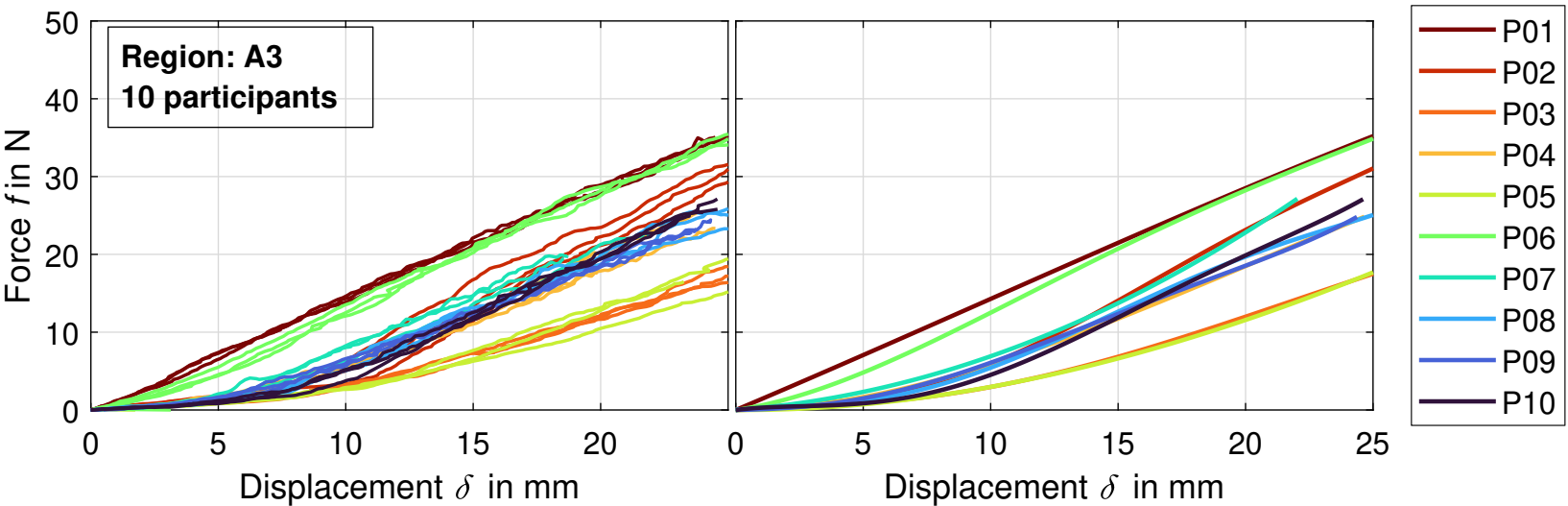

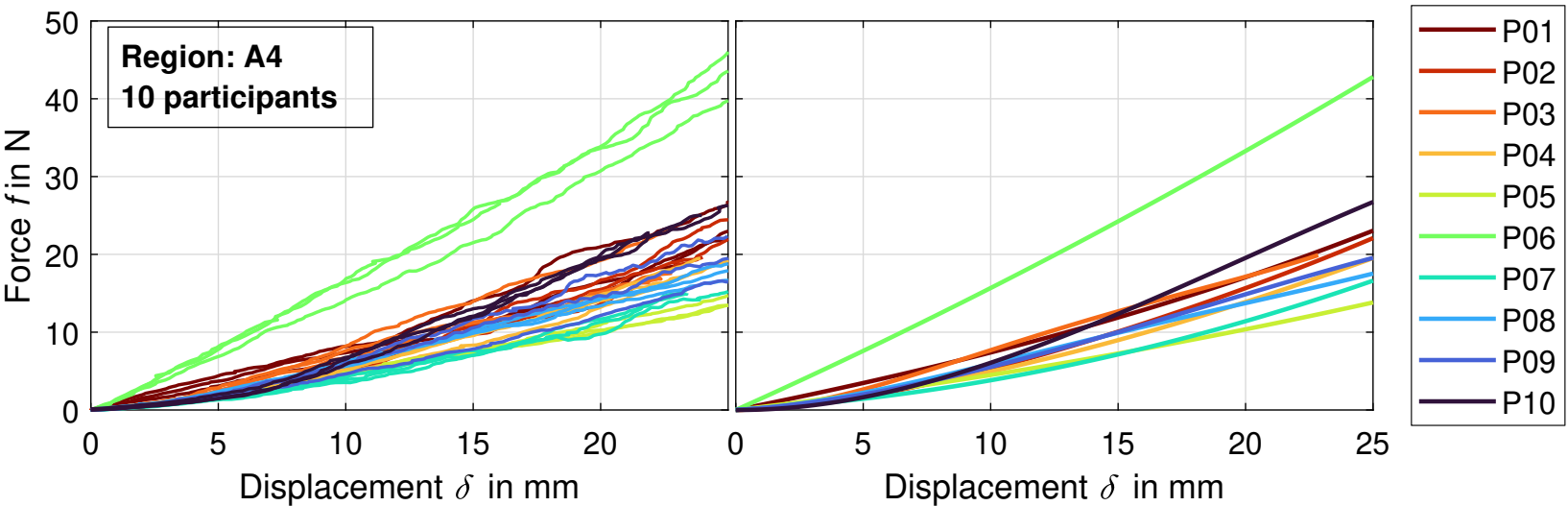

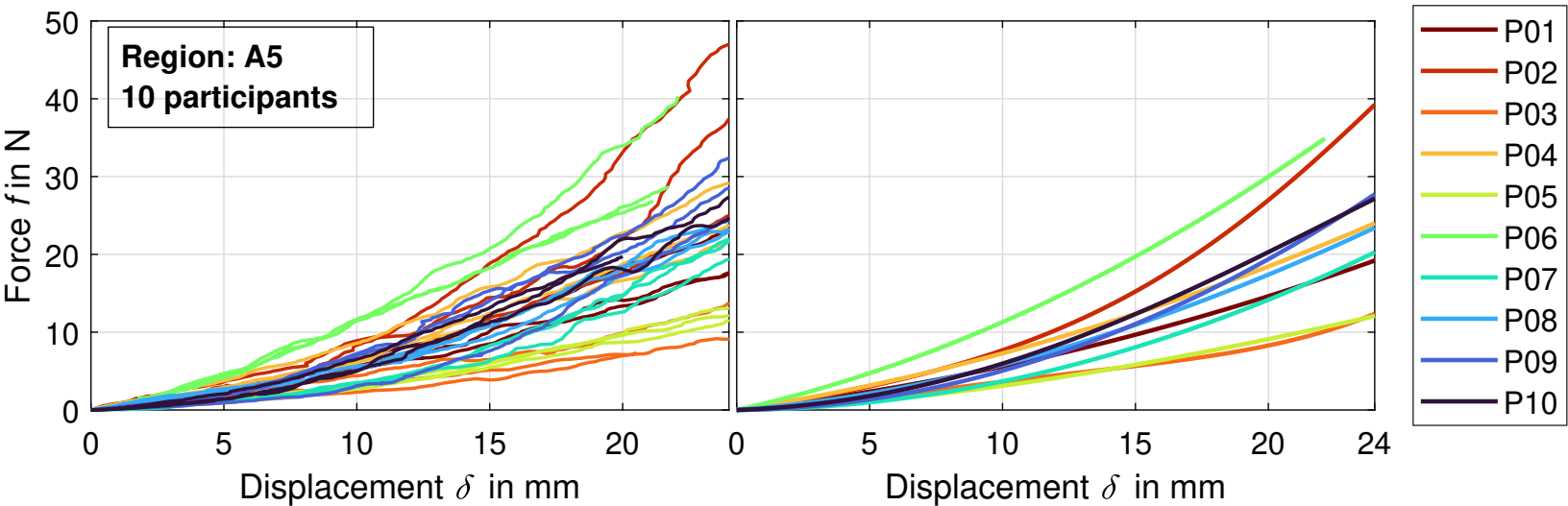

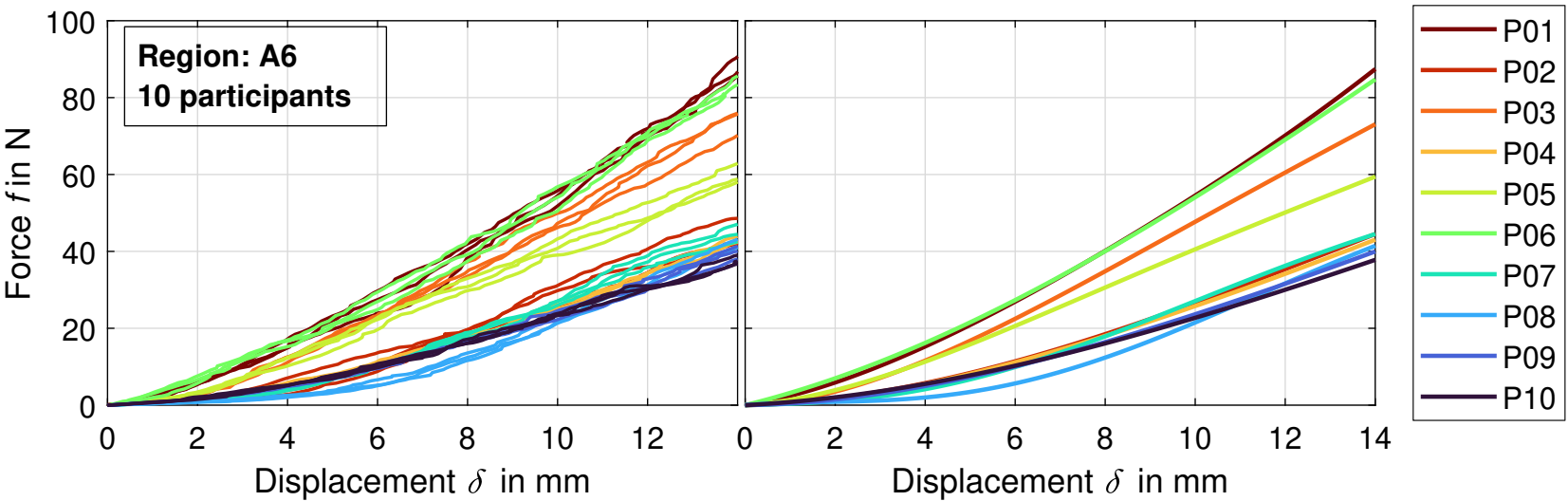

Supplement: Supplementary file 3 [file DataSheet3.PDF]
